# SERENA: Particle Instrument Suite for Determining the Sun-Mercury Interaction from BepiColombo

**DOI:** 10.1007/s11214-020-00787-3

**Published:** 2021-01-12

**Authors:** S. Orsini, S. A. Livi, H. Lichtenegger, S. Barabash, A. Milillo, E. De Angelis, M. Phillips, G. Laky, M. Wieser, A. Olivieri, C. Plainaki, G. Ho, R. M. Killen, J. A. Slavin, P. Wurz, J.-J. Berthelier, I. Dandouras, E. Kallio, S. McKenna-Lawlor, S. Szalai, K. Torkar, O. Vaisberg, F. Allegrini, I. A. Daglis, C. Dong, C. P. Escoubet, S. Fatemi, M. Fränz, S. Ivanovski, N. Krupp, H. Lammer, François Leblanc, V. Mangano, A. Mura, H. Nilsson, J. M. Raines, R. Rispoli, M. Sarantos, H. T. Smith, K. Szego, A. Aronica, F. Camozzi, A. M. Di Lellis, G. Fremuth, F. Giner, R. Gurnee, J. Hayes, H. Jeszenszky, F. Tominetti, B. Trantham, J. Balaz, W. Baumjohann, D. Brienza, U. Bührke, M. D. Bush, M. Cantatore, S. Cibella, L. Colasanti, G. Cremonese, L. Cremonesi, M. D’Alessandro, D. Delcourt, M. Delva, M. Desai, M. Fama, M. Ferris, H. Fischer, A. Gaggero, D. Gamborino, P. Garnier, W. C. Gibson, R. Goldstein, M. Grande, V. Grishin, D. Haggerty, M. Holmström, I. Horvath, K.-C. Hsieh, A. Jacques, R. E. Johnson, A. Kazakov, K. Kecskemety, H. Krüger, C. Kürbisch, F. Lazzarotto, Frederic Leblanc, M. Leichtfried, R. Leoni, A. Loose, D. Maschietti, S. Massetti, F. Mattioli, G. Miller, D. Moissenko, A. Morbidini, R. Noschese, F. Nuccilli, C. Nunez, N. Paschalidis, S. Persyn, D. Piazza, M. Oja, J. Ryno, W. Schmidt, J. A. Scheer, A. Shestakov, S. Shuvalov, K. Seki, S. Selci, K. Smith, R. Sordini, J. Svensson, L. Szalai, D. Toublanc, C. Urdiales, A. Varsani, N. Vertolli, R. Wallner, P. Wahlstroem, P. Wilson, S. Zampieri

**Affiliations:** 1 Institute of Space Astrophysics and Planetology, INAF, via del Fosso del Cavaliere 100, 00133 Rome, Italy; 2 Southwest Research Institute, San Antonio, TX USA; 3 Space Research Institute, Austrian Academy of Sciences, Graz, Austria; 4 Swedish Institute of Space Physics, Kiruna, Sweden; 5 Italian Space Agency, Roma, Italy; 6 The Johns Hopkins University Applied Physics Laboratory, Laurel, MD 20723 USA; 7 NASA/Goddard Space Flight Center, Greenbelt, MD 20771 USA; 8 Department of Climate and Space Sciences and Engineering, University of Michigan, Ann Arbor, MI USA; 9 Physics Institute, University of Bern, Bern, Switzerland; 10 LATMOS/IPSL, CNRS, Sorbonne Université, Paris, France; 11 Institut de Recherche en Astrophysique et Planétologie, CNRS, CNES, Université de Toulouse, Toulouse, France; 12 School of Electrical Engineering, Department of Electronics and Nanoengineering, Aalto University, Helsinki, Finland; 13 Space Technology Ireland, Ltd., Maynooth, Co. Kildare Ireland; 14 Wigner Research Centre for Physics, Budapest, Hungary; 15 IKI Space Research Institute, Moscow, Russia; 16 Department of Physics, National and Kapodistrian University of Athens, Athens, Greece; 17 Department of Astrophysical Sciences and Princeton Plasma Physics Laboratory, Princeton University, Princeton, NJ USA; 18 ESA-ESTEC, Noordwijk, The Netherlands; 19 Max-Planck-Institut für Sonnensystemforschung, MPS, 37077 Göttingen, Germany; 20 Astronomical Observatory, INAF, Trieste, Italy; 21 OHB-Italia SpA, Milano, Italy; 22 AMDL srl, Roma, Italy; 23 Laboratory for Atmospheric and Space Physics, Boulder, CO USA; 24 Institute of Experimental Physics SAS, Slovak Academy of Sciences, 040 01 Košice, Slovakia; 25 Astronomical Observatory, INAF, Padova, Italy; 26 Istituto di Struttura della Materia (CNR-ISM), 00133 Roma, Italy; 27 University of Orleans, Orleans, France; 28 Comisión Nacional de Energía Atómica, cnea, Centro Atómico Bariloche, Bariloche, Argentina; 29 Aberystwyth University, Aberystwyth, Ceredigion SY23 3FL UK; 30 University of Arizona, Tucson, AZ USA; 31 University of Virginia, Charlottesville, VA 22904 USA; 32 LPP, École polytechnique, 91128 Palaiseau Cedex, France; 33 PRISMA srl., Roma, Italy; 34 Istituto Fotonica e Nanotecnologie, CNR-IFN, Roma, Italy; 35 Finnish Meteorological Institute FMI, Helsinki, Finland; 36 TOFWERK, Thun, Switzerland; 37 Department of Earth and Planetary Science, Graduate School of Science, University of Tokyo, Tokyo, Japan; 38 EISCAT, Kiruna, Sweden; 39 Hellenic Space Center, Athens, Greece

**Keywords:** Mercury’s environment, Particle instrumentation, BepiColombo space mission

## Abstract

The ESA-JAXA BepiColombo mission to Mercury will provide simultaneous measurements from two spacecraft, offering an unprecedented opportunity to investigate magnetospheric and exospheric particle dynamics at Mercury as well as their interactions with solar wind, solar radiation, and interplanetary dust. The particle instrument suite SERENA (Search for Exospheric Refilling and Emitted Natural Abundances) is flying in space on-board the BepiColombo Mercury Planetary Orbiter (MPO) and is the only instrument for ion and neutral particle detection aboard the MPO. It comprises four independent sensors: ELENA for neutral particle flow detection, Strofio for neutral gas detection, PICAM for planetary ions observations, and MIPA, mostly for solar wind ion measurements. SERENA is managed by a System Control Unit located inside the ELENA box. In the present paper the scientific goals of this suite are described, and then the four units are detailed, as well as their major features and calibration results. Finally, the SERENA operational activities are shown during the orbital path around Mercury, with also some reference to the activities planned during the long cruise phase.

## Introduction

SERENA (Search for Exospheric Refilling and Emitted Natural Abundances, Orsini et al. 2010) is an experiment composed by four units on the MPO spacecraft that may be operated independently of each other.

ELENA (Emitted Low-Energy Neutral Atoms) covers the < 10 s eV – 5 keV integrated energy spectrum of neutral population from the surface and the close-to-planet environment. It has a high angular resolution and a nadir pointing 1-D field-of-view (perpendicular to the S/C orbital plane). Thanks to the S/C movement a global surface image in term of released particles can be obtained.

Strofio measures the in-situ neutral particle composition at the lowest energy range (∼0 to a few eV), and the particle density in the exosphere.

MIPA will detect ions up to 15 keV with low mass resolution. It is specifically devoted to monitor the intense SW fluxes outside and inside the Mercury’s magnetosphere in the context of the planetary responses detected by ELENA.

PICAM is an ion spectrometer with good mass resolution. Its main objective is to detect and characterize low energy ions (up to 3 keV), so that a complete analysis of the ion and neutral composition in the Hermean environment will be obtained together with Strofio Science objectives

The System Control Unit (SCU) is located inside the ELENA box; it is devoted to the full electronics and S/W management of the SERENA units.

In the next Sect. 2, the main scientific objectives of the SERENA experiment are described in detail:

1. Chemical and elemental composition of the exosphere
2. Neutral gas density asymmetries
  - Latitude,
  - Day/night,
  - Dawn/dusk,
  - Altitude,
  - Asymmetries versus Solar Wind (SW)
3. Planetary ion composition
4. Planetary ions spatial and energy distribution
  - Global distributions,
  - Temporal variations versus SW
5. Plasma precipitation rate
  - SW precipitation
  - SW distribution in the inner magnetosphere
  - Magnetospheric ions (heavy ions)
6. Surface emission rate and release processes.
  - Localized surface emissivity induced by back-scattering
  - Time-averaged emissivity of surface features
  - Surface MIV
  - PSD
7. Particle loss rate from Mercury’s environment
  - Exospheric charge-exchange
  - Loss of planetary ions

The scientific requirements needed to meet the science objectives listed above are described in Sect. 3. The SERENA sensors basic concepts and performances are described in Sect. 4 together with the full SERENA System description. In Sect. 5, the ground calibrations of the SERENA sensors are shown. In Sect. 6, the operational profile of SERENA is described from technical and scientific point of view. Section 6 describes the instrument technical and scientific operations. In Sect. 7, the cruise configuration of the SERENA sensors and possible science objectives achievable before the orbit insertion at Mercury are described.

## SERENA Science Objectives

### Major Scientific Goals

SERENA is an instrument that comprises 4 sensors devoted to the detection of neutral and ionised particles in the Hermean environment.

**Fig. 1:**
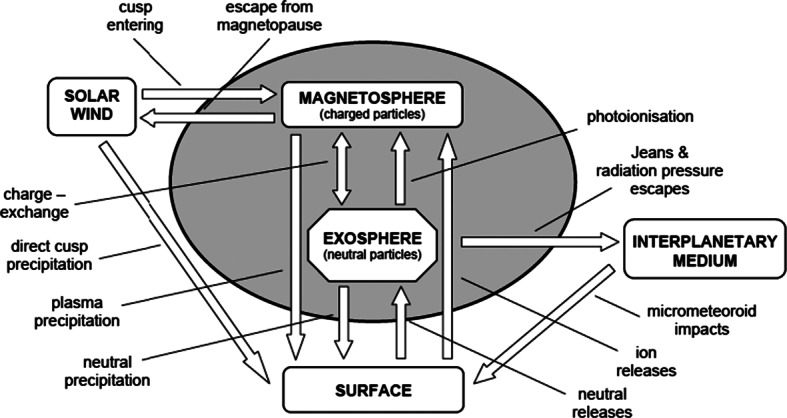
Schematic of the interacting processes (from Milillo et al. 2005)

The interaction between energetic plasma particles, solar radiation and micrometeorites with the Hermean surface gives rise to both thermal and energetic neutral particle populations in the near-planet space; such populations will be recorded by the SERENA Neutral Particle Analysers: a mass spectrometer and an energetic neutral atom imager. The photo-ionised or charged component of the surface release processes as well as the precipitating and circulating plasma in the Hermean magnetosphere will be recorded by the SERENA ion spectrometers: two ion sensors. In summary, BC/MPO/SERENA is an experiment capable to provide information on the whole surface-exosphere-magnetosphere system and the processes involved in the system as well as in the interaction with the SW and the interplanetary medium. A graphical summary of the acting processes is shown in Fig. 1. For a detailed description of the Hermean environment and the great improvement in its understanding expected from the BepiColombo mission (see Milillo et al. 2020).

SERENA deals with some of the main scientific objectives of the BepiColombo mission: composition, origin and dynamics of Mercury’s exosphere and polar deposits; and structure and dynamics of Mercury’s magnetosphere (Benkhoff et al. 2020).

SERENA will contribute to answering the following basic scientific questions:

- What is Mercury’s relation with its parent star as an end member of our Solar System?
- What is the evolutionary history of Mercury, as an extreme case in the Solar System and paradigm of the extrasolar planet?
- What is the role of the weak magnetic field in Mercury’s evolution?

To address these questions a detailed analysis of the following tasks is crucial for the knowledge of the environment and the evolution of Mercury:

1. exosphere composition, spatial distribution and dynamics
2. planetary ions characterization and dynamics
3. surface release processes.
4. atmosphere/magnetosphere exchange and transport processes
5. escape, balance between source and sink, geochemical cycles

Each SERENA sensor is able to operate and to achieve its specific scientific objectives independently. In addition, the opportunity to operate the SERENA units simultaneously greatly improves the success of addressing these scientific objectives and allows for additional objectives.

#### Chemical and Elemental Composition of the Exosphere

It is expected that the nine observed elements (H, He, O, Na, K, Ca, Mg, Al and Fe) may constitute only a fraction of Mercury’s exosphere (e.g., Milillo et al. 2005; Wurz and Lammer 2003; McClintock et al. 2008; Wurz et al. 2010). The quantification of different exospheric components is crucial for the determination of the environment composition since the neutral component is its primary constituent.

Of particular interest are the density measurements of Ca, Mg and other refractory materials, tracers of the ion sputtering and the impact vaporization processes, and of OH or water, tracers of hydrated minerals and water on the Hermean surface (Wurz et al. 2010). Many molecules are expected in the exosphere as a result of micrometeoroid impact vaporization (Berezhnoy and Klumov 2008; Berezhnoy 2018).

Strofio will obtain the global exospheric composition at MPO orbit. Strofio will also determine the aggregation status of atoms and molecules in the exosphere. Strofio measurements can be done on the dayside as well as on the shadowed regions and do not depend on specific emission or absorption lines.

#### Neutral Gas Density Asymmetries

The measurements of the spatial distributions of both neutrals and ions constitute a tool for understanding the ejection processes that caused their release as well as for getting information about the history of the particles during their trajectories (e.g., dissociation, acceleration, etc.).

**Fig. 2:**
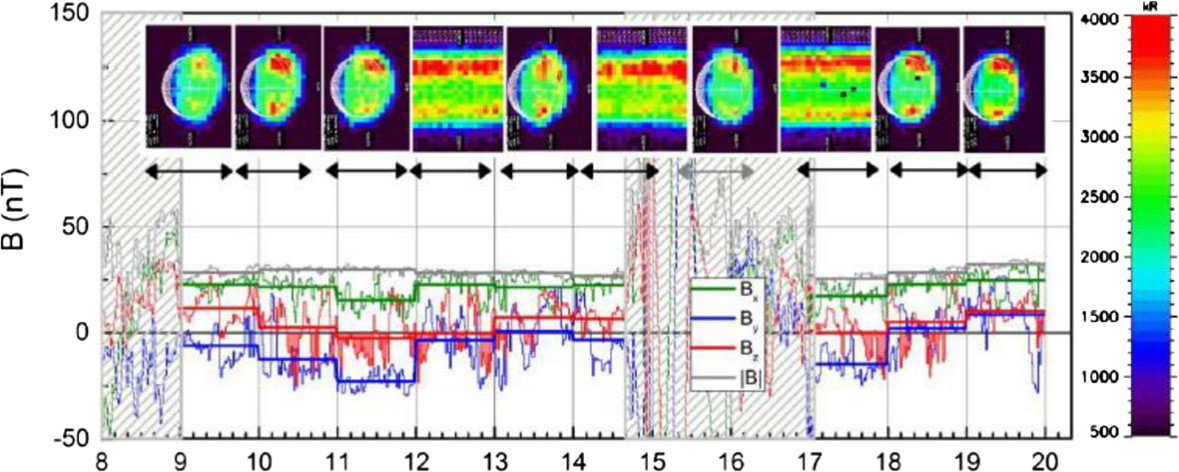
‘Standard’ (1-h long) and ‘fixed-slit’ (time resolution about 4 m) images, during 7 June 2012. The black arrows approximately indicate the acquisition time of each image. In the lower part of each panel, the plots of the IMF values measured by MESSENGER are shown (Bx, By, Bz and |B|, see legend). The 1-hour averages are superposed to the 1-min plots (same colors) of each IMF components. The dashed areas mask the periods when the spacecraft was inside the Mercurian magnetosphere and no in situ IMF data are available (Massetti et al. 2017)

**Fig. 3:**
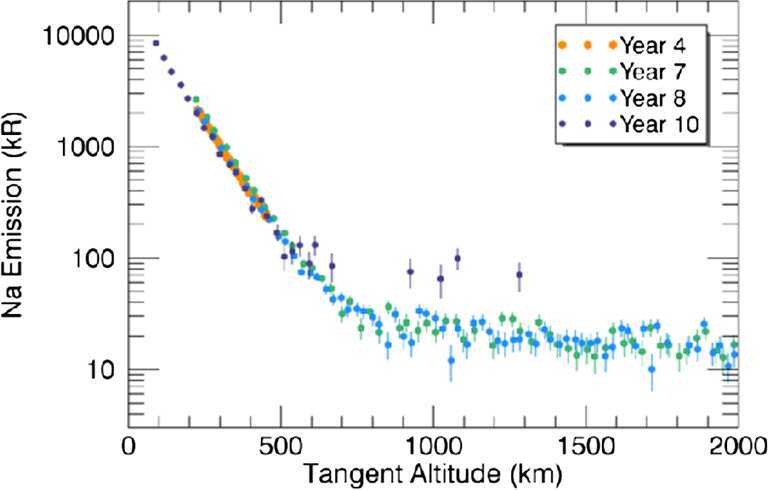
Na D_2_ emission intensity profile close to subsolar point observed at different Mercury’s year by MESSENGER/MASC (Cassidy et al. 2015)

Ground-based observations of Na and K distributions show high to mid-latitude enhancements, decreasing toward the terminator, which appear and disappear on timescales of hours (Potter et al. 1999; Leblanc and Doressoundiram 2008). Temporal fluctuations (time scale less than one hour) in the optical signal (Na D_2_ line emission) has been observed (Massetti et al. 2017) (Fig. 2). However, the mostly-equatorial measurements performed by the UV spectrometer MESSENGER/MASCS show a seasonal repeatability of the Na vertical profile (Cassidy et al. 2015) (Fig. 3). Strofio will provide the exospheric Na mapping in the dayside as well as the mapping of the close-to-planet tail on the night side. It will be a useful reference for observations of column densities through optical observations performed by MSASI on board Mio as well as from ground-based observations.

Moreover, asymmetries between different latitudes, day/night, dawn/dusk sides and perihelion/aphelion have been observed for different species, like Na, Ca and Mg in the Hermean exospheric density (Potter et al. 2006; Schleicher et al. 2004; Burger et al. 2014). Strofio will be able to observe these asymmetries. It will be of particular interest to analyse the density profiles for different species released from surface via different mechanisms and subjected to different processes. For example, Na and K, being volatile, are expected to have substantially different distributions as compared to the refractories, as Ca and Mg (Killen et al. 2005). In fact, this was observed by MESSENGER/ MASCS instrument (Killen et al. 2010). Strofio will record also the time variation in exospheric components for investigating the relationship with the external conditions.

#### Planetary Ions Composition

**Fig. 4:**
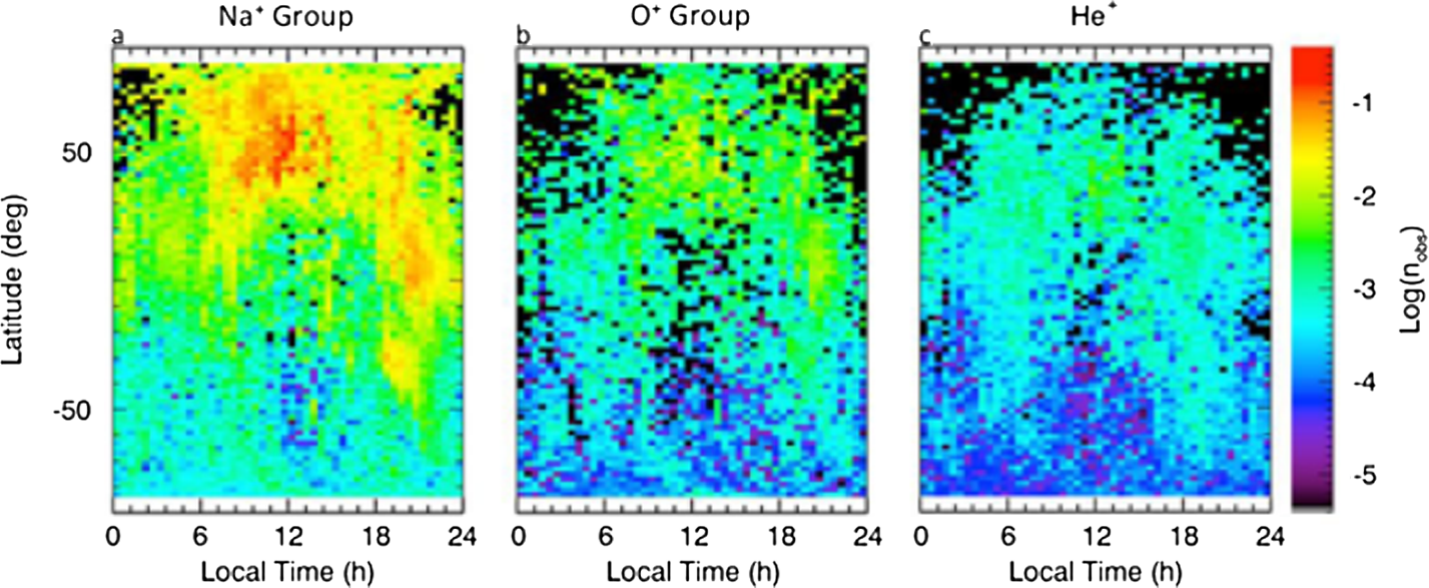
Na^+^-group (**a**), O^+^-group (**b**), and He^+^ (**c**) ion observed density along the MESSENGER orbit as a function of local time and planetary latitude (note that the northern data refer to lower altitudes). Observed regions with zero counts are *colored black* (Raines et al. 2015)

Ions of planetary origin (like the mass groups He^+^, O^+^+OH^+^, Na^+^+Mg^+^, Si^+^, S^+^, K^+^+Ca^+^) have been observed by MESSENGER/FIPS in the magnetosphere in the northern hemisphere (Zurbuchen et al. 2011; Raines et al. 2015) (Fig. 4). These ions are present, more abundantly, in the dayside hemisphere of Mercury, probably due to photo-ionisation and ion-sputtering processes. Ionisation of sputtered material on the dayside cups and ion convection to the nightside has been recently modelled and could explain the MESSENGER/FIPS observations (Wurz et al. 2019). PICAM continuous measurements of these ions will enable a detailed composition measurement. The low altitude orbit of MPO will allow PICAM to perform a full coverage of ion species since they are generated in the nearby regions.

PICAM will allow us to complete, together with the neutral component measured by Strofio, the composition analysis of the Hermean particle envelope.

#### Planetary Ions Spatial and Energy Distribution

Along the MPO orbit at low altitude, PICAM will be able to detect ions created in the nearby regions, hence, ions maintaining, at least partially, the information about their generation process and about the location of generation.

**Fig. 5:**
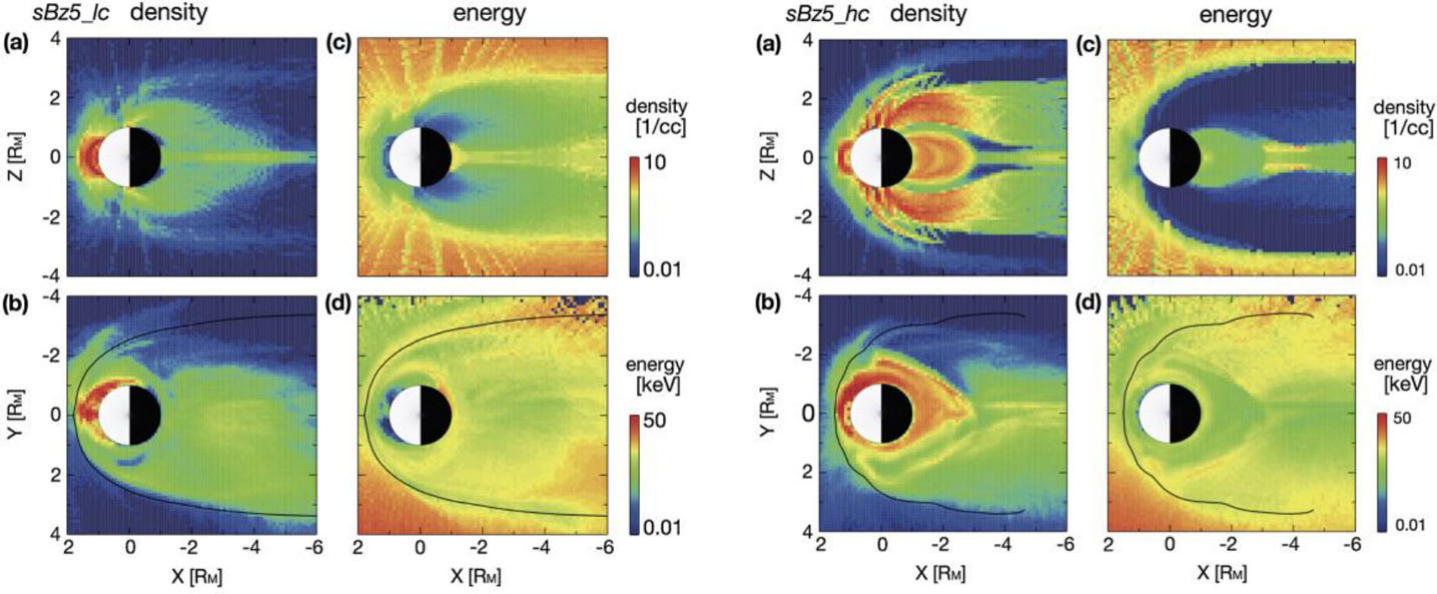
Na ion distribution under the same southward IMF (B_Z_ = −5 nT) and SW conditions, subject to different assumptions of surface conductance. *Upper panel*: low conductivity; *bottom panel*: high conductivity. The resulting ion distributions are markedly different as the formation of an X-line further from the planet inhibits escape in the second case (Seki et al. 2013)

The ions produced at thermal energies are energised and become part of the magnetospheric ion populations, together with the SW plasma entering through the cusp regions (see next Sect. 2.1.5). Model calculations of the Hermean ion environment (Delcourt et al. 2003; Seki et al. 2013) showed that the ions trapped at low altitudes in the magnetic field of Mercury are drifting with velocities determined by the configuration of the magnetic and electric fields and also different ion distributions are expected assuming different surface conductance (Fig. 5). PICAM will measure the energy distributions of ions in different regions along the MPO orbit. The high sensitivity of PICAM will permit detection of low flux of heavy ions. Simultaneous detection of ions originating from the planet by PICAM and ions originating from the Sokar Wind by MIPA will provide general information on plasma distributions in the close-to-planet magnetosphere.

Simultaneous Mio measurements of local plasma distribution inside the magnetosphere will permit reconstruction of the plasma distribution and dynamics on a more extended scale, leading to a deeper understanding of the governing processes (Milillo et al. 2020).

Coupling of the planetary ions with the exosphere and its dependence on external conditions can be determined by the measurement of the instantaneous and temporal variation measurement of the neutral (Strofio) to ion (PICAM) densities ratio around Mercury.

#### Plasma Precipitation Rate

The SW ions entering in the magnetosphere: (a) reach the planet’s surface at the cusp regions causing ion sputtering and ion neutralization and back-scattering, hence producing neutral atoms and ions with energies up to hundreds of eV; (b) partially are diffused toward closed field lines and circulate in the magnetosphere; (c) partially exchange their charge with the thermal exospheric atoms, producing a Hydrogen-ENA signal in the keV range. The intense flux of SW protons circulating at low altitudes, toward and from the planet will be measured by MIPA.

**Fig. 6:**
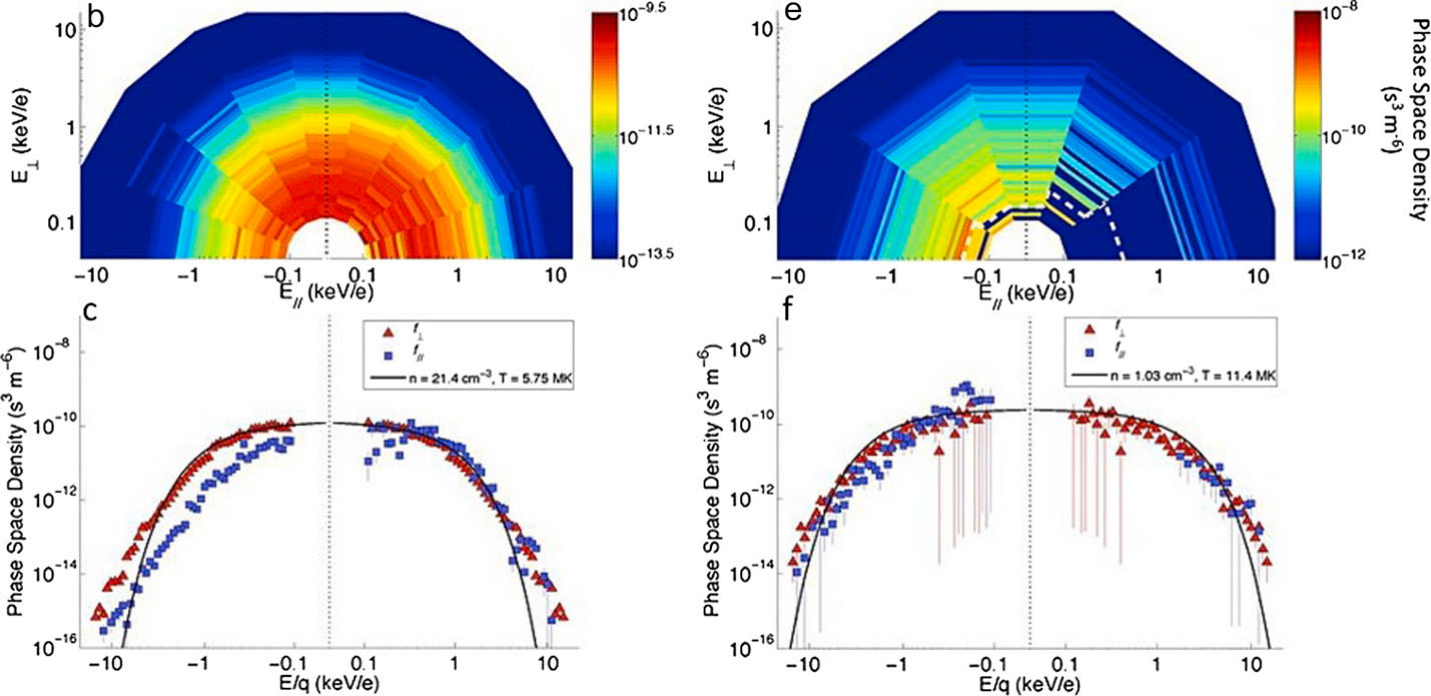
Kinetic properties of protons and Na^+^-group ions within the cusp. Top panels (**b**), (**e**) are energy-resolved pitch angle distributions, which show the flow direction and energy of ions relative to the magnetic field in 20^∘^ (protons) and 36^∘^ (Na^+^-group) bins. Slices through these distributions in the parallel, anti-parallel and perpendicular directions are shown in the bottom panels (**c**), (**f**). The Figs show protons that are flowing down toward the surface, as well as loss cone of > 40^∘^ in width. Low energy (100–300 eV) Na^+^-group ions appear to be upwelling from the surface, while those at energies up to 10 keV have large perpendicular energy components (Raines et al. 2014)

Clear signatures of plasma precipitation in the northern cusp is evident in both MESSENGER plasma and magnetic field data in the vast majority of orbits that cross this region (Winslow et al. 2012). In fact, Mercury’s dayside magnetopause is frequently experiencing reconnection as a result of the low Alfvénic Mach number ($\mathrm{M}_{\mathrm{A}} = \mathrm{V}_{\mathrm{SW}} / \mathrm{V}_{\mathrm{A}}$) conditions, where V_SW_ is the SW bulk velocity and V_A_ is the Alfvén speed and hence low-$\beta $ plasma (where $\beta $ is the ratio of plasma thermal to magnetic pressure) (DiBraccio et al. 2013; Slavin et al. 2018). The precipitating fluxes of heavy planetary ions are expected to be lower than the precipitating SW fluxes. For Na-group ions this flux has been measured by MESSENGER/FIPS (Raines et al. 2014) (Fig. 6). These planetary ions may hit the surface at mid latitudes (Kallio and Janhunen 2004; Massetti et al. 2003), causing an ion-sputtering process (Delcourt et al. 2003, 2007). Even if the estimation of these precipitating fluxes is difficult to obtain from model calculation, both MIPA and PICAM could give a hint in this challenging goal.

The magnetic field measurements from MESSENGER indicate that the Hermean magnetosphere is not symmetric, the internal magnetic dipole is not centred but offset northward by about 400 km (Anderson et al. 2011). Thus, the southern pole could be more exposed to SW precipitation. The southern hemisphere is less known, since the MESSENGER orbit was highly eccentric with its pericenter close to the North pole.

Generally, the high reconnection rate would lead to plasma precipitation even at lower latitudes. In fact, plasma precipitation usually occurs within open field line regions, like the Flux Transfer Events (FTE) (Slavin et al. 2012), depending on reconnection rates, resulting in a broad area of intense plasma precipitation on the surface, especially in case of SW disturbances. Occasionally, precipitation onto the surface is not only limited within open field lines regions. Plasma impact onto most of the dayside surface could occur especially in extreme conditions, like interplanetary coronal mass ejection (ICME), as predicted by models (e.g., Kallio and Janhunen 2003) and inferred by MESSENGER observations (Slavin et al. 2014, 2019; Zhong et al. 2015; Winslow et al. 2017). During high-speed streams (HSS) the induction effects produce a temporarily increase of Mercury’s magnetic moment (Jia et al. 2015, 2019; Dong et al. 2019), the shielding is less efficient during ICME events of slow and dense plasma (Slavin et al. 2014, 2018).

Thanks to its low-altitude orbit, MPO is the only platform useful for measuring and characterising the amount of SW ions (Massetti et al. 2003) as well as the heavier ions of planetary origin (Delcourt et al. 2003) that actually enter in the loss cone and, eventually, hit the planetary surface.

In summary, MIPA is devoted to measure the flux of precipitating ions at Mercury, which through ion-sputtering will be a source of neutral and ion emission from the planetary surface. MIPA is optimised to cover very wide dynamical range that allows to measure both the SW and heavy ion fluxes. The back-scattered H measurement performed by ELENA will directly provide an image of the surface area where the SW precipitates, to be compared to the measurement obtained by MIPA. ELENA, with ion deflectors switched off, will add an energy-integrated measurement in the zenith direction that the MIPA and PICAM field of view (FOV) doesn’t cover, adding a high angular resolution sampling. The identification of the composition and energy distribution of the planetary ion flux impacting the surface can be achieved by joint analysis of MIPA and PICAM with support of MAG magnetic field measurements.

#### Surface Emission Rate and Release Processes

A central problem for understanding the evolution of solar system bodies is the role played by SW, by solar radiation and by micro-meteorites bombardment in controlling mass losses through surface release (Killen and Ip 1999; Wurz and Lammer 2003; Orsini et al. 2014). The rate of surface ageing due to thermal desorption (TD), photon stimulated desorption (PSD), and space weathering caused by ion impact and micrometeoroid impact vaporisation (MIV) is particularly relevant at Mercury.

PSD released species dominate the dayside exosphere when we consider only the presently measured exospheric species. Mura et al. (2009) and Sarantos et al. (2008) suggested that the PSD efficiency of Na release is increased by the action of ion impact. Hence, a major PSD contribution in populating the exosphere is expected in the regions where ion-sputtering takes place. Orsini et al. (2018) showed that there is a strong connection between plasma precipitating regions and Na emission configuration. Nevertheless, the MESSENGER observations of the Na density profile where interpreted as PSD because of the inferred temperature (Cassidy et al. 2015). However, in a more recent calculation based on first principles this Na profile was interpreted as a combination of thermal release and micro-meteoritic impact (Gamborino et al. 2019). Moreover, Kameda et al. (2009) show that average Na density depends strongly on the interplanetary dust distribution, hence suggesting a major role of the MIV process especially on the night side. On the contrary, the Ca and Mg observed distributions fit with a release much more energetic, up to 50’000 K, due to ion sputtering (Burger et al. 2014). Probably, surface material is released also in molecular form, that could be dissociated in a second step (Killen 2016; Plainaki et al. 2017). All these studies and more show that the puzzle of the exospheric refilling is still far from being solved.

The ion impact on the surface may cause not only the release of particles from the surface (ion sputtering), but also the backscattering and neutralisation of a small fraction of the impacting ions (Lue et al. 2014). In fact, a solar wind proton undergoes multiple scattering inside the first monolayers of the surface, and, in the end, it can escape from the surface (Oura et al. 2003). During the multiple scattering a portion of energy is lost and charge-exchange with surface atoms is possible. The identification of such a population can provide a dynamic map of ion precipitation especially on the dayside. Moreover, ion precipitation could produce enhanced diffusion of volatile atoms on the surface, and hence enhance the PSD process efficiency (Mura et al. 2009).

Strofio will detect exospheric neutral particles released mainly by PSD in the dayside (for most species the TD scale height being much smaller than the MPO periherm - Wurz and Lammer 2003) and by MIV in the nightside (added to the PSD released particles circulating from dayside). Different efficiencies in releasing different species will help in the process identification. ELENA will detect particles released only at higher energies originating from ion-sputtering and back-scattering processes. Eventually, the detection of neutrals over the whole energy range of each process, temporal and spatial variations to be performed by Strofio and ELENA, will allow us to identify the process responsible of their generation.

Moreover, the correlation of the ELENA released (mainly back-scattered) particles flux and the MIPA plasma precipitation measurements will allow us to study the cause and effect of the ion-impact process. The repeated high spatial resolution measurements of the flow of the released neutrals above specific surface features, like bright craters or bright polar regions or extended hollows regions performed by ELENA will allow the determination of the feature neutral atom emissivity; hence, it will be possible to map the efficiency of the back-scattering process on the surface and provide a possible input for the source location of the particles detected by Strofio.

#### Particle Loss Rate from Mercury’s Environment

The loss of endogenic material in the exosphere can be due to gravitational escape of exospheric particles, or by the ionization due to photon impact or charge exchange with SW protons.

The high-energy neutral products of the release processes as well as the charge-exchange ENA, are created close to the surface and carried outward the planetary environment due to their high velocity that exceeds the escape velocity (v_esc_ = 4 km/s, i.e., 2 eV for Na and 4 eV for Ca). Directional neutral atom measurements are crucial for evaluating the mass loss from the Hermean environment. ELENA will detect the charge-exchange ENA escaping from the exosphere by looking at the limb of the planet.

The ions produced at thermal energies by photoionization or charge exchange are energised and become part of the magnetospheric ion populations, together with the SW plasma entering through the cusp regions. The magnetospheric plasma partially impacts on the surface (Ip 1997; Delcourt et al. 2003); hence, these particles are absorbed by the surface at specific latitudes (Delcourt et al. 2003; Leblanc et al. 2003) and are redistributed over the planetary surface (Killen et al. 2004). On the other hand, part of the magnetospheric plasma is eventually lost to the SW (Ip 1997; Delcourt et al. 2003). Actually, pick up ions have been observed by MESSENGER (Slavin et al. 2009; Jasinski et al. 2017). Ion measurements are important for the planetary global mass loss estimation and provide key information on the formation and on the erosion of Mercury’s neutral exosphere.

PICAM will perform observations with a good velocity and suitable spatial resolution, with wide instantaneous FOV, to identify the ions that are being lost from the planet. For this measurement the MAG magnetic field data are needed from which information about nearby ion trajectories will be derived.

Finally, some hints to the global loss rate can be evaluated by the measurements performed by ELENA and PICAM, thus providing crucial information for deriving the past and present evolution of the planet.

### Further Scientific Goals

SERENA will be able to contribute in scientific objectives mainly addressed by other instruments of the BepiColombo payload, such as remote sensing of the surface composition, magnetosphere structure and dynamics, as planetary response to SW variations. In the following some brief description of these goals is given.

#### Remote Sensing of the Surface Composition

Due to the direct link between the exosphere and the surface, by measuring neutrals and ions at relatively low altitudes SERENA will offer the possibility to get information on the upper surface composition. Nevertheless, some release processes are non-stoichiometric, which means that only selected species are involved.

Therefore, to infer surface composition, it is really important to know the release mechanism and the surface properties. Hence, taking into account the effectiveness of the process in ejecting material, we can gain information on the surface composition.

Strofio composition together with ELENA ion precipitation and particle release mapping, that provide information on the refractories releasing regions, will allow deriving roughly the element abundances of the upper surface. Detection of specific volatiles like S, OH, Si, over the polar regions will help understand the nature of the bright polar deposits (Neumann et al. 2013).

Furthermore, information on the composition of the bulk regolith can be derived from the total escape rates of atoms and ions by Strofio and PICAM. Planetary ions can be either produced directly by sputtering or by photo-ionisation of sputtered neutral atoms. Some minor constituents at the surface (e.g. Li, Al, Ca) may be released as ions with very high efficiency and therefore they can be hardly detected by a neutral gas mass spectrometer, they will be detected probably only by a high sensitivity ion spectrometer like PICAM.

In conclusion, this is not a major science objective of SERENA, but the comparison of this surface composition analysis with the most specifically devoted observations by MIXS and SIMBIO-SYS from MPO could be intriguing, too (Rothery et al. 2020).

#### Magnetosphere Structure and Dynamics

The solar wind ions have large gyro radii in the weak magnetic field of Mercury, thus these ions can be used as test particles that penetrate a significant portion of the magnetosphere. Ion measurements at the high-end of the energy range of the SERENA IS will provide information on the ions that have been drifted while penetrating the magnetosphere. Near the MPO apocentre, and when being close to the magnetopause on the dayside or on the flanks, an ion spectrometer (PICAM and MIPA) could observe ions subjected to the processes present in the SW-magnetosphere mixing layer, as reconnections and FTE or Kelvin-Helmotz instability observed predominantly at the dusk side of the magnetopause (Sundberg et al. 2012; Liljeblad et al. 2014). Furthermore, the SW ions entering into the planetary magnetosphere at the dayside (e.g. Kallio and Janhunen 2004; Massetti et al. 2003), as well as the protons circulating inside the magnetosphere (Mura et al. 2005) can interact with the exospheric atoms via charge-exchange, hence producing a Hydrogen-ENA signal in the energy range between several hundreds of eV and tens of keV.

When MPO will occasionally point off-nadir, towards the limb, as well as when considering the edge pixels of the field-of-view, which will observe the limb when the MPO spacecraft approaches the apoherm, ELENA will have the opportunity to observe the ENA, thus providing useful information on the plasma circulation close to the planet.

The combination of the data on convection in the Hermean magnetosphere provided by PICAM and MIPA associated to magnetic field information by MAG and together with correlated measurements onboard MMO, will help in understanding the structure and dynamics of the magnetosphere of Mercury. The high time resolution mode of MIPA is suitable to observe the fast magnetospheric dynamics. At close distances between MPO and Mio (lower than characteristic plasma scales), the capabilities of the instrumentation (fields of view, energy and mass resolution) complement each other. Coordinated measurements of both spacecraft inside Mercury’s magnetosphere but at large distance should be performed when the ion populations at the two positions are expected to show some relation, this may be the case if the positions are conjugate along a derived magnetic field line (Milillo et al. 2020).

Currents and plasma circulation at Mercury are controlled not only by SW conditions but also by possible currents system induced on the surface and mantle. Sixteen seconds at the MPO orbit at periherm correspond to one degree in latitude, but at the same time several seconds are the typical time scale of magnetospheric variations. Therefore, it is very important that two-point measurements at MPO and Mio are available to distinguish between spatial and temporal variations (Milillo et al. 2020).

By comparing the MPO/SERENA measurements to the Mio/MPPE observations in the SW, the response of the plasma environment of Mercury to the SW conditions can be studied. Simultaneity is crucial for the comprehension of the highly dynamic planetary response to SW and IMF variations (Milillo et al. 2005, 2020). The coordinated observations between the two BepiColombo s/c would allow for the first time simultaneous observations of the SW with Mio, and specific regions of the magnetosphere and exosphere along the MPO orbit:

- Dayside: analysis of the FTE occurrence and plasma precipitation toward the surface at MPO (SERENA IS) and, finally, a plasma monitoring at the surface obtained by the backscattered particles detection (ELENA), dayside exosphere short-term variation (Strofio);
- Lobes: analysis of the plasma circulation toward the tail, Dungey cycle, and possibly pick-up ions (SERENA IS), exosphere terminator distribution short-term variability (Strofio);
- Near tail: tail reconnection and FTE identification, ion convection at MPO (SERENA IS), night side precipitation onto the surface (ELENA), nightside exosphere short-term variability (Strofio).
- It is likely that the MPO spacecraft will be in the magnetosheath for some periods of the mission life time. Hence, in these configurations, MIPA will perform magnetosheath plasma measurements to be associated to the plasma and magnetic field signals observed from Mio inside the magnetosphere.

It is likely that the MPO spacecraft will be in the magnetosheath for some periods of the mission life time. Hence, in these configurations, MIPA will perform magnetosheath plasma measurements to be associated to the plasma and magnetic field signals observed from Mio inside the magnetosphere.

The SW flux at 0.3 AU is up to 10^8^ cm^−2^ s^−1^ sr^−1^. MIPA is more adapted to SW measurements by its larger energy range and larger dynamical range. A SW mode for PICAM is implemented, as well, for redundancy. MAG data are useful in this context to know when MPO is in different magnetospheric regions and to reconstruct particles trajectories.

## Scientific Requirements

For the accomplishment of the science objectives listed in Sect. 2, it is required that the following measurements are performed by the SERENA sensors when orbiting around Mercury during the nominal mission.

### Chemical and Elemental Composition of the Exosphere

The estimation of the exospheric densities can be derived from observations and models (see Milillo et al. 2005). The scale height for each species is derived by assuming a temperature $T$ = 500 K for volatiles and $T$ = 5000 K for refractory (Leblanc et al. 2004). The density at MPO orbit may be computed. The range has been evaluated by Leblanc et al. (2004) by taking into account the large uncertainty in estimating the real effectiveness of the release processes and the expansion in space.

The STROFIO observations will also benefit of the PHEBUS exospheric UV observations; in fact, such observations will be able to provide vertical density profile of the illuminated exosphere. A cross-calibration between these two instruments, at least for some selected species will be really useful for determining the 3D profile of the exosphere. Moreover, cross-calibration between STROFIO and of the MMO/MSASI spectrometer for Na density profiles will be done.

The estimated densities of the major species from sputtering or micrometeorite impact at 400 km are generally in the range 0.1 – 10 particles/cm^−3^ (Wurz et al. 2010). A mass resolution of M/$\Delta $M∼45 is needed to separate K from Ca at comparable abundance. Lower densities can be detected by Strofio by increasing the integration time. A mass resolution of M/$\Delta $M ∼ 60 will be necessary to detect also some minor species. The mass range of Strofio ranges from m/q=3 (He) up to m/q=56 (Fe), hence the H populations will be investigated with the PHEBUS instrument.

Such estimates refer to several studies on this subject. e.g.: Shemansky (1988): Mariner 10 measurements; Doressoundiram et al. (2009): observations; Leblanc et al. (2004); Killen et al. (2007): MSG measured abundance; Morgan and Killen (1997): model abundances; Killen (2002): model abundance; Burger et al. (2014): MSG measured abundance; Lakdawalla (2008); MSG measured ion abundance; Sprague et al. (1995): prediction.

In particular, the investigation of the radar-bright regions requires the mass discrimination of sulphur from water compounds, a mass resolution of M/$\Delta $M∼35 is sufficient for this goal. The oxygen aggregation status requires the discrimination of many oxygen bearing molecules as CaO, MgO and O_2_ achievable within the mass resolution requirement of M/$\Delta $M ∼ 60 and a sensitivity requirement of 10 particles/cm^−3^.

The on-board integration time for Strofio is programmable and can be adapted to the various phases of the orbit around Mercury. The natural guiding quantity is the time it takes the MPO to traverse a scale length in the exosphere: Monte Carlo simulations show that particles’ mean free path is equal to the altitude of the spacecraft, corresponding to a travel time of the order of 150 seconds at 400 km altitude, 750 seconds at apoherm. For the purpose of sizing telemetry we have baselined a constant integration time of 100 seconds. As the MPO orbit precesses slowly over the surface of Mercury, superposed epoch analysis will be used to enhance the signal to noise ratio for rare species.

### Neutral Gas Density Asymmetries

Strofio will be able to observe latitudinal asymmetries in a time range of half of a MPO orbit. Extended time-period measurements of Strofio are needed for deriving asymmetries between day/night, dawn/dusk sides and perihelion/aphelion. The altitude neutral gas density profile of the existing species in the altitude interval of the MPO periherm and apoherm (400–1500 km) can be derived by analysing measurements of Strofio more extended time periods and will complement the UVS measurements by PHEBUS that will characterise the density profile at lower altitudes in the dayside.

The range of densities of interest can be seen in Milillo et al. (2005), for example, Mg the estimated density at 1500 km altitude is still above 10 cm^−3^ (see also Wurz et al. 2010). Hence, the asymmetries mapping of trace-species as Na, Mg, S and Ca requires a sensitivity of 10 particles/cm^−3^ and a mass resolution of M/$\Delta $M∼35. The observation time must be at least half of an orbit for latitudinal characterization with a resolution lower than 10 min/mass spectrum and at least 1 full orbit for local time asymmetries investigation with a resolution lower than 30 min/mass spectrum. Note that the integration time on board will be always less than 100 s, so when talking about time resolution, we refer to continuous measurement to be integrated on ground. Due to the slow precession of the orbital plane over the planet, multiple orbits (10–15) can be co-added to reach the desired Signal-to-Noise ratio.

The investigation of exospheric time variability as a function of external conditions requires measurements in similar positions but occurring during different Sun activities. Considering that MPO arrival will be in the Solar cycle ascending phase, an appropriate statistical dataset can be reasonably achieved by collecting some minutes of observations every 3 hours for a total integration time of at least 1200 minutes in similar positions (for instance, close to sub solar point, close to dawn or dusk terminator).

### Planetary Ions Composition

The ion measurements to be performed by PICAM at MPO orbit during the nominal mission will cover a nearly full 2$\pi $ field-of-view. The mass resolution shall allow PICAM to discriminate between major species within the planetary ions, still not resolved by MESSENGER/FIPS that was able to resolve only ion groups (Fig. 6). A mass resolution better than M/$\Delta $M ∼ 50 is desirable. The specific orbit of MPO, close to the surface at all latitudes and covering all geographic longitudes and local time (LT) during the mission, will permit to detect ions generated from both hemispheres and in all the Sun-Mercury configurations. High sensitivity requires long integration time and is therefore in contrast to high spatial resolution discussed in the following section and is considered as a separate requirement. However, although for this scientific objective no specific spatial or time resolution is required, since the planetary ions composition can be related to external conditions, as many measurements as possible will be performed to increase the statistical significance.

In Table 1, third column, some estimates of the ion densities at the minimum altitude of MPO are listed. The uncertainties, especially for the ion component, are big due to the surface binding energy, the regolith composition, the porosity of the surface material, the efficiency by which a species is lost from Mercury’s exosphere and the main release process for each species (e.g. Seki et al. 2015; Orsini et al. 2004).

**Table 1:** SERENA scientific performances

Scientific topic	Sensor	Signal intensity @ 400 km	Energy resolution	Major components Mass resolution	Angular/spatial coverage Angular resolution	Time resolution	Observable region	Useful associated observations (see Milillo et al. 2020)
*1. Chemical and elemental composition of the exosphere*	Strofio	≤10^5^ cm^−3^ min req. 10^1^ cm^−3^	<1 eV Not req.	H, He, Na, Ca, CaO, H_2_, Mg, MgO, Si, others … M/ΔM>60	- Not req.	Not req.	Whole planet	Exosphere remote sensing
*2a. Neutral gas density asymmetries* *Latitude*	Strofio	≤10^5^ cm^−3^ min req. 10^1^ cm^−3^	<1 eV Not req.	H, He, Na, Ca, CaO, H_2_, Mg, MgO, Si, others … M/ΔM>60	- Not req.	$\Delta \mbox{T}<10~\mbox{min}$	Whole planet	Exosphere remote sensing
*2b, c. Neutral gas density asymmetries Day/night Dawn/dusk*	Strofio	≤10^5^ cm^−3^ min req. 10^1^ cm^−3^	<1 eV Not req.	H, He, Na, Ca, CaO, H_2_, Mg, MgO, Si, others … M/ΔM>60	- Not req.	ΔT<half orbit	Whole planet	Exosphere remote sensing
*2d. Neutral gas density asymmetries* *Altitude*	Strofio	≤10^5^ cm^−3^ min req. 10^1^ cm^−3^	<1 eV Not req.	H, He, Na, Ca, CaO, H_2_, Mg, MgO, Si, others … M/ΔM>60	- Not req.	$\Delta \mbox{T}<10~\mbox{min}$	Whole planet	Altitude density profiles variations
*2e. Neutral gas density asymmetries* *Temporal variation versus SW*	Strofio (MIPA)	≤10^5^ cm^−3^ min req. 10^1^ cm^−3^	<1 eV Not req.	H, He, Na, Ca, CaO, H_2_, Mg, MgO, Si, others … M/ΔM>60	- Not req.	$\Delta \mbox{T}<10~\mbox{min}$	Whole planet	SW monitoring
*3. Planetary ions composition*	PICAM	≤10^2^ cm^−3^ min req. 1 cm^−3^	>10 eV Not req.	H^+^, He^+^, Na^+^, Mg^+^, O^+^, K^+^, Ca^+^, others … M/ΔM>50	- Not req.	Not req.	Whole planet	
*4a. Planetary ions spatial and energy distribution*	PICAM	≤10^2^ cm^−3^ min req. 1 cm^−3^	>10 eV ΔE/E<30%	H^+^, He^+^, Na^+^, Mg^+^, O^+^, K^+^, Ca^+^, others … M/ΔM>40	2*π* in the orbit plane Δ*α*<25^∘^	$\Delta \mbox{T}<2~\mbox{min}$	Whole planet	Fluxes and fields from different v.p.
*4b. Planetary ions spatial and energy distribution temporal variation versus* *SW*	PICAM MIPA	≤10^2^ cm^−3^ min req. 1 cm^−3^	>10 eV ΔE/E<30%	H^+^, He^+^, Na^+^, Mg^+^, O^+^, K^+^, Ca^+^, others … M/ΔM>40	2*π* in the orbit plane Δ*α*<25^∘^	$\Delta \mbox{T}<2~\mbox{min}$	Whole planet	SW monitoring Fluxes and fields from different v.p.
*5a. Plasma precipitation rate* *SW*	MIPA (ELENA)	≤10^8^ $(\mbox{cm}^{2}\,\mbox{s}\,\mbox{sr})^{-1}$ min req. 10^6^ $(\mbox{cm}^{2}\,\mbox{s}\,\mbox{sr})^{-1}$	0.5–10 keV ΔE/E<30%	Mainly H^+^. 3 ion groups	2*π* in the orbit plane Δ*α*<25^∘^ polar × 60^∘^ azimuth	$\Delta \mbox{T}< 1~\mbox{min}$	Mainly dayside	Magnetic field Fluxes and fields from different v.p High energy particles and Xrays
*5b. Plasma precipitation rate* *SW distribution in the inner magnetosphere*	MIPA PICAM	≤10^8^ $(\mbox{cm}^{2}\,\mbox{s}\,\mbox{sr})^{-1}$ min req. 10^6^ $(\mbox{cm}^{2}\,\mbox{s}\,\mbox{sr})^{-1}$	0.5–10 keV ΔE/E<30%	Mainly H^+^. 3 ion groups	2*π* in the orbit plane Δ*α*<25^∘^	$\Delta \mbox{T}< 1~\mbox{min}$	Whole planet	Magnetic field Fluxes and fields from different v.p High energy particles and Xrays
*5c. Plasma precipitation rate* *Heavy ions*	MIPA PICAM	≤10^6^ $(\mbox{cm}^{2}\,\mbox{s}\,\mbox{sr})^{-1}$ min req. 10^5^ $(\mbox{cm}^{2}\,\mbox{s}\,\mbox{sr})^{-1}$	0.5–50 keV ΔE/E<30%	Mainly Na^+^, Ca^+^ M/ΔM>10	2*π* in the orbit plane Δ*α*<25^∘^ polar × 60^∘^ azimuth	$\Delta \mbox{T}< 1~\mbox{min}$	Whole planet/nightside	Magnetic field Fluxes and fields from different v.p. High energy particles and X-rays
*6a. Surface emission rate and release processes.* *SW – back-scattering emission*	ELENA	Up to 10^8^ $(\mbox{cm}^{2}\,\mbox{s}\,\mbox{sr})^{-1}$ min req. 10^6^ $(\mbox{cm}^{2}\,\mbox{s}\,\mbox{sr})^{-1}$	100 s–1000 s eV Not req.	H	Of the order of 1 R_M_ on the surface, i.e.: 5^∘^ × 60^∘^ (nadir centred) $\Delta\mbox{s} < 100~\mbox{km}$, i.e.: Δ*α*<15^∘^	$\Delta \mbox{T}< 1~\mbox{min}$	Mainly dayside middle-latitude	Magnetic field Fluxes and fields from different v.p.
	MIPA	≤10^8^ $(\mbox{cm}^{2}\,\mbox{s}\,\mbox{sr})^{-1}$ min req. 10^6^ $(\mbox{cm}^{2}\,\mbox{s}\,\mbox{sr})^{-1}$	0.5–10 keV ΔE/E<30%	Mainly H^+^. 3 ion groups	2*π* in the orbit plane Δ*α*<25^∘^ polar × 60^∘^ azimuth	$\Delta \mbox{T}< 1~\mbox{min}$	Mainly dayside middle-latitude	
*6b Surface emission rate and release processes.* *Time-averaged emissivity of surface features*	ELENA Strofio	Up to 10^8^ $(\mbox{cm}^{2}\,\mbox{s}\,\mbox{sr})^{-1}$ min req. 10^6^ $(\mbox{cm}^{2}\,\mbox{s}\,\mbox{sr})^{-1}$	100–1000 eV Not req.	Not req.	Of the order of 1 R_M_ on the surface, i.e.: 5^∘^ × 60^∘^ (nadir centred) $\Delta \mbox{s} < 50~\mbox{km}$ i.e.: Δ*α*<8^∘^	$\Delta \mbox{T} <3~\mbox{min}$	Above specific target features	Surface composition, mineralogy and structure
*6c. Surface emission rate and release processes.* *Surface MIV*	Strofio	≤10^5^ cm^−3^ min req. 10^1^ cm^−3^	<2 eV Not req.	Mg, Si, O, Na, K, Ca, others … M/ΔM>50	- Not req.	$\Delta \mbox{T}< 5~\mbox{min}$	Whole planet mainly nightside	Dust detection Refractories detection
*6d. Surface emission rate and release processes.* *PSD*	Strofio (MIPA and ELENA)	≤10^5^ cm^−3^ min req. 10^1^ cm^−3^	<1 eV Not req.	H, He, O, Na, K, others … M/ΔM>60	- Not req.	$\Delta \mbox{T} <5~\mbox{min}$	Dayside	Ion precipitation and back scattered particles
*7a. Particle loss rate from Mercury’s environment* *Exospheric charge-exchange*	ELENA	≤10^7^ $(\mbox{cm}^{2}\,\mbox{s}\,\mbox{sr})^{-1}$ min req. 5 10^5^ $(\mbox{cm}^{2}\,\mbox{s}\,\mbox{sr})^{-1}$	0.5–10 keV Δv/v<50%	mainly H Not req.	Up to hundreds km above the planet, i.e.: 5^∘^ × 20^∘^ (toward horizon) Δ*α*<8^∘^	$\Delta \mbox{T}< 1~\mbox{min}$	Mainly when MPO is close to apoherm looking to the sunward horizon	c-e ENA at wider FOV
*7b. Particle loss rate from Mercury’s environment* *Loss of planetary ions*	PICAM MIPA	≤10^7^ $(\mbox{cm}^{2}\,\mbox{s}\,\mbox{sr})^{-1}$ min req. 10^5^ $(\mbox{cm}^{2}\,\mbox{s}\,\mbox{sr})^{-1}$	0.5–10 keV ΔE/E<30%	Mainly Na^+^, K^+^ Ca^+^ M/ΔM>50	2*π* in the orbit plane Δ*α*<25^∘^	$\Delta \mbox{T}< 1~\mbox{min}$	Whole planet at higher altitudes, Mainly the tail	Magnetic field Fluxes and fields from different v.p.

### The Spatial and Energy Distribution of the Planetary Ions

In Fig. 6, the calculated ions spatial and energy distributions expected for Na^+^ are shown. In the close-to-planet regions energies between 10 eV and 10 keV are expected. From models of planetary ion populations (e.g. Leblanc et al. 2003) it is expected that their angular distributions are highly variable along the orbit of MPO and will cover a wide range of arrival directions. In fact, cause of the low internal magnetic field, Debye length has same order of magnitude as the planet size, so that inside the hermean magnetosphere particles are randomly distributed according to their individual motions.

To obtain the density and energy spectrum of the ions as a function of position, the field of view of the instrument should be as large as possible to be able to cover the entire velocity distribution. The time resolution requirement is coupled to spatial resolution through the orbit parameters. Thanks to its wide field-of-view PICAM will allow collecting a large part of the distribution of the ions. An angular resolution of about 25^∘^ cone angle and spacecraft motion will permit PICAM to resolve spatial structures. This time interval must be compared with the duration of the PICAM imaging sequence, typically 1 min (varying with the acquisition scheme). For a good composition characterisation of the ion distributions, the required mass resolution is moderate (M/$\Delta $M < 40), while the required energy resolution should be $\Delta $E/E < 30%.

**Fig. 7:**
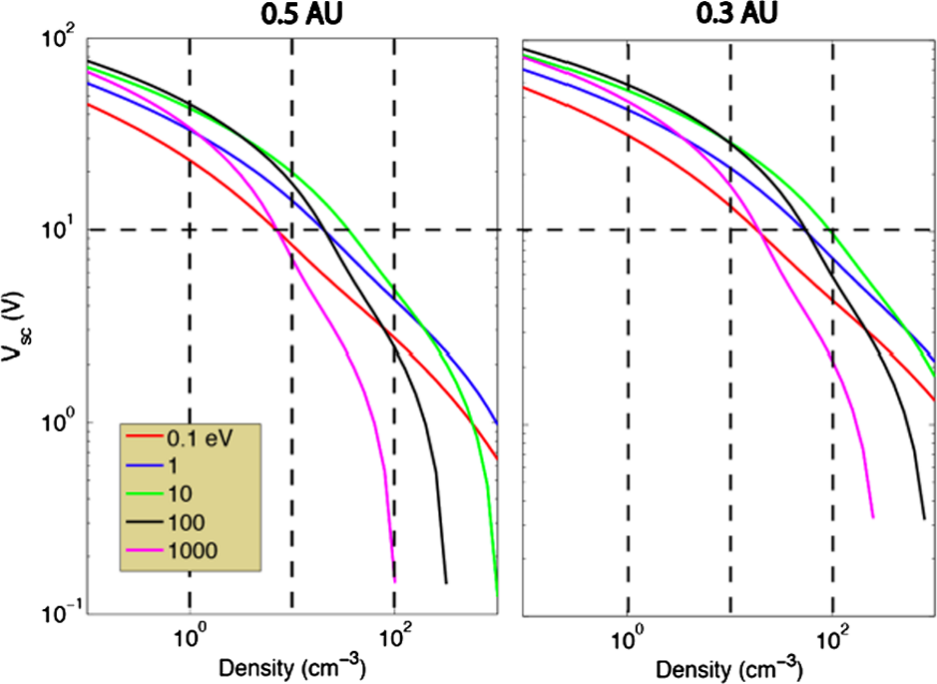
Spacecraft potential for 5 electron temperatures as a function of plasma density, computed at 0.5 AU and 0.3 AU, by assuming a spherical body. The satellite photoelectron population is described by three Maxwellian distributions (energies: 1.5, 7.5 and 15 eV, and the saturation currents are 5 nA cm^−2^ at 1 AU for 1.5 eV and 0.5 nA cm^−2^ for the other two)

Spacecraft potentials in the expected range of a few tens of volts positive will limit the capability to measure the lower end of the ion energy distributions. Figure 7 (H. Laakso, 2009, private communication) shows model calculations of the spacecraft potential as a function of the plasma density, under the assumption of a spherical body (based on orbit motion limit equations, courtesy of H. Laakso). The satellite photoelectrons are modelled by the superposition of three Maxwellian distributions (at characteristic energies of 1.5, 7.4 and 15 eV). The curves are computed at 0.3 and 0.5 AU, for five electron temperatures.

The spacecraft potential in different environment configurations and spacecraft attitudes will be evaluated to define the lower energy limit of the detectable ions. It will probably range between +10 V and +100 V. However, the spacecraft potential is correlated with the plasma density, and the lower the density the higher the spacecraft potential. Where the plasma density is extremely low (<10 cm^−3^), accurate composition measurements would be difficult in the first place. Spacecraft potentials will be moderate in high-density regions on the dayside, where photoelectrons are also significantly contributing at distances so close to the sun. In this context measurements in eclipse will be helpful to study this lowest energy range, which normally is hidden from the observations, and to calibrate the instrument response to spacecraft potential.

A good coverage of latitude and longitude with plasma measurements is needed to relate the planetary ion distribution to external conditions and to spatial structures of the magnetosphere and of the surface.

To reconstruct the ion trajectories, simultaneous MAG data at a time resolution at least comparable to, but preferably significantly higher than the sampling rate of PICAM (1 minute) are needed to identify at least roughly the pitch angle distribution. High time resolution of the magnetic field data also helps to find possible small-scale features in the ion distribution. The direction of the magnetic field at MPO is useful to derive flow patterns and to distinguish between precipitating and trapped particles. Simultaneous observations of MeV electrons and protons by SIXS will help to identify effects of variable solar input on the planetary ions. Simultaneous measurements of Mio in the SW will be very useful to relate the solar input with the planetary response in ion populations. To reconstruct the plasma distribution and dynamics on a more extended scale, simultaneous Mio 3D measurements of local plasma distribution inside the magnetosphere are needed (Milillo et al. 2020).

To relate planetary ion composition to external conditions as many measurements as possible are needed covering the full range of external conditions and to obtain sufficient statistical significance.

### Plasma Precipitation Rate

#### SW Ions Inside the Magnetosphere

The SW plasma enters the Hermean magnetosphere thanks to the high magnetic reconnection rate of the IMF with Mercury’s magnetic field, due to a general low $\beta $ condition in the magnetosheath. Preferential plasma entry regions are the cusps, where frequent FTEs have been observed (Slavin et al. 2012). Protons and heavy ions data collected in the cusps by MESSENGER/FIPS have been analysed (Fig. 6) (Raines et al. 2014). They show that the protons are flowing mainly down toward the surface, with a loss cone of > 40^∘^ in width. As mentioned in Sect. 2, the high-reconnection rate makes the plasma to precipitate not only through the cusps, but also at lower latitudes especially during disturbed conditions, when the cusps are extended towards the equator and occasionally the magnetosheath ions can impact directly onto the surface whenever the magnetopause approaches the planet (Orsini et al. 2018). Protons of SW origin migrate inside the inner magnetosphere toward dawn and/or pole-ward depending by the balance between electric and magnetic field effects, they are then energized toward the tail, in the plasma sheet, and then toward the planetary night side (e.g., Raines et al. 2015; Mura et al. 2005). This implies that proton fluxes detection should be performed by MIPA and PICAM in the whole dayside and also in night side, with a wide FOV. Required angular resolution is at least 20^∘^-30^∘^.

The expected time scale of these fields and particle fluxes variations is of the order of tens of seconds (Siscoe et al. 1975), and may be a few seconds for special SW conditions (Slavin et al. 2010). The intense flux of SW protons toward and around the planet will be monitored by MIPA when MPO will be in the low altitudes, but also PICAM can support the measurement especially in the night side where the flux intensity is lower. A specific high time resolution mode will be used for this analysis.

#### SW Precipitation

**Fig. 8:**
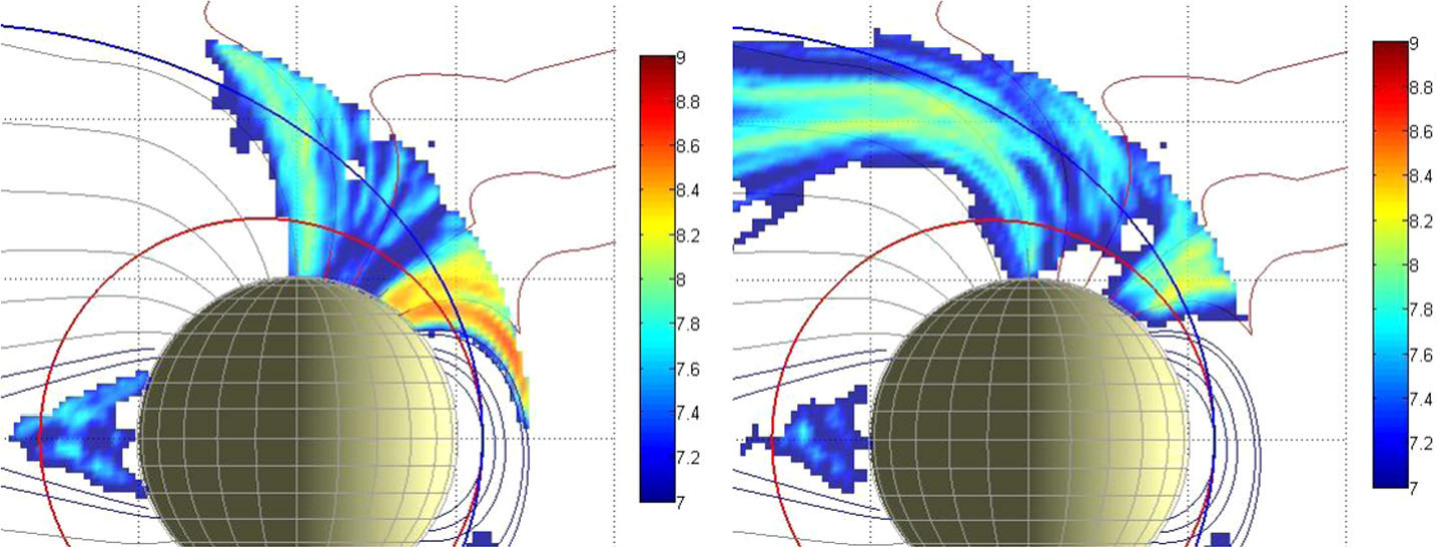
Colour-coded precipitating (pitch angle < 90^∘^, left panel) and the mirrored (pitch angle > 90^∘^, right panel) proton flux in case of IMF (−20, 0, −5) nT at aphelion. Sample orbit of the MPO spacecraft (red line) and Mio (blue line) with magnetic field-lines are traced in the xz plane (Massetti private communication)

**Fig. 9:**
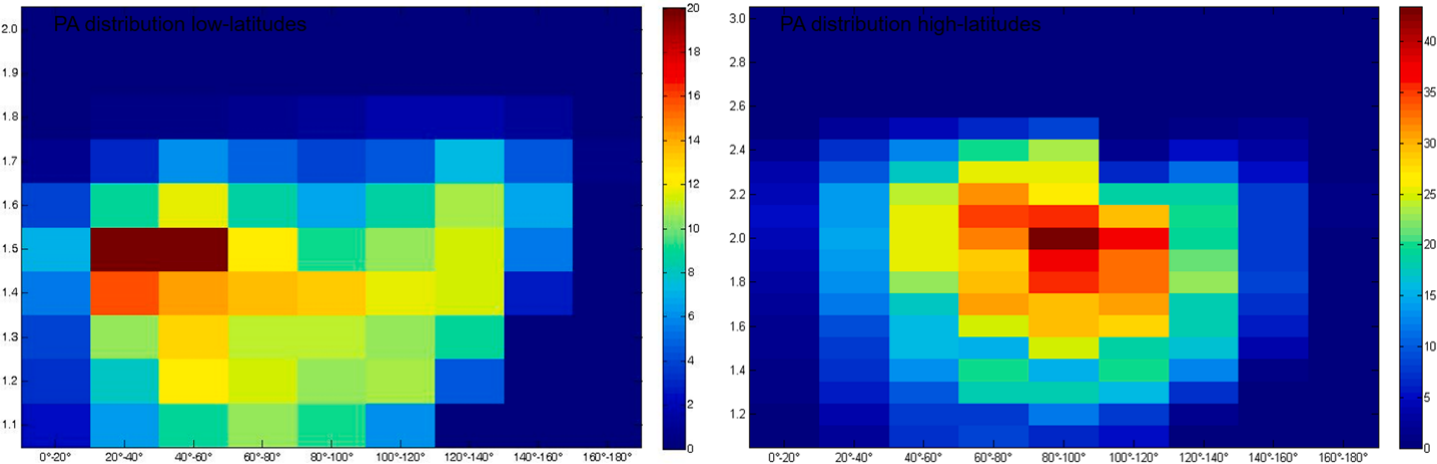
Pitch angle H^+^ distribution at low- (left) and high-latitudes (right), computed in case of IMF (−20, 0, −5) nT at aphelion (*Massetti* *private communications*)

In Fig. 8 an analytical-empirical model was used to estimate the downward (left)/upward (right) H^+^ flux that MPO (red line) and Mio (blue line) could find along their orbits, in case of IMF (−20, 0, −5) nT at aphelion. Figure 9 shows the H^+^ pitch-angle distribution at low- (left) and high latitudes (right), derived under the same assumptions of Fig. 8 and in accordance with the MESSENGER measurements shown in Fig. 6. Northward shift of the magnetic dipole should result in a wider open field line region in the Southern hemisphere. Of course, these simulations are an over-simplification of the real Hermean magnetosphere conditions. As mentioned in Sect. 2.1.5, the high-reconnection rate makes the plasma precipitation onto the surface not only at the cusps projection, but also at lower latitudes especially during disturbed conditions.

The science requirements for achieving this scientific objective are similar to the requirements for the general plasma circulation inside the magnetosphere, but with a specific attention to the downward (pitch angle close to < 90^∘^) and upward (pitch angle close to > 90^∘^) directed fluxes.

ELENA, operated with ion deflectors switched off, will be able to add a high angular resolution measurement of the mirrored ions in the radial direction, while ELENA in nominal mode will provide the map of the SW precipitation onto the surface by detecting the back-scattered particles.

**Fig. 10:**
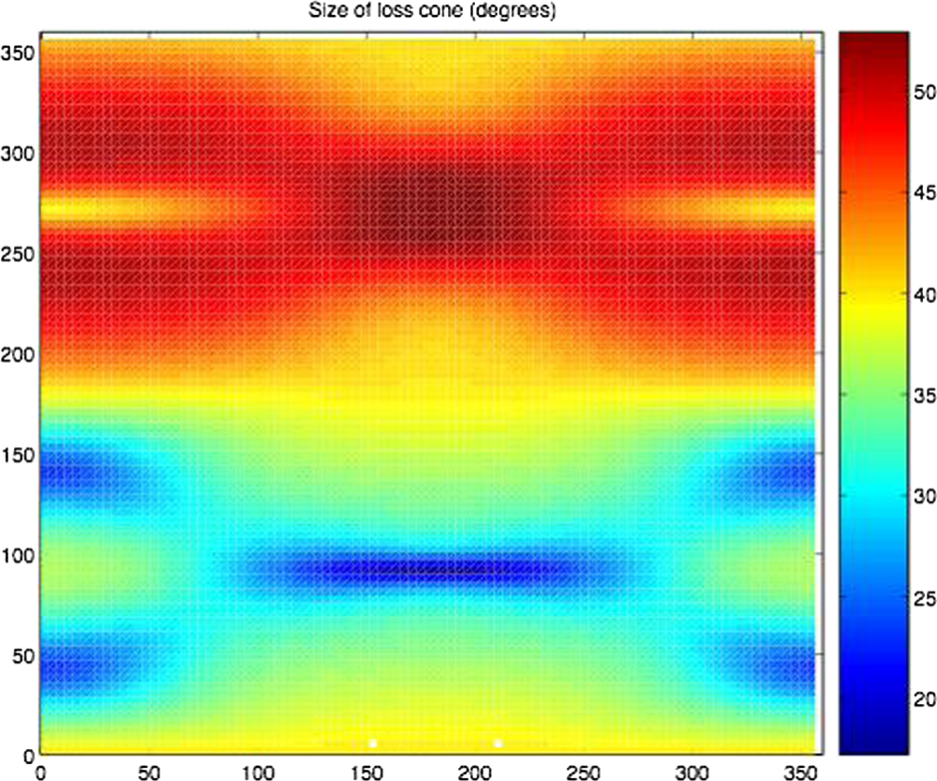
Estimated size of the loss-cone (degrees) along possible MPO orbits. The x-axis shows the longitude (0^∘^ towards the Sun) of the apogee. The $y$-axis shows the co-latitude of the true anomaly angle, apogee at 90^∘^ and perigee at 270^∘^

MIPA’s primary scientific objective is to measure precipitating ions; it only needs to detect ions within the loss-cone of about 40^∘^ wide. We have used a scaled Tsyganenko magnetospheric magnetic field model to estimate the size of the loss-cone shown in Fig. 10. The loss cone at the MPO altitude is almost always larger than 20^∘^, so that the required angular resolution is about 25^∘^.

If the shocked SW plasma entering Mercury’s magnetosphere is fairly isotropic the precipitating flux can be calculated based on low angular resolution ion measurements combined with magnetic field measurements. If, for example, the shocked SW is gyrotropic, but with a pronounced temperature anisotropy, it may be sufficient to resolve two down-going directions. If the temperature anisotropy is fairly constant it may be sufficient to estimate the total flux.

The binning of MIPA data must be flexible enough to allow for a trade-off between energy, time and angular resolution in the low telemetry modes. The choice must be made based on actual observations made in a high time resolution mode before. Suitable time resolutions must be < 1 minute.

#### Heavy Ions Precipitation

Seki et al. (2013) estimated the Na^+^ ion fluxes from the magnetosphere toward the surface on the night side up to 10^6^ cm^−2^ s^−1^ sr^−1^, with energies up to 10 keV (Fig. 5). Both MIPA and PICAM sensors have a wide FOV able to catch at least partially the heavy ion precipitating fluxes and will be operated to maximise the geometrical factors to detect this low ion flux and to focus on their highest energy range to detect at least part of the precipitating population.

In summary, MIPA will measure the flux of SW circulating and precipitating ions at Mercury. The identification of the composition and energy distribution of the planetary ion flux impacting the surface can be achieved by joint analysis of MIPA and PICAM. ELENA, operated with ion deflectors switched-off, will contribute to characterize the mirrored (or directed toward zenith) ions, while ELENA in nominal mode will provide the map of the precipitation onto the surface by detecting the back-scattered particles.

For this scientific investigation the simultaneous magnetic field data from MAG at a time resolution comparable or higher to the sampling rate of PICAM and MIPA are necessary to define ions pitch-angle distribution and precipitating ion flux.

During particular MPO-Mio configurations (when both spacecraft will be located along the same flux tube of precipitating ions, see for example the two orbits in Fig. 8 the ion fluxes could be observed from two vantage points (by Mio/MPPE sensors and MPO/SERENA IS), thus greatly improving the study of dynamical behaviour (Milillo et al. 2020). The frequency of such useful configurations will be evaluated when mission phases and detailed operations will be defined.

### Surface Emission Rate and Release Processes

**Fig. 11:**
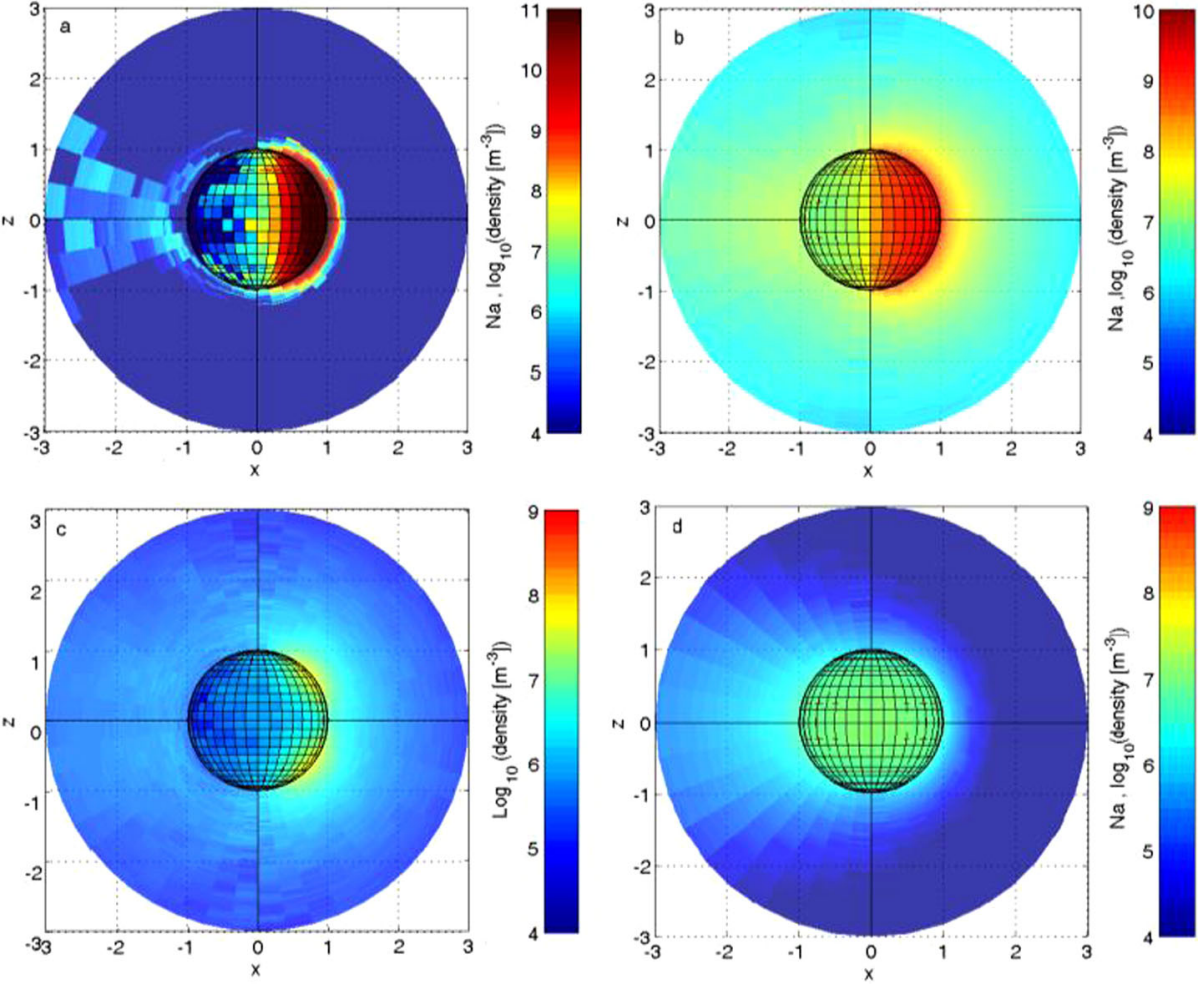
Exospheres modelled for different surface release processes. (**a**) TD; (**b**) PSD; (**c**) ion-sputtering; (**d**) MIV (Mura et al. 2007)

Observations of the atoms and molecules released from the planet’s surface as a function of latitude, longitude, local time, and external conditions, as solar irradiance or plasma precipitation are of crucial importance to identify and to localise the different physical processes acting onto the surface as well as to estimate their relative efficiencies. In Fig. 11, the surface release processes more active in the Hermean environment are modelled to evidence different roughly expected produced Na distributions to be compared to actual observations.

Volatile species originating by PSD will be measured by Strofio mainly in the dayside, but they can migrate to the night side producing also a tail induced by radiation pressure. Possible relation with plasma precipitation will evidence enhanced efficiency due to ion impact onto the surface. Strofio also discriminate the signal originating from the MIV, since it is probably the most important release process in the night side (Wurz and Lammer 2003) and since the refractories species and molecules are signature of this release process. In fact, meteoroid streaming along the Mercury orbit has been identified by Christou et al. (2015) associated to a seasonal increase of the Ca density. A mass resolution able to discriminate between volatile and refractory species, and some expected molecules like CaO and MgO (never detected at Mercury) is required (M/$\Delta $M>50). The characteristic energies of the emitted particles stay below few eV (Cintala 1992) even if the observed vertical density profile has been fitted with an exponential decay law at a virtual temperature reaching about 50’000 K, probably due to energization to some eVs after photon dissociation of the atom groups (Killen and Hahn 2015). Hence, at MPO orbit these particles are only observed by Strofio and not by ELENA.

Mangano et al. (2007) evaluated that during the BepiColombo mission the probability to observe a meteorite impact event is not negligible. For a meteoroid of 10 cm size the probability is close to 99% for a time range of a month. Such an impact will produce a strong increase in the refractory component of the exosphere (for example, for a meteoroid of 10 cm the Ca density will temporarily increase four orders of magnitude at MPO periherm). This signal will be detected by Strofio and it should not be associated with a simultaneous increase in the ELENA signal. Useful related simultaneous measurements of dust enhancement could be done by Mio/MDM, and it would be interesting to compare previous and subsequent high resolution images by SIMBIO-SYS to identify new craters (Milillo et al. 2020).

As stated above, the SW precipitation onto the surface produces two particle emission processes at the surface: back-scattering and ion-sputtering.

In the back-scattered process, the released particle is the same as the projectile. In the first approximation, it can be considered as multi-elastic hard-sphere collision between a high kinetic energy ion and an atom at the surface (Futaana et al. 2012). For an incoming mono-energetic ion flux of energy $E_{i}$, the back-scattering energy spectra shows, in general, a continuous profile between 0 and $E_{i}$. For heavier ions the scattered-ion energy $E$ is strongly reduced. We can consider that this process is relevant at Mercury mainly for the H^+^.

**Fig. 12:**
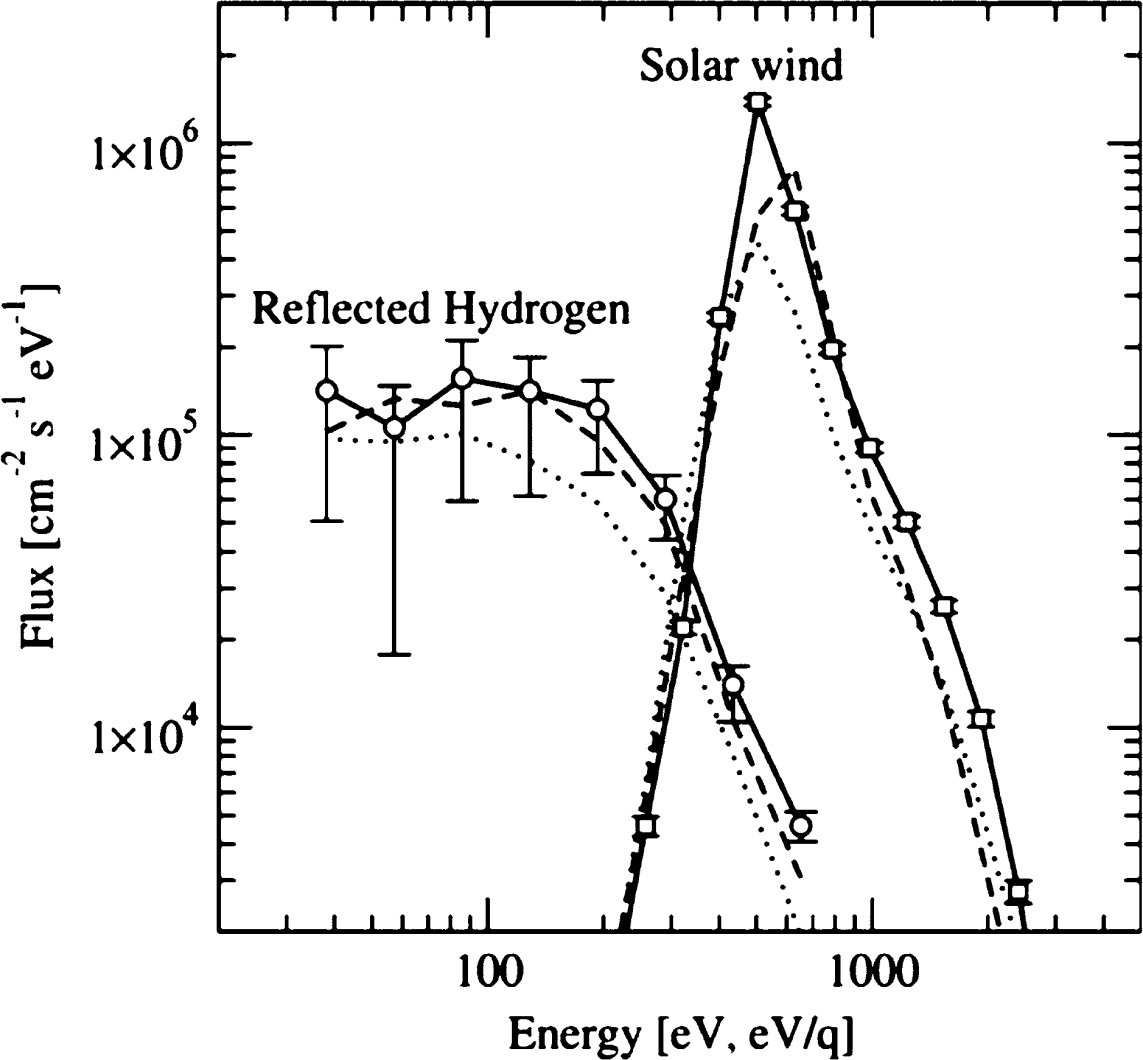
Chandrayaan-1 measurements taken shortly after the Moon crossed the Earth’s bow shock to the downstream direction. Energy spectra of the SW (right side, open squares) and of the corresponding reflected energetic hydrogen (left side, open circles) (Wieser et al. 2009)

The observations of neutral energetic hydrogen atoms from the Moon (McComas et al. 2009; Wieser et al. 2009; Vorburger et al. 2013) revealed that 10–20% of the SW is neutralised and back-scattered from the regolith surface, so that the estimated neutral back-scattered total flux at MPO orbit is about 10^7^ (cm^2^ s sr)^−1^. In Fig. 12 the energy distribution function of the back-scattered SW H from the Moon is shown (Wieser et al. 2009).

**Fig. 13:**
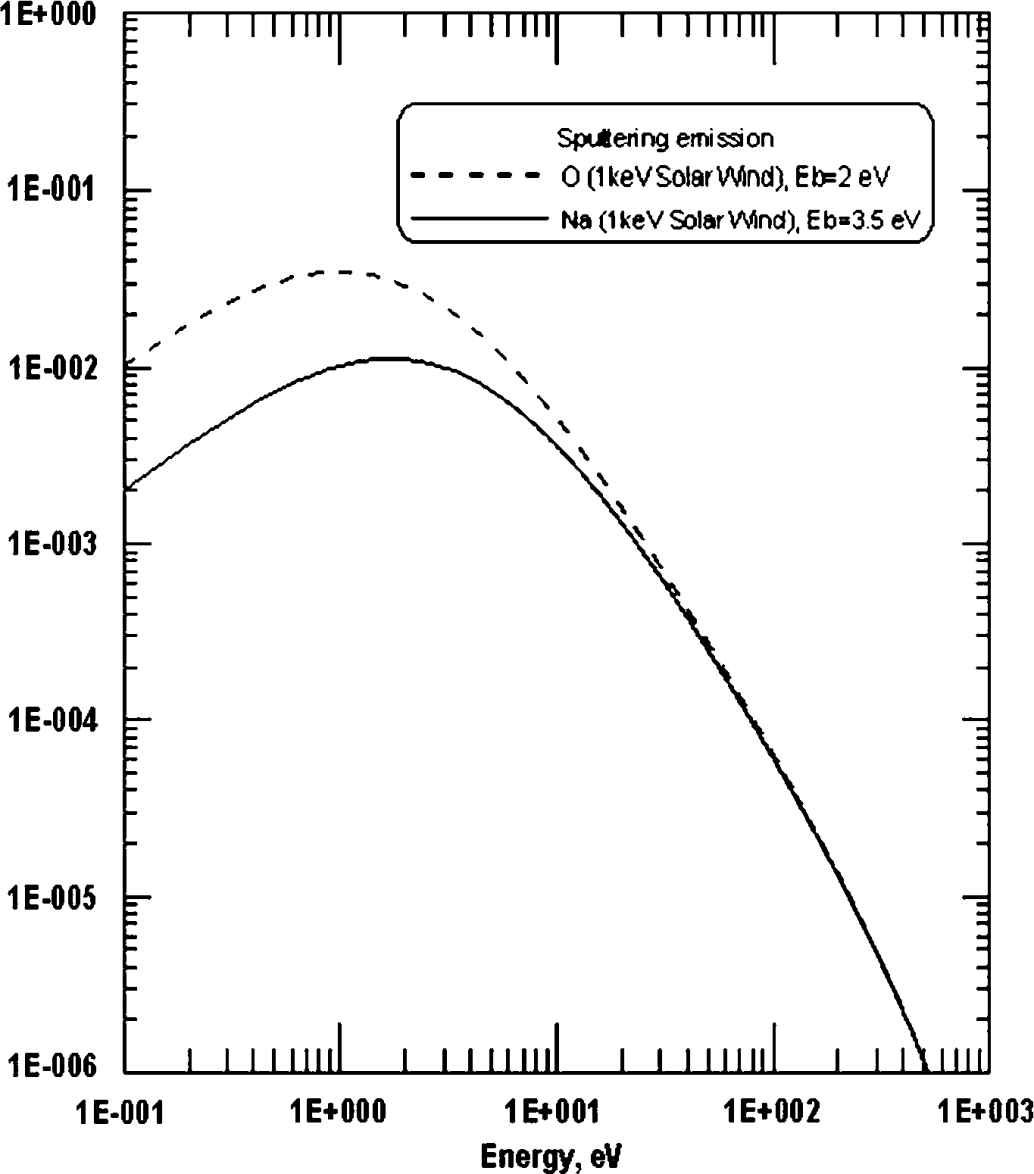
The energy distribution for sputtered particles, as a function of ejected Na and O particle energy in the case of 1 keV SW protons

The ion-sputtering process is a localised and highly variable release process. The intensity of the released flux depends by the plasma precipitating flux but also by the energy of impacting ions and by the element and the mineralogy of the target. Furthermore, differently from PSD and TD, ion-sputtering also involves refractory elements and has a wide energy spectrum with a significant high energy tail (e.g.: Milillo et al. 2011) (Fig. 13). The occurrence of ion sputtering can be identified by combining the ELENA and Strofio observations. The flux of the ion-sputtering neutral products of the release processes is estimated to be in the range 2⋅10^6^–2⋅10^7^ cm^−2^ s^−1^ sr^−1^ (Wurz and Lammer 2003). Strofio will provide detailed information about the composition of the exospheric particles, captured along the spacecraft ram direction, emitted from a wide region below the spacecraft. An ion-sputtering signal should be seen by Strofio as a non-recurrent variation in the refractory species of the exosphere. The comparison between this variation with ELENA signal increase, signature of plasma impact onto the surface, will provide information on this process. Considering the timing of flux variation and planetary response with respect to the particle release and transport toward MPO altitudes, the time resolution required for this measurement will be of the order of minutes.

**Fig. 14:**
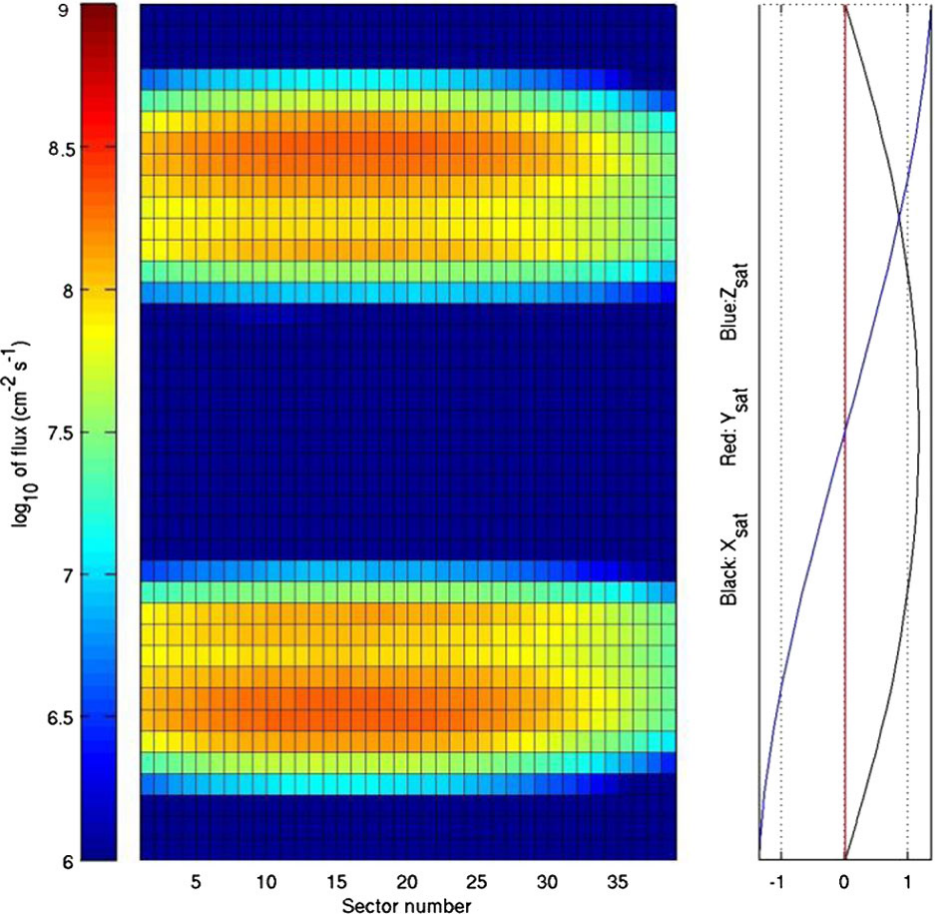
Estimated neutral back-scattered total flux impinging at the ELENA FOV sectors along the MPO day side orbit considering a yield of 10%

Given the existing link between precipitating particles and PSD particle release (Mura et al. 2009; Orsini et al. 2018), the comparison among the observations of neutral gas composition by Strofio, precipitating plasma by MIPA and instantaneous release from the surface by ELENA will permit to evaluate the PSD release efficiency with respect to ion precipitation and back-scattering release process in the observed region. Since the back-scattered signal has an energy spectrum peaking at higher energies where MCP efficiency is higher (Rispoli et al. 2013), the computed yield can be referred to the back-scattering process, only. The particle release detected by ELENA will allow the determination of the surface area from which the particles are escaping (that is roughly the region where the plasma impacts onto the surface). In Fig. 14, the back-scattered particles at the MPO orbit are simulated. The required spatial resolution is of the order of magnitude of the Larmor radius at the surface, hence tens of km for SW protons.

Simultaneous observations of magnetic field by MAG and of MeV electrons and protons by SIXS will help to identify periods of variable, i.e., increased solar activity.

Higher spatial resolution (about 50 km, i.e., about 7^∘^ at MPO periherm) is useful for investigating the surface composition inhomogeneities; in this case, time resolution is not required and many observations will be normalized (for precipitating flux) and averaged together.

This measurement will be strongly supported by other MPO instruments devoted to surface characterization such as: MIXS that will provide elemental composition, MERTIS that will provide information about mineralogy and SIMBIO-SYS that will map the geological features (Milillo et al. 2020).

### Particle Loss Rate from Mercury’s Environment

**Fig. 15:**
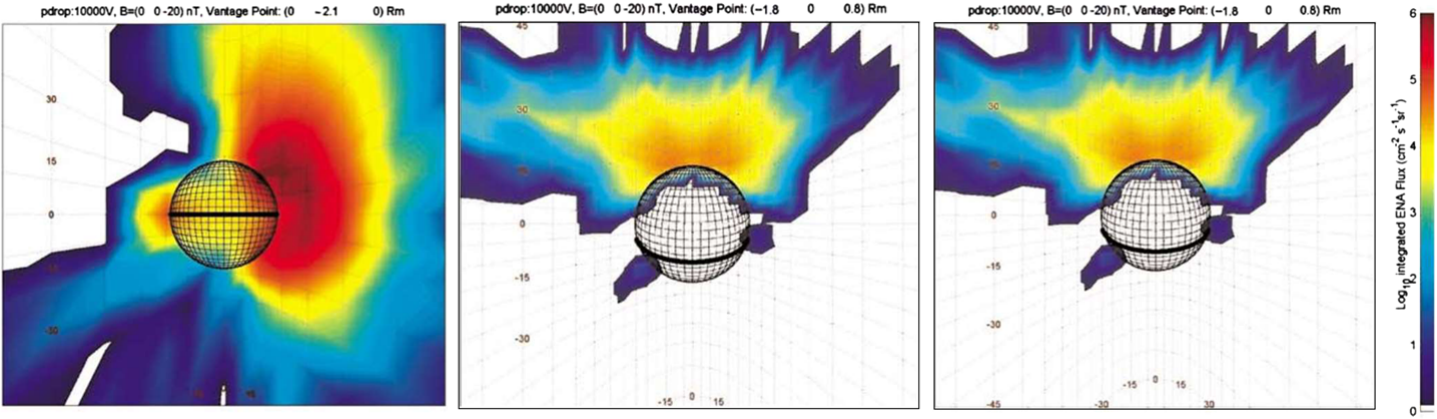
Simulated ENA images, from a vantage point in the nightside (P1 = (1.8, 0, 0.8)RM) (left and middle panels) and at dawn sector (P2 = (0, 2.1, 0)RM) (right panel). Color is coded according to log (ENA flux), integrated over energy ranges: 100 eV–1 keV (left panel) and 1–10 keV middle panel, and 100 eV -10 keV right panel. The boundary conditions are: BIMF = (0, 0, −20) nT; PD = 10 kV (Mura et al. 2005)

Mura et al. (2005) have simulated the ENA signal due to SW protons entering through the cusp region. The charge-exchange ENA fluxes at Mercury are estimated in the range of 10^5^–10^6^ cm^−2^ s^−1^ sr^−1^ mainly coming from the morning/dawn side of the planet toward night/dusk side (Fig. 15). This signal is more intense when the line of sight is directed toward the tangential view of the planet, since the integrated column is longer and because the bulk of the neutral atmosphere is close to the planet. ELENA will observe the limb with the edge pixels of its field-of-view (between +30^∘^ and +45^∘^ from the nadir direction), when the MPO approaches the apoherm, or when the MPO will point off-nadir towards the limb of Mercury. Coordinated observations with ENA observed from farer vantage points from Mio/MPPE will improve this investigation by adding wider FOV to ELENA higher angular resolution measurements. The time scale of emission of such signals is the same as for the magnetospheric variations (i.e., ≈ 1 min), hence this ENA signal could be seen as localised bursts.

Ion flux measurements are important for the planetary global mass loss estimation. It is essential to determine the 3D velocity distribution and/or the mass spectrum of ions over a full 4$\pi $ field of view should be required. Nevertheless, if the ion distribution can be considered symmetric with respect to the magnetic field direction, the 2$\pi $ pitch angle distribution can provide the required information. The energy range shall start at thermal energies and go above 10 keV (Fig. 5, see also Seki et al. 2013). The mass resolution shall allow us to discriminate between major species in the planetary ions up to ∼50 AMU. The time resolution requirement is coupled to spatial resolution through the orbital parameters. PICAM shall be capable to resolve spatial structures of the order of 5 degrees in geographic latitude at periherm, equivalent to ∼80 s at maximum velocity over ground. This distance is about half of the typical size of the observed local structures in the neutral Na exosphere. MIPA can also support this investigation in the case the escaping fluxes are intense enough for its geometrical factor. Simultaneous observations of magnetic field by MAG at same or higher time resolution (<30 s) are crucial for calculating the ions trajectories. Higher time resolution of the MAG data up to ∼1 s will provide additional insight into the magnetospheric environment. The detection of pick up ions at the magnetospheric boundary will be possible only when the MPO orbit will cross the magnetopause. The largest ion escape flux is expected in the magnetospheric tail; hence, joint investigation with Mio/MPPE will greatly improve the definition of particle trajectories.

### Summary of Scientific Performances of SERENA

#### General Overview

For the first time, the joint observations of he SERENA sensors will allow investigating the complex interactions between the charged particles and the planet, thus answering many open questions on the efficiency of the surface release processes at Mercury.

The main goals and expected new results of each SERENA sensor are summarised below.

- *ELENA*: neutral back-scattering emission; neutral particle loss rate from Mercury’s environment. Such measurements are novel and could not be observed by MESSENGER, due to the lack of similar instrumentation.
- *Strofio*: chemical and elemental composition of the exosphere; neutral gas density asymmetries; Temporal variation versus SW. Exospheric composition has been measured by the MESSENGER/MASCS UV spectrometer but such measurements are novel and not possibly observed by MESSENGER, due to the lack of similar instrumentation
- *PICAM*: planetary ion composition; planetary ion spatial and energy distribution close to the planet; planetary ion spatial and energy distribution temporal variation versus SW. Such measurements performed at low altitude, at all latitudes and longitudes are mostly novel, in fact, MESSENGER orbit did not allow the observation of the southern hemisphere at low altitudes, furthermore the FIPS ion spectrometer had a smaller FoV that did not allow to observe the anti-sunward ions, the PICAM mass resolution is much better that the FIPS one that allowed to resolve only mass groups. For the first time it will be possible to observe the ion distribution of many new species.
- *MIPA*: SW ion precipitation and mirroring rate and distribution in the inner magnetosphere. Such measurements performed at low altitude, at all latitudes and longitudes are mostly novel, in fact, MESSENGER orbit did not allow the observation of the southern hemisphere at low altitudes, furthermore the FIPS ion spectrometer had a smaller FoV that did not allow to observe the solar wind and the anti-sunward ions.

#### Detailed Description

The SERENA scientific performances details are summarised in Table 1.

## Instrument Description

### ELENA

The ELENA unit will resolve intensity and direction of the incoming particle flux, which is escaping from the planet.

**Fig. 16:**
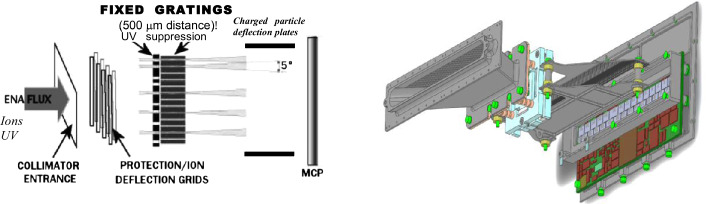
ELENA sensor concept

The ELENA sensor concept is showed in Fig. 16. The composite radiation made by neutral atoms, ions and photons impinges onto the ELENA sensor entrance. An ion deflector based on a grid system placed between the main ELENA entrance and the shutter suppresses the charged particle flux entering inside instrument. The transparency for particles through the grid deflectors is about 90%.

At the ELENA entrance there is a UV filter for photon noise suppression. It is composed by two Si3N4 membranes of 1 cm^2^, finely patterned with slits of the order of 200 nm wide and 1.4 μm pass (Mattioli et al. 2011). The membranes are located at about 500 μm distance to each other. This distance allows the neutral particles to enter inside the ELENA chamber independently on the membranes alignment, from any flow direction within the nominal FOV, while suppressing UV noise. The internal charge suppressor is a stack of particle cross-track plates which introduce a transversal E-field able to filter out the bulk of the charge particles of both signs.

Neutral particles are then flown in the ELENA box, and finally detected by a 1-dimensional array composed by MCPs and a discrete anodes set corresponding to a Field of View (FOV) of 4.5$^{\circ}{\times}76^{\circ}$, allowing the reconstruction of the direction of the incoming events. The spacecraft footprint track will provide the second dimension.

In this way, imaging of the neutral emission from planet’s surface is guaranteed, allowing to detect the particle generated by the major escape emission processes: back-scattering at hundreds eV and ion-sputtering mostly below 100 eV.

According to the actual simulations, the first signal is dominant respect to the second one (being the detector efficiency higher at higher energies), so that the ion-sputtering contribution could be partially masked due to the velocity-integrated information acquired.

By switching-off the deflectors (Elena ion mode) the ions directed along zenith can be measured, adding some more details on the anti-nadir angular distribution of these particles observed by PICAM and MIPA.

#### Science Performance Analysis

##### Estimated Flux

The energetic neutral particles that are likely to be detected by ELENA come primarily from back-scattering and also from ion-sputtering process and from charge exchange (Mura et al. 2005). To estimate the neutral flux measured by the instrument, we assume that, during an intense SW activity: SW density 60 cm^−3^, velocity 400 km/s, hence a flux of 2.5 10^9^ cm^−2^ s^−1^ at the magnetopause, a total of 5 10^26^ protons s^−1^ impact onto the surface (Leblanc et al. 2003, and references therein), considering the particle collimation inside the cusps a factor 2. Only 10% of this flux reaches the surface (Massetti et al. 2003) F_ion_ = 5⋅10^8^ cm^−2^ s^−1^ on average, this means that locally the flux could be even higher. These protons impact on roughly 50% of the dayside surface (i.e., an area of $\pi \ R_{M}^{2}$, $R_{M}$ = Mercury’s radius) (Kallio and Janhunen 2003; Massetti et al. 2003), and they cause back-scattering and sputtering (as well as PSD through enhanced diffusion) of various surface components, with a yield ($Y$) that is, on average, about 0.1 neutral particles for each incoming proton (Lammer et al. 2003), even if it depends on the considered surface neutral species.

Protons can be reflected by the surface (backscattering) and neutralized during the reflection. The back-scattering yield considered is between 10 and 20% (McComas et al. 2009; Wieser et al. 2009). We can estimate a maximum back-scattered neutral hydrogen flux at the spacecraft of 6⋅10^7^ cm^−2^ s^−1^ sr^−1^.

Because of the low abundance of heavy ions in the SW their contribution to the sputtered flux is negligible in normal conditions and only alpha particles contribute to the sputtered signal (about 30% to the total sputter yield; Wurz et al. 2007). During CME events the alpha and heavy particle abundances in the SW can increase (Wurz et al. 2003), thus, in this case, they can contribute to the process in a similar amount of protons (Johnson and Baragiola 1991), while the back-scattering efficiency should be lower since the kinematic factor of the heavy particles is higher and the reflection efficiency is lower (Plainaki et al. 2010).

The energy distribution $f$($E$) of those sputtered neutrals peaks at few eV (Sigmund 1969); nonetheless, since the energy needed to reach MPO altitude is, on average, below 1 eV, it has been estimated that 90% of the sputtered flux arrives at the spacecraft orbit. In summary, a maximum neutral sputtered flux *F*=*F*$_{\textit{ion}}$*$Y$*2*.9 of the order of 2⋅10^7^ cm^−2^ s^−1^. The sputtered particles are emitted with a complex angular distribution; here we assume (as a first guess) that they are emitted towards the vertical direction with a spread of $\pm \pi $/2 sr, thus a maximum sputtered flux is about 4⋅10^7^ cm^−2^ s^−1^ sr^−1^. About 1% of these particles are sputtered high energy atoms SHEA (Milillo et al. 2011) detectable by ELENA.

Charge exchange neutrals have energies of the order of 1 keV, and they are expected to be primarily H-ENAs. The maximum estimated H-ENA flux is about 10$^{6}\sim $10^7^ cm^−2^ s^−1^ sr^−1^ (Mura et al. 2005). This signal may be detectable only at apoherm, when a small part of the ELENA FOV looks tangent to the planet (Mura et al. 2005).

##### Geometrical Factor

For reference, we define the $x $ axis perpendicular to the entrance. The $y $ axis is perpendicular to $x $ and along the shortest border of the STOP MCP; the $z$ axis is parallel to the longest border of the MCP (see Fig. 16). The entrance of the instrument has a total area $S$ of 1 cm^2^. At the entrance, two identical grids (membranes) are placed. Each hole is 260 nm wide (*d*, in the vertical direction). The path for the holes is 1.4 μm (in the $y$ direction). The grids areas are 1 cm^2^ but to ensure rigidity, some parts of the grids are solid (without holes). Furthermore, the open area is reduced by the ion deflectors before the entrance. Finally the grids transparency to particles is T_g_ = 0.1 per grid. So the ELENA theoretical geometrical factor is G_bu_ = T_g_^2^ *FOV * Area = 1⋅10^−3^ cm^2^ sr.

##### MCP Efficiency

**Fig. 17:**
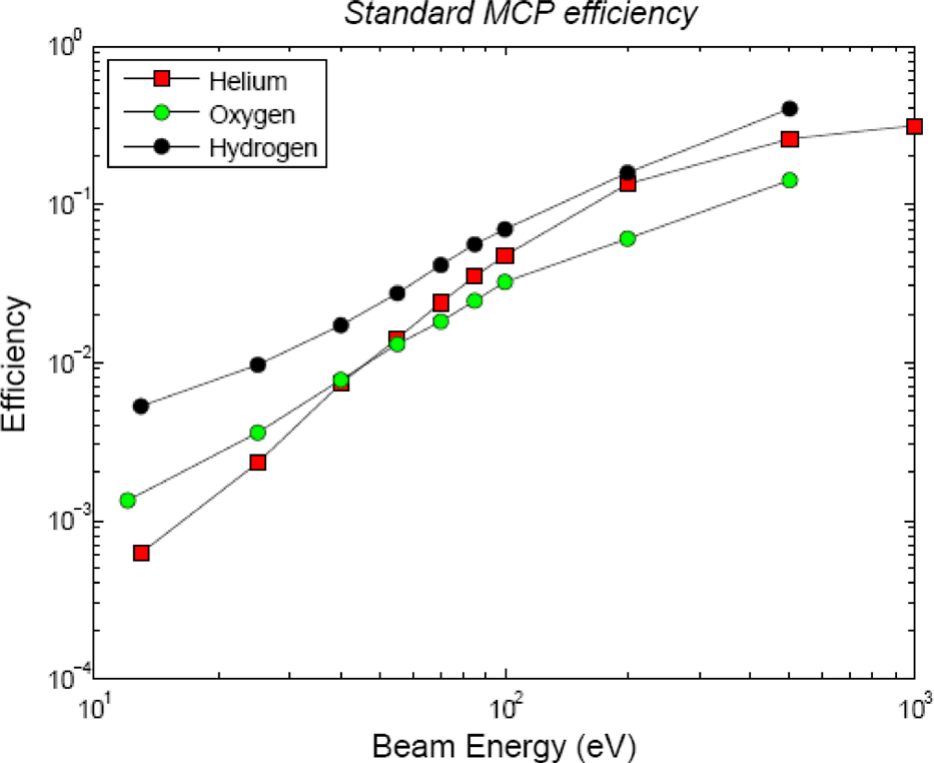
MCP efficiency to H, He and O impact as a function of energy resulting from test performed by the IAPS team at the MEFISTO facility at the Bern University (Rispoli et al. 2013)

The MCP detection efficiency for neutral particles in the range 10 eV – 1keV is a function of energy (Fig. 17, Rispoli et al. 2013). The MCP efficiency for protons at higher energies approaches unity.

##### Count Rates

The geometrical factor of ELENA is 1.0⋅10^−3^ cm^2^ sr. The three main expected signals are:

- proton backscattering expected flux is about 6⋅10^7^ cm^−2^ s^−1^ sr^−1^, the energy spectrum is between hundreds eV and 1 keV and hence the averaged MCP efficiency is about 20%. By using these numbers, we obtain more than 10^4^ counts per second.
- SHEA expected flux is about 4⋅10^5^ cm^−2^ s^−1^ sr^−1^. The MCP efficiency at energies <100 eV is about 1%, the expected signal is of few tens of counts per seconds so lower than the back scattered signal; since it comes from the same region from the surface, it is difficult to decouple the two signals.
- Charge exchange signal (about 10$^{6}\sim $10^7^ cm^−2^ s^−1^ sr^−1^) from the exosphere will be detected only in those angular sectors not pointing directly to the planet surface. Hence, in those sectors we expect only this signal. The MCP efficiency for 1-keV protons is almost 1; the geometrical factor must be reduced by a factor 10 because only 10% of the FOV is looking at the exosphere (and only at apoherm). The expected count rate is approximately 100 counts/s.

During the integration time (of the order of tens of seconds), the spacecraft is moving along its orbit and the footprint of ELENA FOV on the surface is moving as well. However, if the integration time is up to 60 s, the movement is small compared to the size of the footprint.

##### Pointing Requirements

**Fig. 18:**
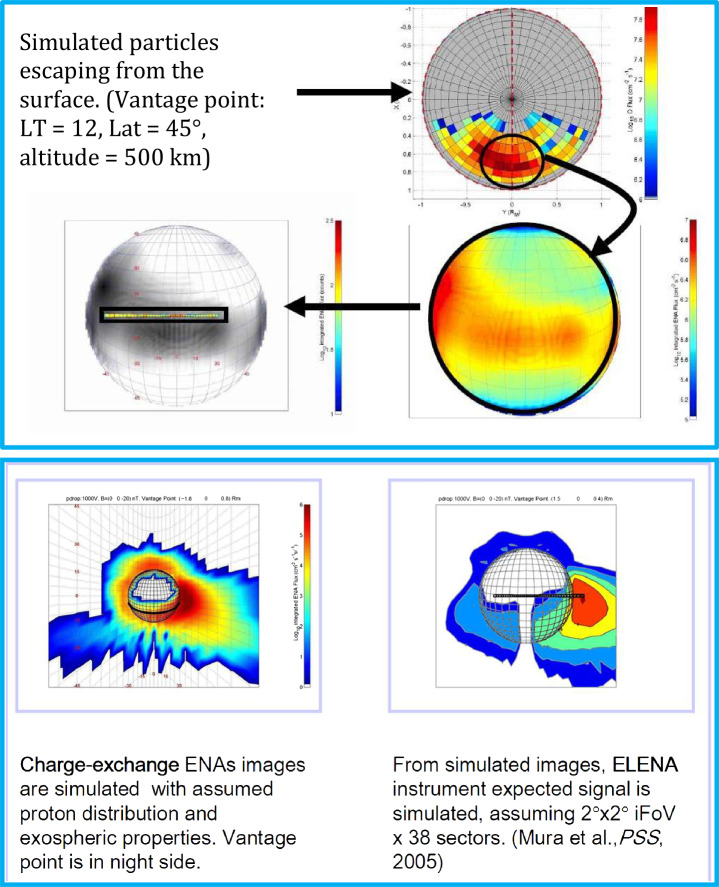
*Upper*: Simulation of the energy-integrated (between 20-1000 eV) signal from vantage point MLT=1200, 45^∘^ elevation and 500 km altitude is shown in the upper panel. The horizon at in this position is zoomed in the bottom-right panel and the slice of ELENA FOV is evidenced in the bottom-left panel. *Bottom*: Energy-integrated H ENA from the night side apoherm (left panel). The instantaneous FOV of the linear array of the ELENA sensor is shown as a slice in the right panel (from Mura et al. 2005, 2006)

The FOV of ELENA will cover a 4^∘^-latitudinal slice over a longitude range of 76^∘^, thus imaging a big portion of the surface (see Fig. 18, upper panel). The spacecraft footprint track will provide the second dimension for a 2D mapping of the surface.

In order to observe the planetary horizon at least when MPO is at apoherm the instantaneous FOV of ELENA must extend 46^∘^ longitudinally (i.e., perpendicularly to MPO orbital plane) from the nadir direction toward the Sun, hence opposite to spacecraft radiator. The charge-exchange ENA flux is only partially detected by ELENA mainly at apoherm (see Fig. 18, bottom panel), but with the help of models and of the observation from Mio/MPPE, the contribution to global loss could be evaluated as well. The MPO off-nadir pointing towards the limb of Mercury will add a possibility to observe a more extended area of charge-exchange ENA generation.

##### Background Noise

Possible background arising from Sun light, SEP and cosmic rays can affect the signal. A rough estimation of the background due to high-energy ions (>10 MeV) from galactic cosmic rays (GCR) and worst-case solar particle events (SPEs) at 0.3 AU is between 1 particle/cm^2^/s/sr and 10^4^ particles/cm^2^/s/sr, respectively (Mewaldt et al. 2001). ELENA MCP is inside the s/c and well protected, anyway in a worst-case estimation with a total MCP area of about 30 cm^2^, GCR will produce < 30 Counts/s. During a SEP event the background could be above the signal nevertheless, we expect few SEP events per year lasting a few hours.

The Ly-$\alpha $ (∼UV band 100-150 nm) flux at 0.3 AU is 4 10^12^ Ly$\alpha $/(cm^2^ s) (Schmidt 2013).

The albedo of the planet is ∼0.1 in the visible range (we apply the same overestimated factor for UV light).

The Ly$\alpha $ signal in the dayside is on average (open/closed cycle; average latitude):

$$ S_{\mathit{Ly}\alpha } =F{\cdot }0.1/ ( \sqrt{2} \pi ) {\cdot }\Omega {\cdot } T_{\mathit{UV}} {\cdot }\varepsilon \left ( \mathit{Ly}\alpha \right ) =5000\mathit{Ly}\alpha /s $$

Where $T_{\mathit{UV}} = 310^{-5}$ is the theoretical two grating transmission factor for 200 nm of the hole, $\Omega $ is the ELENA FOV=0.09 sr and $\varepsilon $(Ly$\alpha $) = 3 10^−2^ is the MCP efficiency to Ly-$\alpha $. In the night side of Mercury, this noise is below the estimated signal for BS population; nevertheless it is not negligible especially in the illuminated side and careful analysis is required.

Other wavelengths can produce different noise-signals that will be estimated, but they are probably negligible.

The noise signal on the MCP is called “dark current” and represents the number of counts per seconds and per square centimeters in the absence of any signal. The nominal value for this noise is 1 cm^−2^ s^−1^ or more. With a total MCP area of about 30 cm^2^, there are about 30 counts/s. However, this noise, being “white noise”, scales as the square root of the integration time.

The reflected SW intensity at MPO is of the order of 10^8^ 1/(cm^2^ s sr) for 1-keV H^+^ and <10^7^ (cm^2^ s sr)^−1^ for 4-keV He^+^ and $<10^{6}$ (cm^2^ s sr)^−1^ O^+^. Recent MESSENGER/FIPS results show that at low altitudes (close to the MPO periherm) above the cusps, the mirrored particles are much less than the estimated ones since probably the mirror point is at higher altitudes (Raines et al. 2015). These particles must be deflected before reaching the MCP, but also before passing the ELENA entrance since they could produce additional neutral population generated by ion-sputtering and back-scattering inside the instrument.

**Fig. 19:**
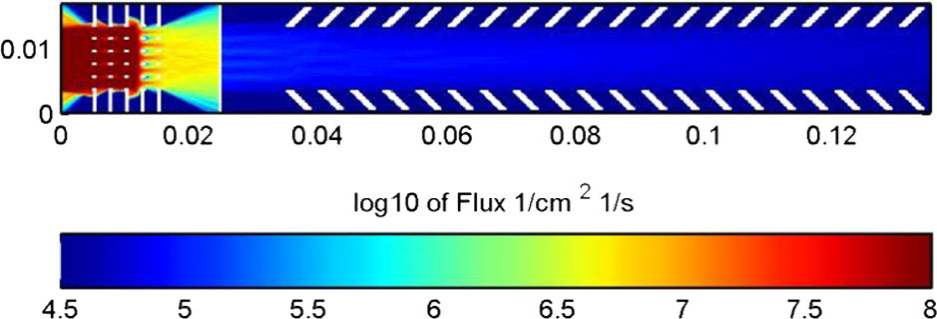
Ion flux at the instrument entrance suppressed by the −1000 V/+1000 V charge stopping deflectors

The ion deflectors at the entrance and inside the ELENA box minimize the noise due to the charged particles (see Fig. 19). To obtain a N < 1% S, the required ion rejection R_ion_ is of the order of 99.5% for protons and 90% for heavy ions. In the case of extreme events, during strong CME, the energies and fluxes could be double; hence, possible background could be higher, but also back-scattering signal would increase of a similar factor.

Noise due to the neutral generation by ion-sputtering and back-scattering inside the instrument is estimated to be <100/s.

### Strofio

**Fig. 20:**
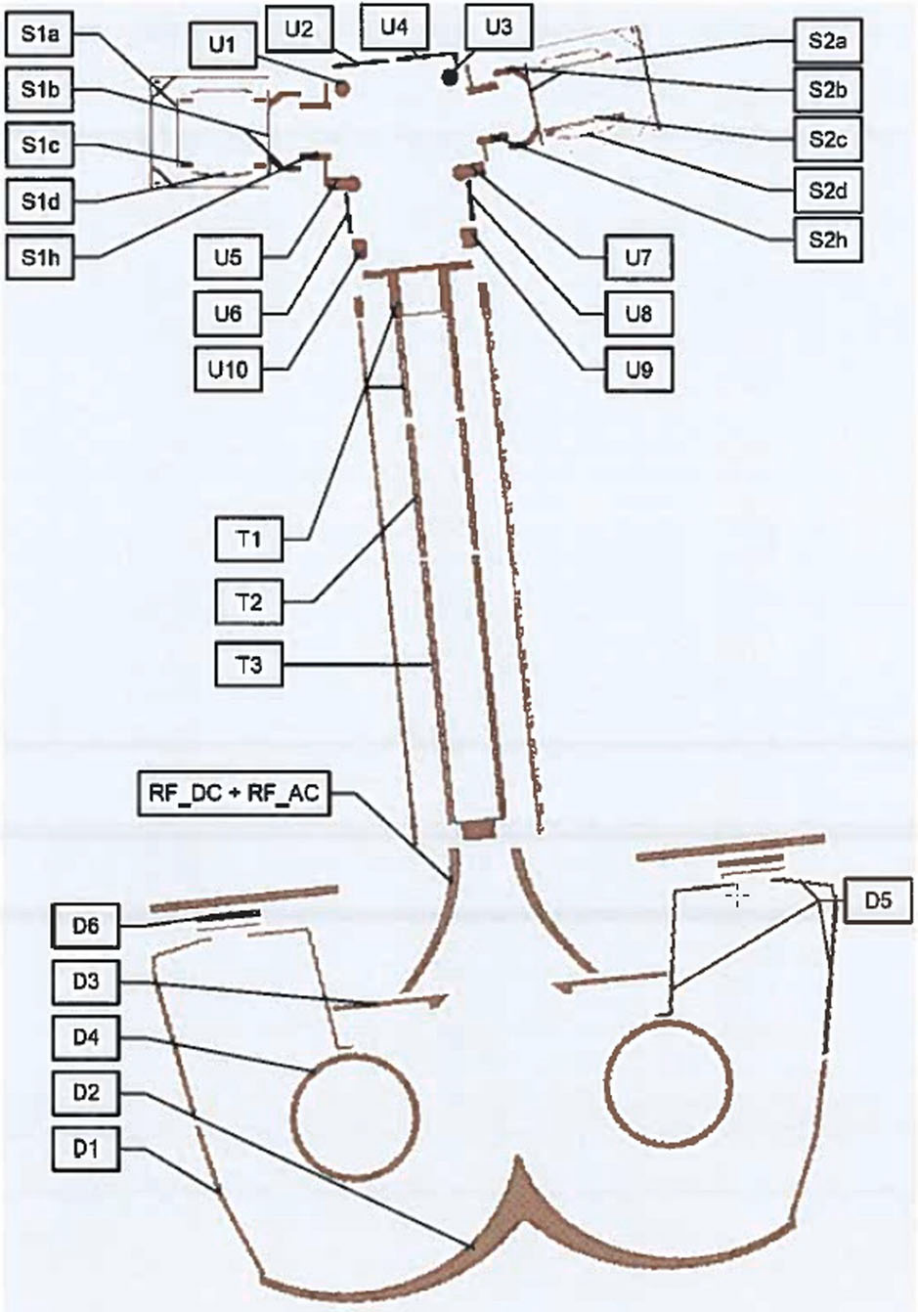
Simion Model of Strofio, showing the 30 different electrodes, whose voltages were determined and optimized during the calibration activities

Strofio is a neutral and ion mass spectrograph that determines particle mass-per-charge (*m*/$q$) by a time-of-flight (TOF) technique. The name comes from the Greek word *Strofi*, which means “to rotate”: the phase of a rotating electric field “stamps” a start time on the particles’ trajectory and the detector records the stop time. Strofio is characterized by a high-sensitivity (0.14 counts/s when the density is 1 particle/cm^3^). The mass resolution ($m$/$\Delta m\ \geq $ 84) is achieved by fast electronics and does not require tight mechanical tolerances. Figure 20 shows a SIMION model of Strofio, along with the naming scheme for the electrodes whose voltages were optimized during calibration.

#### Science Performance Analysis

Mercury’s exosphere is unique; it is not a typical exosphere made up of atoms and molecules escaping off the top of an atmosphere, from the exobase. In Mercury’s surface-bounded exosphere, the neutral particles emitted from the surface move on Keplerian trajectories and mostly return to the surface. Wurz and Lammer (2003) performed extensive Monte Carlo simulations of neutral particle trajectories that indicate that particles reaching the spacecraft come from a footprint on the surface roughly equal to the spacecraft altitude. Examining the characteristics of the exosphere will enable us to explore and probe the different processes responsible for ejecting the atoms from the surface.

**Fig. 21:**
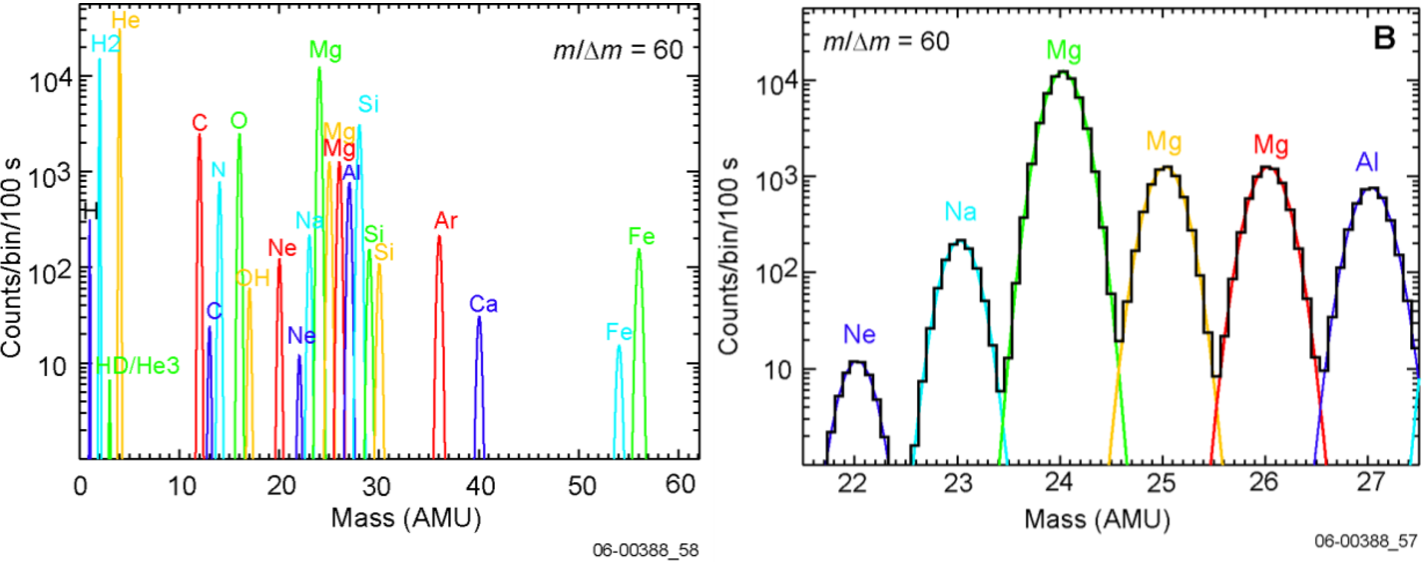
Expected Strofio performance at Mercury. Both elements and isotopes will be resolved in Mercury’s exosphere

The 2.4-h orbit of MPO will enable the mapping of the spatial distribution and the temporal variability of the Na present in the dayside and night side exosphere. To fully characterize the temporal and spatial variability of Mercury’s exosphere, correlated observations from both remote and in-situ are essential. Measurements of Na are required to enable these correlations. A mass resolution of m/$\Delta $m of 60 or better is achievable with an optimized use of available resources and clearly enables resolving Na from the more abundant Mg (Fig. 21B). This constitutes the level 1 requirement for mass resolution.

**Fig. 22:**
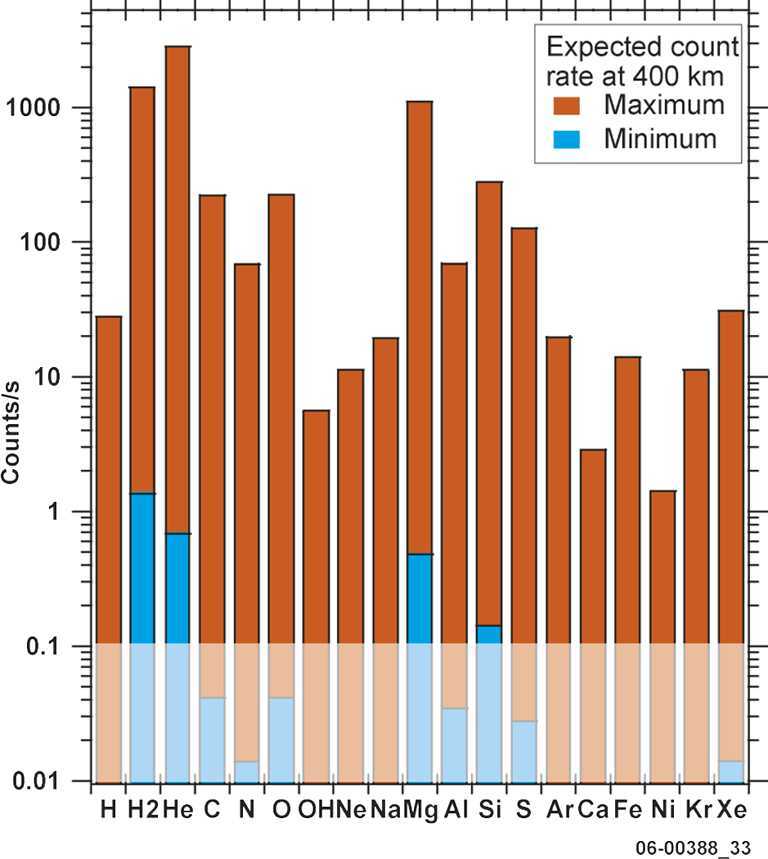
Strofio’s count rates at 400 km altitude, estimated from Table 1. Background is estimated at the 0.1 count/s level

In Fig. 22, Strofio counts rates at 400 km are estimated for density ranges of Table 1.

Sensitivity is defined here as the ratio of measured countrates and the particle density: the higher the sensitivity the more counts the sensor will detect for a given local density. Figure 22: countrates measured by Strofio as function of the emission current and chamber pressure.

Illumination on the MCP and the resolution were checked to prove that overall sensor properties were consistent.

As the expected densities in the exosphere of Mercury are extremely low (see Sect. 2.2), Strofio design was optimized as to achieve a very high sensitivity: for a particles density of 1/cm3, Strofio will produce a countrate of 0.14 cts/s; this corresponds to a sensitivity in excess of 1mA/Torr. This very high sensitivity is achieved by a tailored ionization source design, as well as from Strofio’s continuous mode of operation: every ion produced has the same probability of being detected, avoiding the need for scanning or filtering.

##### Mass Resolution

**Fig. 23:**
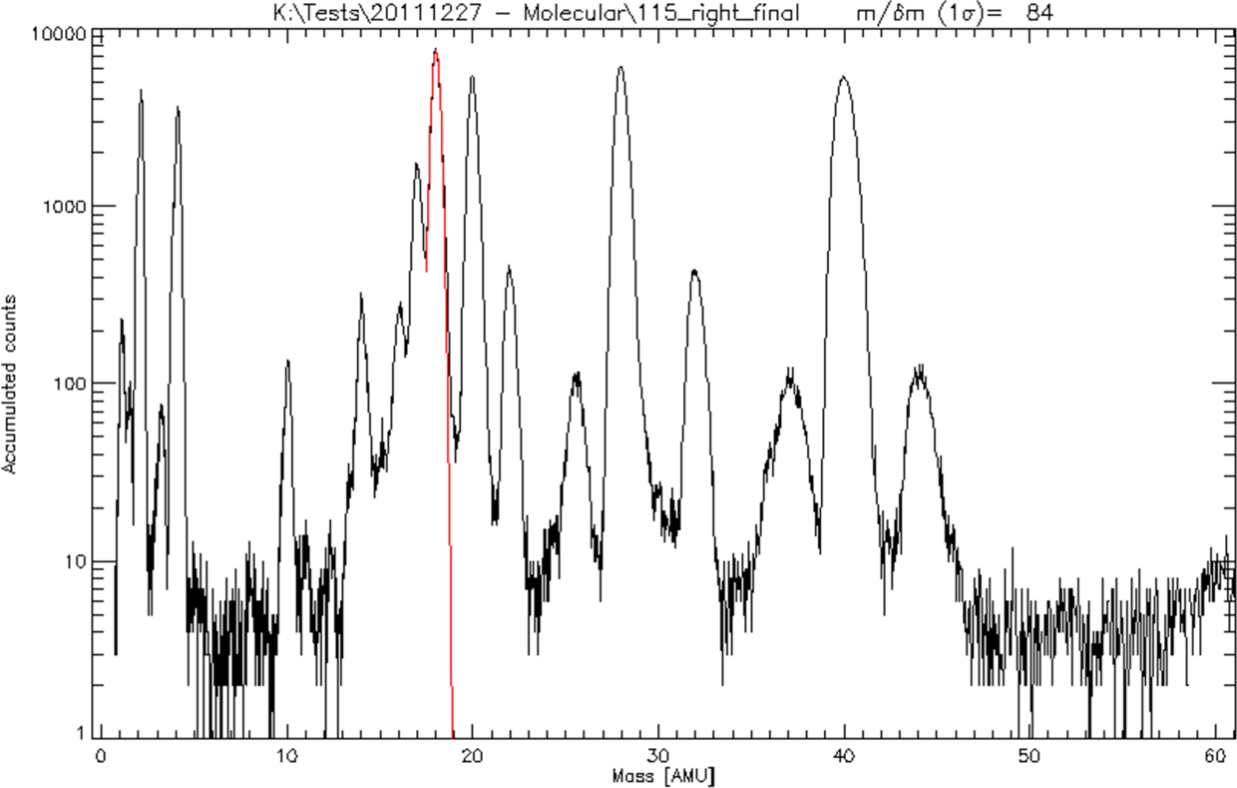
Mass spectrum of H_2_, He, Ne, N_2_, Ar, O_2_, CO_2_

During the same set of measurements that determined the efficiency of Strofio, mass resolution and mass range were constantly monitored to ensure that the efficiency is achieved at the same time as resolution and mass range. It is always possible to trade efficiency for resolution by selecting the velocity space to be measured, and for Strofio resolution and mass range are directly correlated. Figure 23 shows one of the mass spectra collected during the efficiency run, which depicts the characteristics of the sensor: total pressure was 2.8e–8 Torr and the ambient gas was an equal mixture of H_2_, He, N_2_, Ne, and Ar with air (N_2_, O_2_, CO_2_) and water vapour (H_2_O and by-products) coming from the rest gas in the chamber. Note also the many fragmentation products of the various molecules. The peak at m/q = 10 is doubly ionized Ne.

Optimization of the voltages was aimed at the water peak. The result was a mass resolution m/$\Delta $m of 84.

##### Mass Range

As can be seen in Fig. 23, in its primary mode Strofio covers masses in the range 1-64 AMU. If masses of the order 72-90 were present, they would show in the apparent range 5-11: this is the so called “race-track” effect, where particles that take longer than the period of the dispersing wave (here 195 kHz) appear as small masses. Close analysis of the range 5-11 would enable us to detect higher mass species, although they are not expected in the exosphere of Mercury.

The mass range can be easily expanded or reduced in flight, by changing three DC potentials. This may be interesting, for example, to enhance the mass resolution if no noticeable signal is measured above say mass 50, or to focus Strofio on the analysis of heavy masses (50-100) if they indeed were present.

##### Background

Several sources contribute to Strofio’s background: thruster firing, spacecraft out gassing, Lyman-$\alpha $, low-energy ions, and high-energy penetrating particles.

*Thruster firing background*. The ESA Rosetta total pressure sensor measured the response of the ambient pressure to the spacecraft thruster firing (Graf et al. 2008). The total pressure measurement increased by more than 2 orders of magnitude every time the spacecraft fired its thruster. The duration was short (minutes) and the pressure was observed to go down to background within 2 h of the manoeuvre.

*Spacecraft outgassing*. One of Strofio’s apertures is not affected by spacecraft outgassing as no part of the spacecraft is in direct field of view of the experiment. However, the other entrance views part of the spacecraft. The effect is that during half a Mercury year, when the ram direction is along the spacecraft, measurements done by Strofio will be affected. The expected pressure around the spacecraft is 10^−11^ mbar based on measurements on the Rosetta spacecraft (Graf et al. 2008; Schläppi et al. 2010). The background in Strofio strongly depends on the characteristics of the surfaces in the field of view.

Instrument outgassing. One of the well-known issues associated with mass spectrometers is hydrocarbon contamination from residual gas, which Strofio is also likely to encounter. Three strategies to mitigate this problem, used in past mass spectrometers (Balsiger et al. 1998, 2007), are planned on Strofio:

Only inorganic parts were used in the ionisation source.

We allow the source to be exposed to direct sunlight twice a year to bake out residual gas. The temperature of the ionizing source will reach $280\ ^{\circ}$C.

Separate vents will be provided for both the ionisation source and the ToF optics.

Lyman-$\alpha $. MPO is a nadir-pointing 3-axis stabilized spacecraft. Mercury’s shine will never penetrate the aperture, because Strofio always points normal to nadir. At Mercury’s orbit, the Ly-$\alpha $ from the Sun is more than 10 times stronger than at 1 AU. Twice per Mercury year, the Strofio entrance will point toward the Sun. To avoid MCP overload, Strofio will be turned off at these times, with a predicted loss of coverage less than 10%. The galactic and interplanetary UV background (1000 Rayleigh) will enter Strofio, but the photons must non-specularly bounce two times (once in the source and once in the reflectron) to get to the detector. We estimate this background at 0.01 counts/s/pixel, which is negligible.

High-energy penetrating ions. The background due to high-energy ions (>10 MeV) from galactic cosmic rays (GCR) and worst-case solar particle events (SPEs) at 0.3 AU are expected to be between 1 particle/cm^2^/s/sr and 10^4^ particles/cm^2^/s/sr, respectively (Mewaldt et al. 2001). The GCR background in each detector pixel (∼ 0.1 cm^2^) will be less than 0.1 counts/s. On the other hand, during an intense SEP, the background rate in each pixel can be as high as 10^3^ counts/s. Because BepiColombo will arrive at Mercury in late 2025 during the ascending phase of the Solar cycle, based on the current solar minimum, we expect to encounter few major particle events per year. In addition, the highest-energy component of the SEP typically lasts for only a few hours.

### MIPA

#### Science Performance Analysis

##### Expected Particle Fluxes and Count Rates

**Fig. 24:**
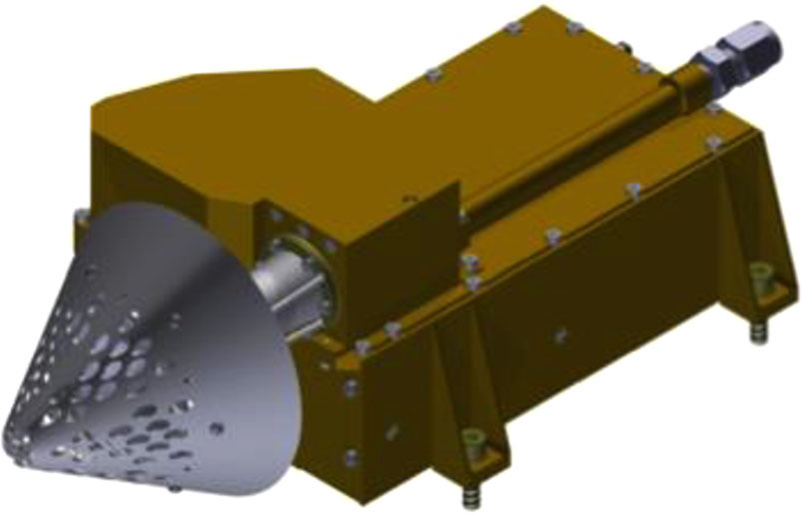
MIPA model with aperture top hat

MIPA (Fig. 24) will monitor two main precipitating populations: (1) the SW in the cusp region and (2) accelerated ions of magnetospheric origin. Using the Helios SW measurements at the Mercury orbit reported in Burlaga and Ness (2001) and picking the peak density of 200 cm^−3^ and the peak velocity of 700 km/s, the maximum SW flux is of 1.4⋅10^10^ cm^−2^ s^−1^. As a minimum requirement for precipitating flux of the magnetospheric ions important for sputtering and back-scattering, we take 10^5^ cm^−2^ s^−1^ following Delcourt et al. (2003) calculations of fluxes of Na^+^ with E > 0.5 keV. Thus, the total flux requirements are: minimum >10^5^ cm^−2^ s^−1^ and maximum <10^10^ cm^−2^ s^−1^. The expected count rates for 1% efficiency and the geometrical factor ∼10^−4^ cm^2^ sr are the minimum >0.1 s^−1^ and maximum < 10^4^ s^−1^. This is well within the range of the counting electronics and detectors. Note Carbon Channel Electron Multiplayer (CCEM) can provide count rates up to 1 Mhz = 10^6^ s^−1^. The MIPA dynamical range established in calibrations is 6 orders of magnitude (see also Wieser and Barabash 2016).

##### Angular Coverage and Resolution

The MIPA bore-sight is looking to the ram or anti-ram direction and covers a hemisphere (some small blocking will occur from protruding spacecraft elements around the sensor).

Ions which are within the loss cone are sampled by an instrument even with a limited field of view, but the complete loss cone may not be fully sampled. The attitude of the instrument field of view can a posteriori be compared with measured magnetic field directions and corrections for the limited field of view be made. We have therefore put the main emphasis on determining if part of the loss cone population is within the field of view of the instrument. The loss cone at the MPO altitude is almost always greater than 20^∘^, i.e., normally exceeds the MIPA pixel size. Telemetry constraints will certainly require the MIPA measurements to be of rather low resolution during most of the time. MIPA should however be run in a high angular resolution mode (< 25$^{\circ}{\times}25^{\circ}$) for some intervals to determine the characteristics of the near-Mercury fluxes of SW origin ions.

**Fig. 25:**
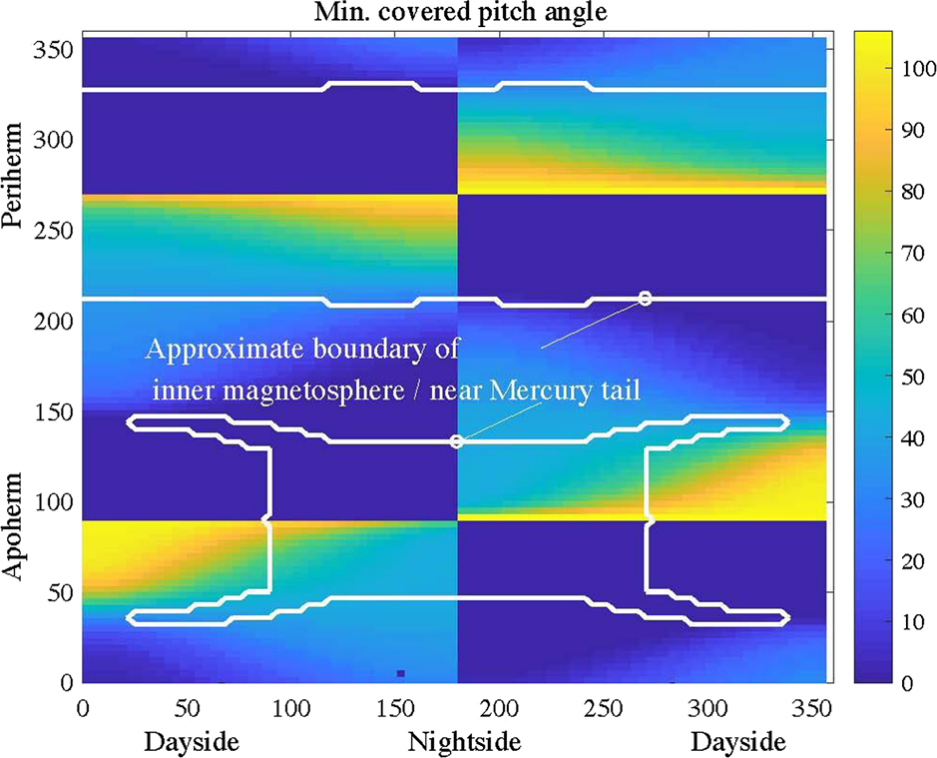
Min pitch-angle covered by MIPA as a function of the beta-angle and true anomaly angle. $(0,0)$ corresponds to the noon-midnight orbit with the pericenter at the subsolar point

**Fig. 26:**
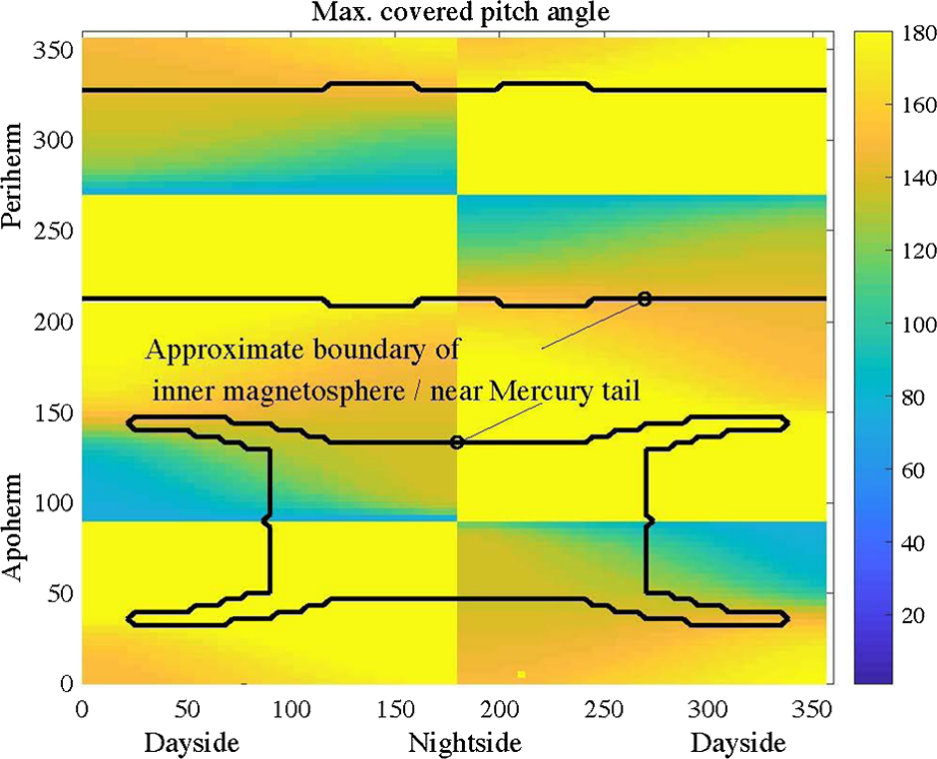
Max pitch-angle covered by MIPA as a function of the beta-angle and true anomaly angle. $(0,0)$ corresponds to the noon-midnight orbit with the pericenter at the subsolar point

**Fig. 27:**
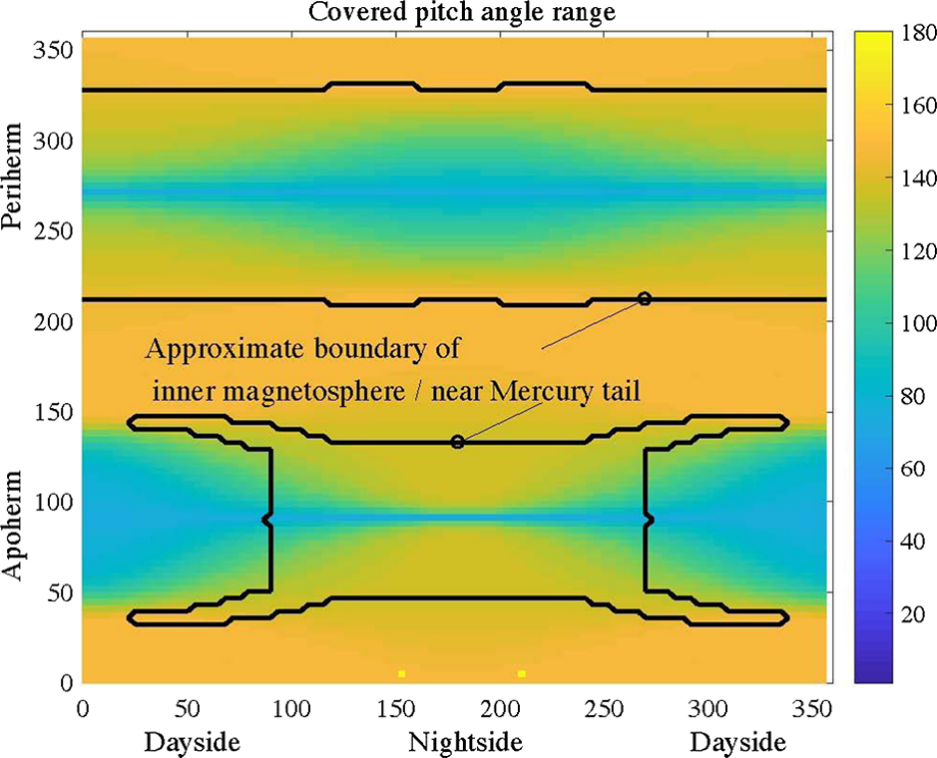
The pitch-angle range covered by MIPA as a function of the beta-angle and true anomaly angle. $(0,0)$ corresponds to the noon-midnight orbit with the pericenter at the subsolar point

The pitch angle coverage of MIPA is shown in Figs. 25, 26, 27. Figure 25 shows the minimum pitch angle observed by MIPA, where we have defined 0^∘^ as precipitating particles. Figure 26 shows the maximum pitch angle, where values above 90^∘^ indicate reflected (mirroring) particles. Figure 27 finally shows the total pitch-angle range, i.e. the result shown in Fig. 25 minus the result shown in Fig. 26. What can be seen is that as the spacecraft crosses the equator (at 90^∘^ and 270^∘^ co-latitude) the spacecraft switches from mainly observing precipitating ions to mainly observing mirrored ions. Coverage of precipitating ions is very good in one hemisphere (typically from about 0^∘^ to 90^∘^) and low in the other hemisphere. This is due to the blockage by the spacecraft itself and its flip-over once per one orbit revolution around the planet. So around equator crossings the coverage of precipitating ions is either perfect or not good. Over the polar caps (co-latitudes of 0^∘^ and 180^∘^) the total pitch-angle range covered is very good. This can be compared to the simulation results by Massetti (Fig. 9), where the typical pitch-angle range is from 20^∘^ to 140^∘^ at high latitudes and from 40^∘^ to 120^∘^ at low latitudes. The pitch-angle coverage of MIPA is therefore well suited for the science requirements of SERENA.

The above discussion concerns pitch-angle coverage. Much of the blocking, in particular due to the magnetometer boom, will in practice block certain gyro-phases for certain pitch-angles. Since ion distributions are isotropic over gyro-phase, the blocking has a much smaller impact on the range of pitch angles sampled.

The angular coverage of MIPA has been simulated and tested in laboratory conditions. The total pixel size and field of view is determined by an electrostatic entrance deflection system in combination with an aperture hat that provides thermal shielding and which is tuned to attenuate the SW fluxes to obtain fluxes in a suitable range for the instrument.

**Fig. 28:**
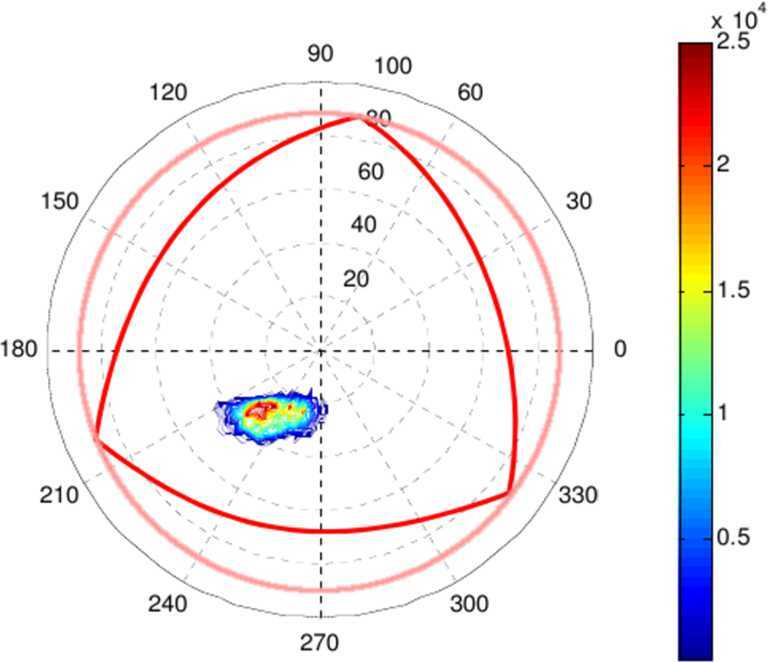
Field of view of MIPA (red line). The colour scale (in arbitrary units where highest value corresponds to 100% transparency) shows a pixel example

**Fig. 29:**
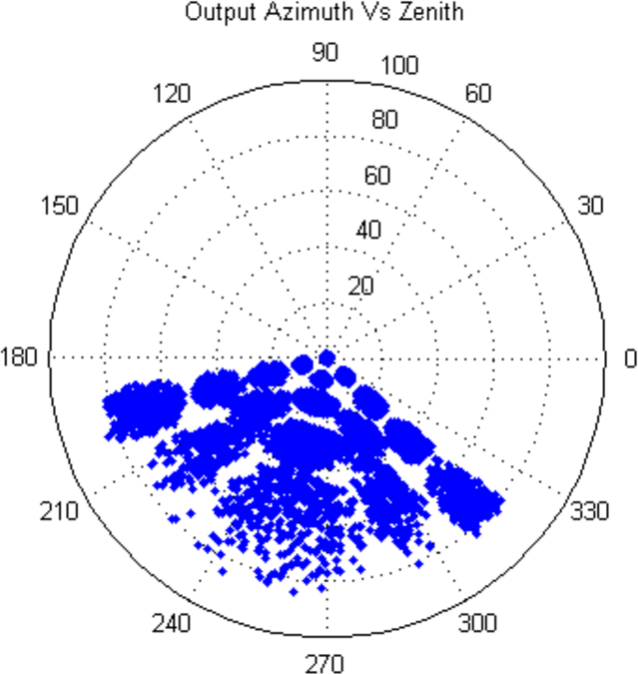
Simulation of primary pixels of the MIPA instrument for one of three rotationally symmetric sections of the instrument

**Fig. 30:**
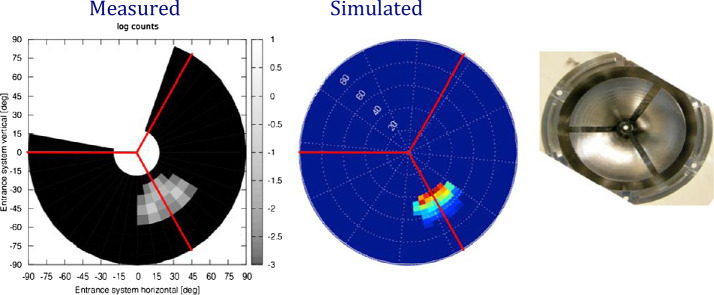
Comparison between laboratory results (left) and simulations for the field of view of one MIPA pixel. The right hand panel shows a photo of the instrument

The near-2$\pi $ field of view of the instrument is illustrated in Fig. 28. The shape of different primary pixels is shown in Fig. 29. The pixels shown are simulation results for 1/3 of the field-of-view. The other 2/3 of the field-of-view are rotation symmetrical. Figure 30 finally compares the laboratory test results for one pixel (left panel) with simulation results (middle panel). The right panel shows the actual instrument. The black region in the left hand panel shows the angular range covered by the calibration chamber turntable. This region has been illuminated with an ion beam. Grey pixels shows the relative response of the selected pixel.

The laboratory tests show clearly that the simulations are a good representation of the actual instrument performance. Laboratory tests and simulations together show that MIPA meets its specifications and will be able to provide data with sufficient angular coverage and resolution for the science mission.

##### Background

The choice of the START and STOP surface materials makes the photon detection efficiency at START and STOP less than 1%. Therefore the solar photon flux ∼2×10^13^ cm^−2^ s^−1^ impinging on the entrance slit of an area of 0.03 cm^2^ results in a correlated count rate of 1.5⋅10^−6^ s for the 1 μs TOF window. That is much below the expected count rate from the ion flux.

### PICAM

The PICAM (Planetary Ion CAMera) ion mass spectrometer operates as an all-sky camera for charged particles (Vaisberg et al. 2001) allowing the determination of the 3D velocity distribution and mass spectrum for ions over a principally instantaneous 2$\pi $ FOV, from several eV up to ∼3 keV energies and in a mass range extending up to ∼132 amu (Xenon). Moreover, due to its omnidirectional sensor, the instrument offers a very efficient duty cycle since no angular scanning is necessary.

**Fig. 31:**
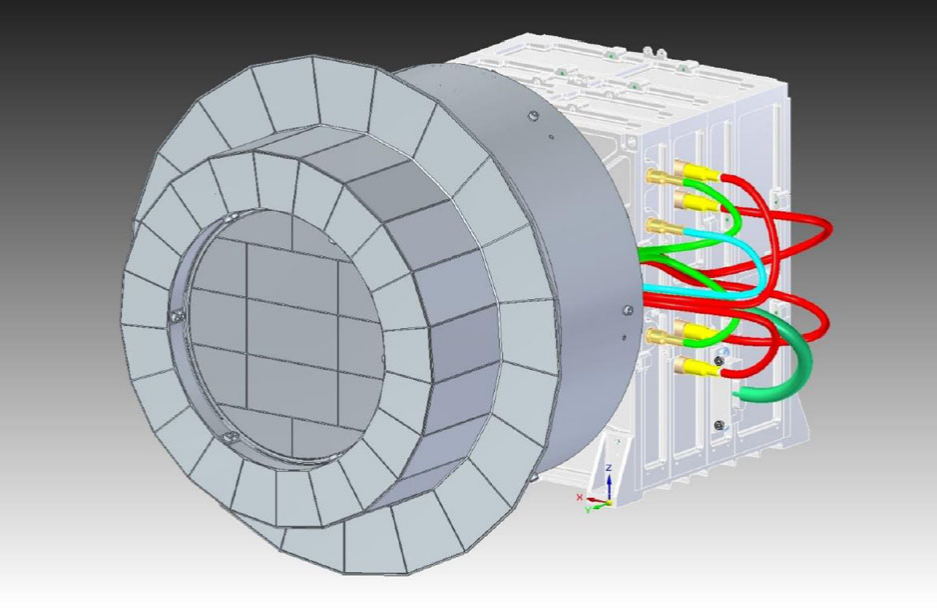
Sketch of the PICAM instrument

PICAM is composed of three main parts: (a) the ion optics, (b) the detector, and (c) the electronic box. The entire instrument has a mass of 2.45 kg and an envelope of approximately 20×24×19 cm in the (x, y, z)-directions and is shown in Fig. 31.

**Fig. 32:**
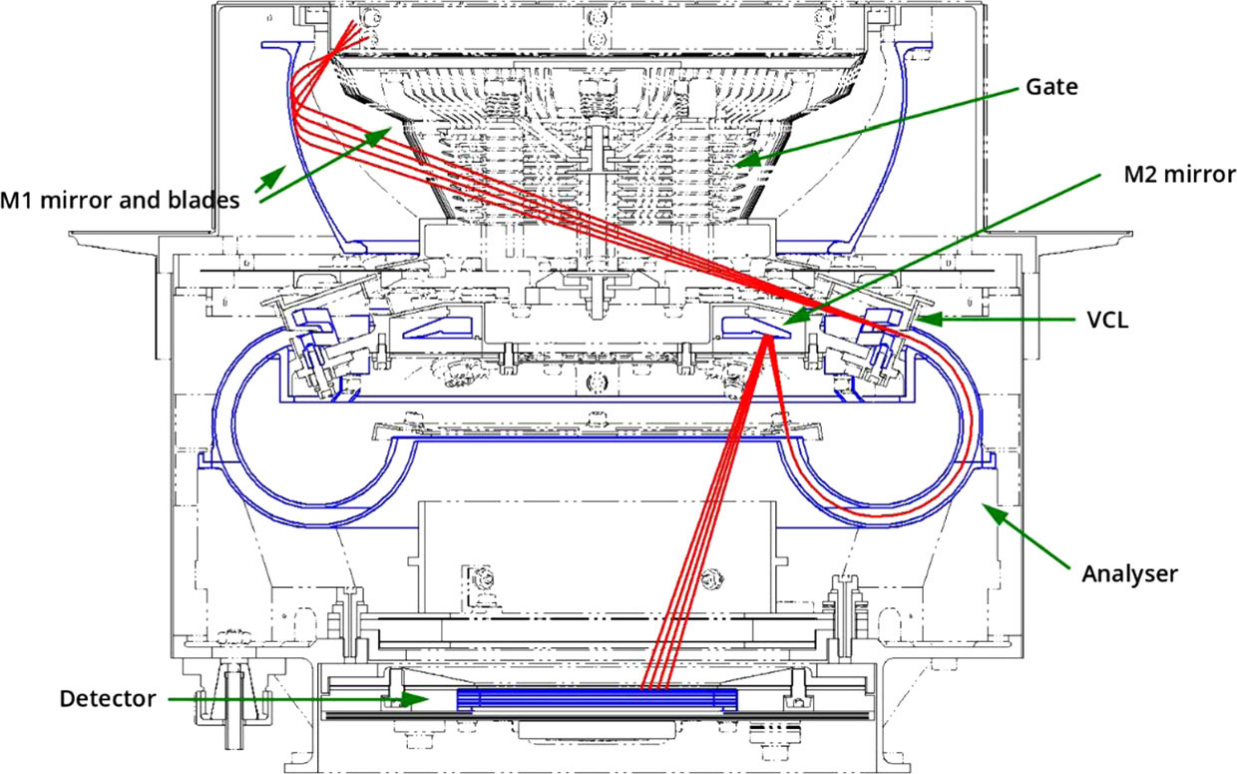
Sketch of PICAM ion trajectories

The general measurement principle is as follows (see Fig. 32): Particles passing the entrance slit at the top of the instrument from any direction, are deflected by an electrostatic mirror M1 through an array of gating blades towards the correction lens located in front of a toroidal electrostatic analyser (ESA). After passing the ESA, the particles are reflected by a mirror M2 into the direction of the detector, consisting of an array of micro-channel plates (MCP). This layout provides energy selection by the pass-band of the electrostatic analyser and results in an inverted image of the hemisphere on the detector, giving a one-to-one correspondence of the particle direction at the entrance and its location on the detector. Finally, the mass analysis is performed by means of time of flight (TOF) measurements: the gating blades provide the “start” signal while the time of impact on the MCP corresponds to the “stop” signal of the individual particles. The characteristics of PICAM are summarized in Table 2.

**Table 2:** Summary of PICAM characteristics

Characteristic	Value
Mass	2.4 kg
Envelope	20×24×19 cm
Power	2.9 – 7.8 W
Field of view (azi., ele.)	6 × 40^∘^ by 6 × 15^∘^
Energy range	∼10eV - 3 keV

#### Ion Optics

##### Mirror M1

**Fig. 33:**
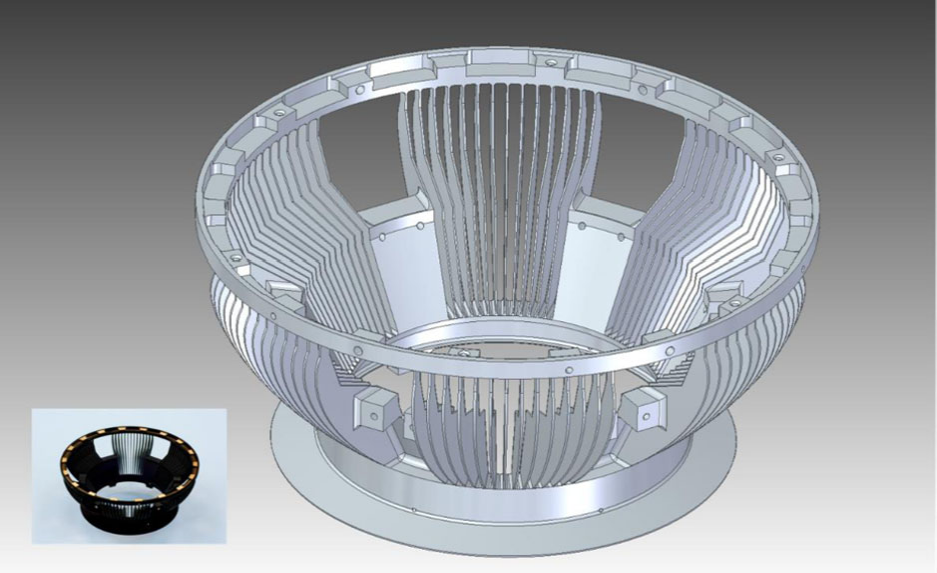
Annular array of blades of the inner part of mirror M1

Key elements of the ion optics are two electrostatic mirrors M1 and M2, the gating blades, and the electrostatic analyser. Particles entering the 0.5 mm wide circular entrance slit are deflected by the mirror M1, which is located immediately behind the slit. M1 is composed of two parts, an external electrode at a positive reflective potential and an internal part at zero potential to produce the desired retarding electric field within the mirror space. The internal part consists of radial blades with an angular “thickness” of 0.35^∘^ and are separated by 3^∘^ (see Fig. 33).

The annular array of blades is divided into six sectors, each of them having an angular width of 40^∘^, gaps of 20^∘^ separate one sector from the other. The blades serve to limit the azimuth angles of the incoming ions in each sector so that only ions with their direction of motion (almost) parallel to the radial direction of the blades can pass M1 and eventually reach the detector. In addition, M1 must map the range of possible elevation angles (∼ 70^∘^) at the entrance slit to ∼ 10^∘^, which can be handled by the interior optics.

##### Converging Lenses and the Electrostatic Analyser (ESA)

The converging lenses (VCL) are located in front of the entrance slit of the ESA, consisting of two parallel plates separated by 2 mm. Their main function is the compression of the elevation angles of the incoming ions from ∼ 10^∘^ to ∼ 3^∘^. By applying a positive voltage, the ion beam can be adjusted to improve the mapping of the ion angular distribution in the detector.

**Fig. 34:**
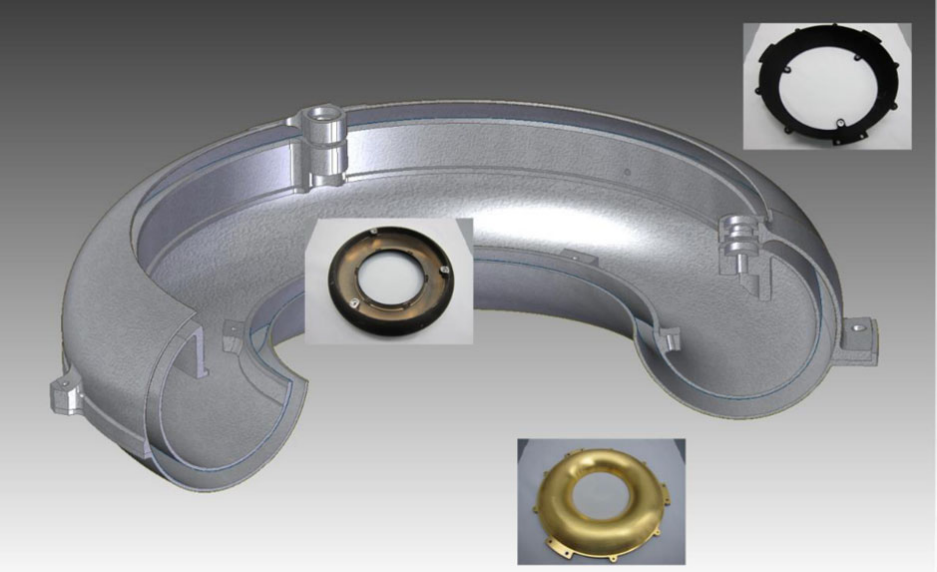
Electrostatic Analyser ESA, consisting of three parts, which are screwed together

Just behind the VCL, the ions enter the toroidal electrostatic analyser through a small annular aperture (see Fig. 34). Depending on the applied voltage between the grounded inner and the positive outer electrode, only ions with an appropriate energy can pass through the ESA, hence the E/q ratio of the exiting ions is known.

##### Mirror M2

**Fig. 35:**
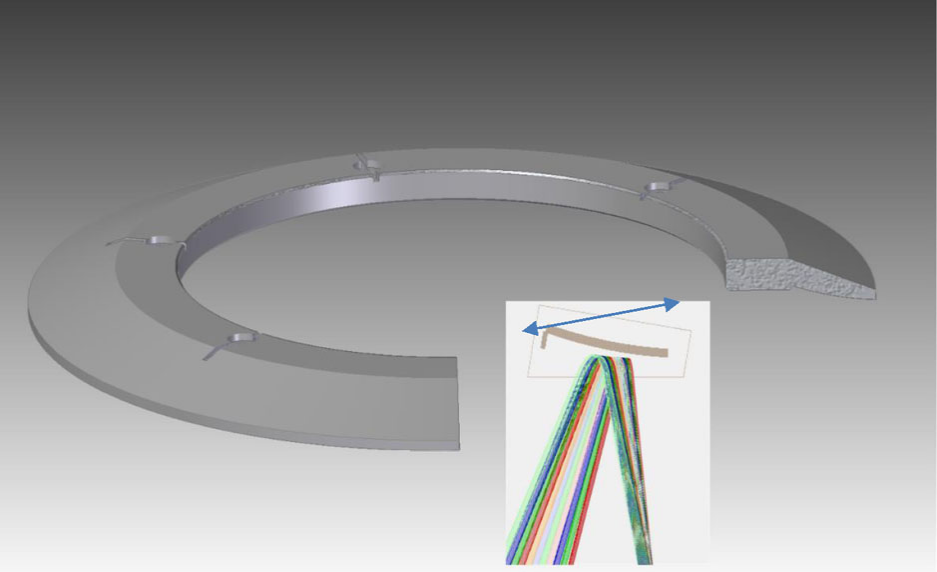
M2 serving as a convex lens to map the ions onto the detector

The mirror M2 is located after the exit slit of the ESA and reflects the ions onto the detector (see Figs. 32 and 35). M2 consists of an external grounded planar grid passed by the ions both before and after reflection, and a positively charged electrode, which actually reflects the ions.

The design of M2 had to meet the requirements that:

- the elevation range of the incoming ions (20^∘^ − 90^∘^) should be mapped on the entire radius of the detector’s MCP by preserving a linear relationship between the radial distance on the detector and the polar angle at entrance,
- the “blurring” of the image due to energy aberration should be kept as small as possible.

##### Gate and Time of Flight

**Fig. 36:**
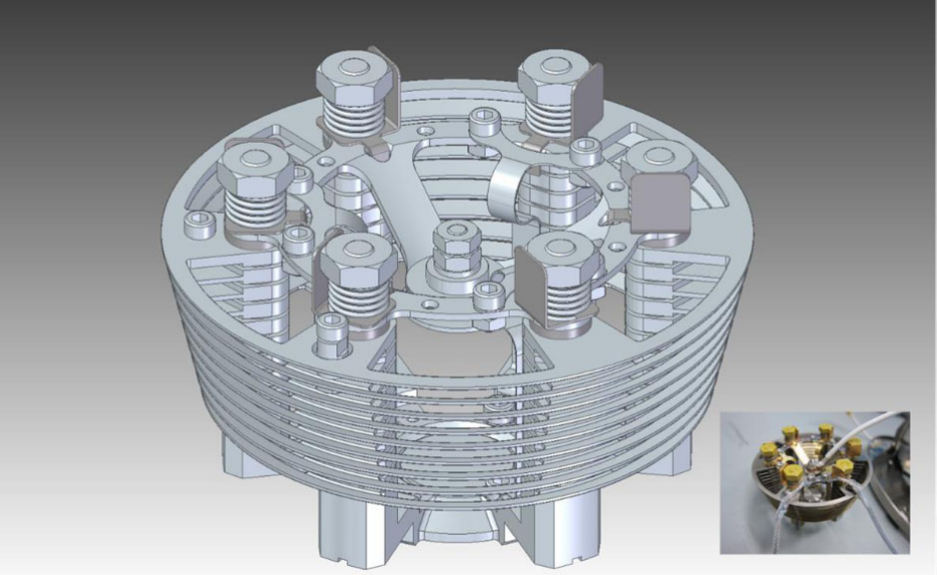
Gating electrodes for TOF measurements

Mass analysis is performed by measuring the time of flight of the ions between a set of gate electrodes located between the primary and secondary mirror M1 and M2, and the time of impact on the MCP. After exiting M1, the ions travel through the gating system, a series of 9 parallel stacked conical electrodes with a thickness of ∼ 0.7 mm each. These gate blades and all following parts of the optics have been optimized to ensure that ions with different elevations, but identical energy have almost identical transit times. In case of TOF measurements, these electrodes serve as a gate for the ions and provide the TOF-“start” signal. In non-TOF mode, the gating electrodes are on ground potential, leaving the ion trajectories mostly unaffected. The detector then continuously counts all ions without mass discrimination. During TOF-mode, a voltage of typically 20 V is applied to five electrodes and a similar negative value to the other electrodes to prevent the penetration of further ions while determining the flight-time of the passed ions (see Fig. 36). The detector provides the “stop”-signals upon the individual impacts of the ions on the MCP. That allows the separation of the ions according to their different mass-to-charge ratios. Knowing also the energy of the ions and assuming the ions to be singly charged, their mass spectrum can be determined.

However, since the gating distinctly affects the trajectories of the ions, some information about their angular distribution is inevitably lost during TOF-measurements. The loss of information occurs during the time intervals of transient voltage at the gate between zero Volt (open state) and maximum voltage (closed state). Ideally, this time should be kept small compared to the duration of the open states of the gate, because the single pulse gating sequence results in a reduction of the effective operating time of the instrument. The opening time $\Delta $T of the gate should be small to achieve a sufficiently high mass resolution, while the closure time T of the gate should be long enough to ensure also the registration of the time of flight of the highest mass ion species. The single pulse mode hence results in a low duty cycle $\Delta $T/T of a few percent of the total time and can only be used when ion fluxes are sufficiently high.

Therefore, to improve the effectiveness of the sensor, especially when ion fluxes are low, a more sophisticated gating sequence based on the Hadamard time mask technique is applied. This technique has been developed in recent years (Brock et al. 2000) in laboratory instruments and has a great advantage since it keeps the mass resolution high and largely increases the effective operational time of the sensor up to about 40%. In Hadamard mode, the gate is opened and closed according to a pseudo-random sequence with 511 code elements. The detected pulses have to be de-convoluted, however, in order to obtain the original time of flight spectrum.

#### Detector

**Fig. 37:**
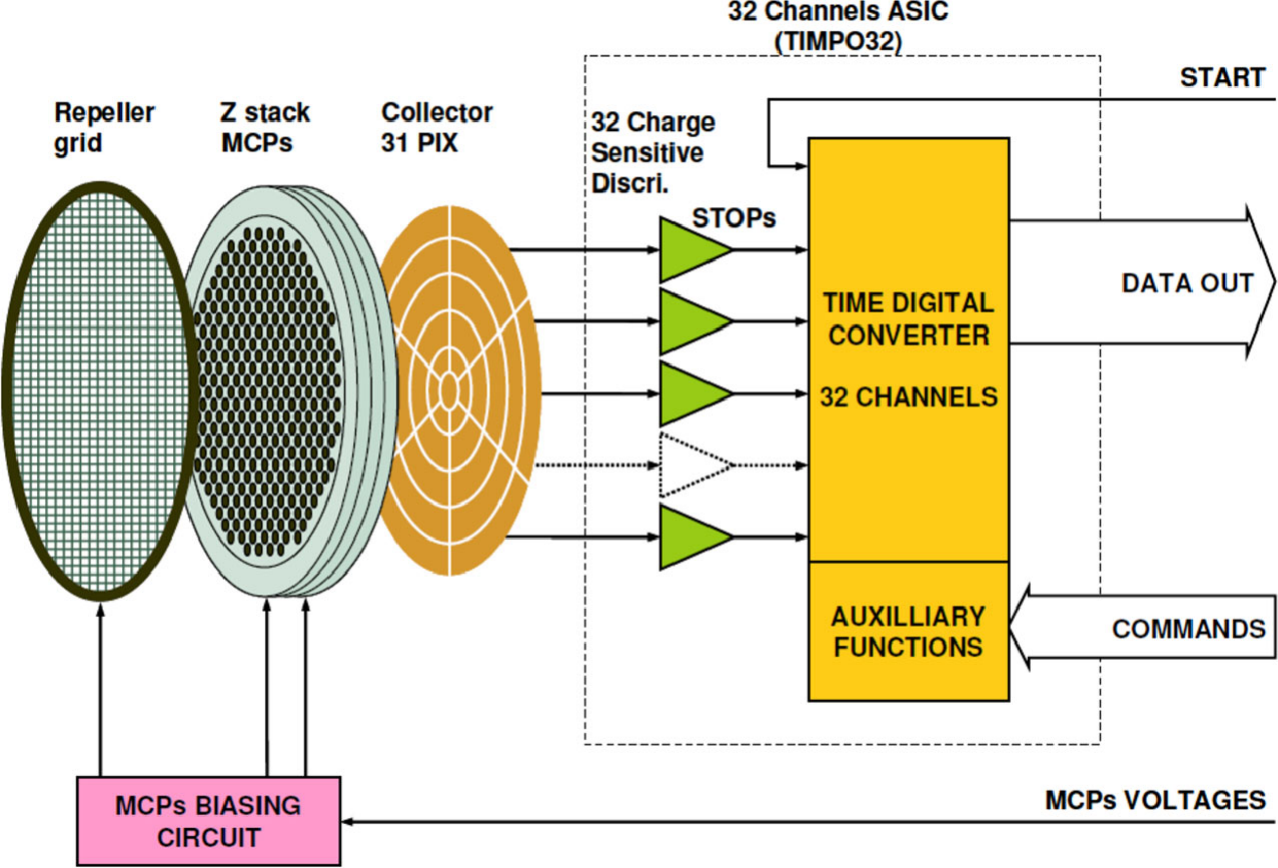
Detector block diagram

After being reflected by M2, the ions travel through a field free region before hitting the detector. A block diagram of the detector is shown in Fig. 37.

The detector is based on a Z-stack of three multichannel plates and a 31 pixels anode followed by a 32 channels ASIC named TIMPO32. The incoming particle induces an electron cloud exiting the MCP stack. This electron cloud is collected on one of the pixels of the anode to become a charge pulse acting as a “STOP” for the TOF-measurement. The “START” information, synchronous with the opening of the gating electrode of the optics, is provided by the gate encoder and driver board (GED) as a voltage echelon converted on a charge pulse similar to those exiting the MCP-stack.

The 32 channels ASIC constitutes the heart of the electronics, where channel 0 is dedicated to “START” pulses and channels 1 to 31 to “STOP” pulses triggered by the 31 pixels.

Charge pulses are discriminated and time stamped with a resolution of 390 ps. TOF-measurement (TOF=TSTOP-TSTART) are computed on board the ASIC, and data are send to the CPU as serial packets of 40 bits for one event.

**Fig. 38:**
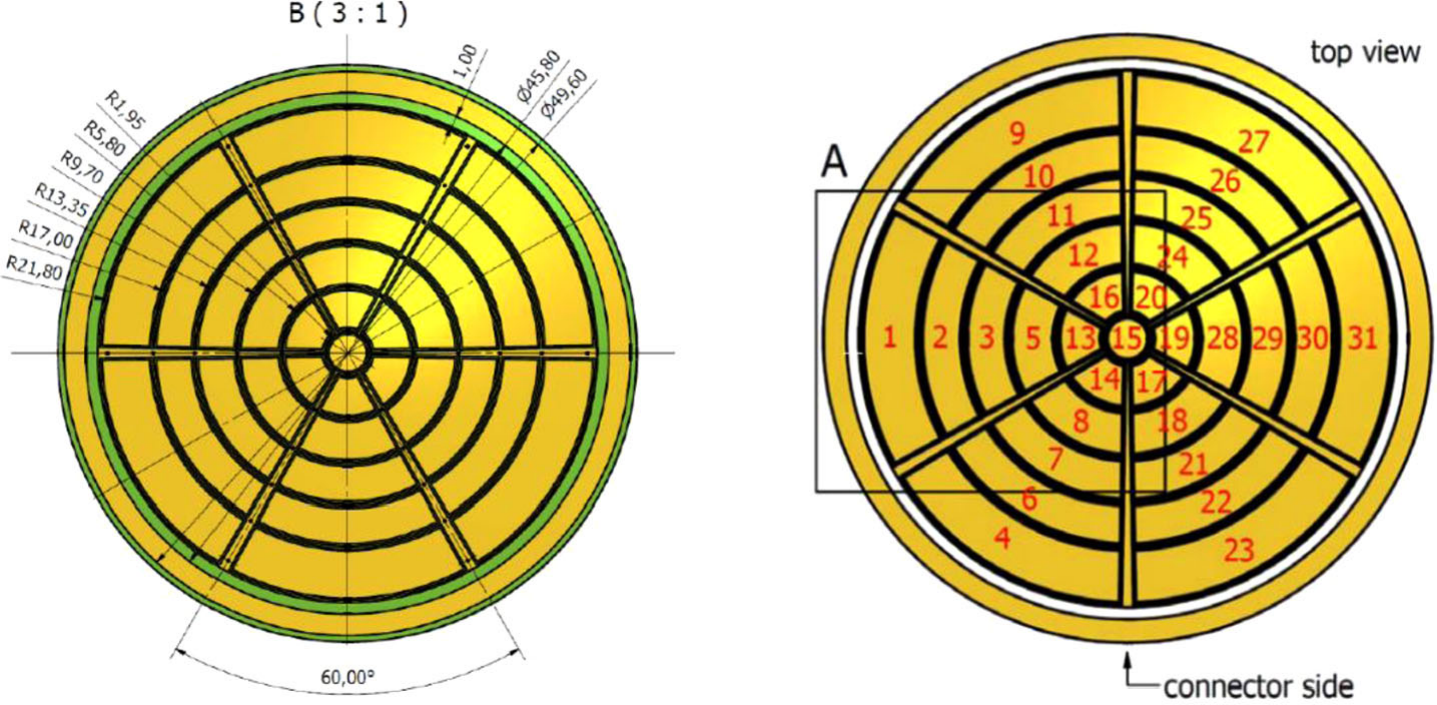
Pixel geometry and mapping of the collector

The anode or collector consists of an array of 31 pixels. The pixel geometry is shown in Fig. 38. Each pixel has an azimuthal width of 60∘ together with an additional circular pixel in the centre. This arrangement covers the whole FOV between $0^{\circ}- 360^{\circ}$ azimuth and $20^{\circ}- 90^{\circ}$ elevation and allows the determination of the direction of the incoming particles.

**Fig. 39:**
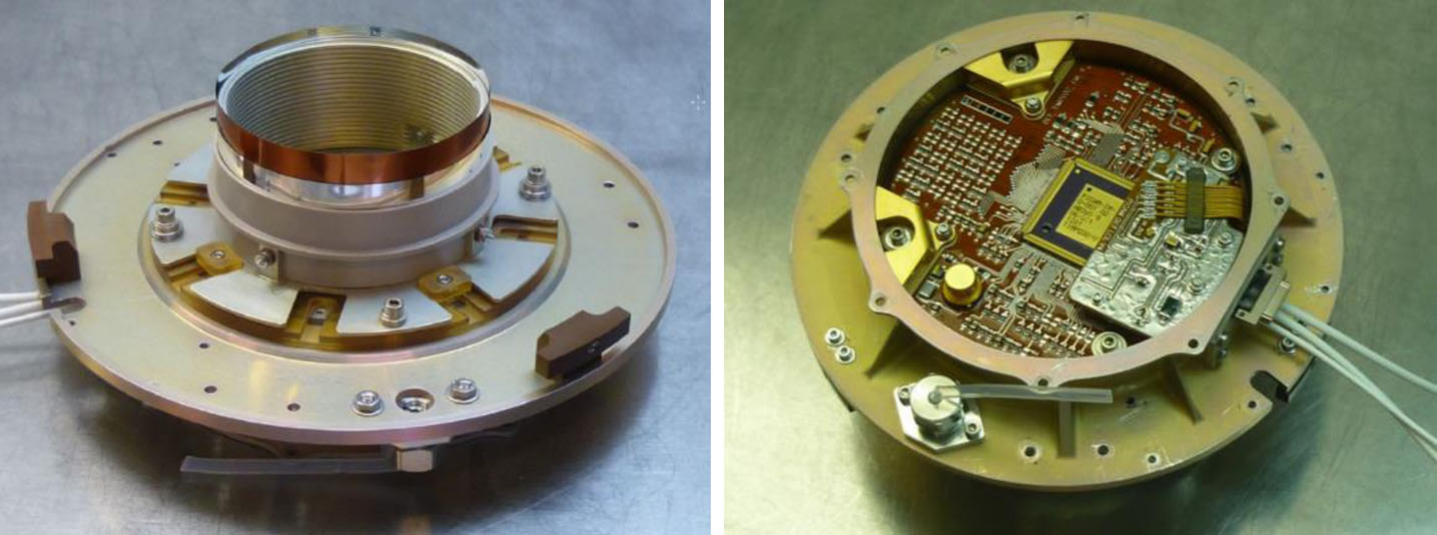
detector top and bottom view with Electronics

Both the collector and the ASIC are mounted on a circular electronics circuit. The MCP and the repeller grid are stacked right above the circuit and hold together with a set of mechanical parts. The whole assembly is fitted into a circular housing (see Fig. 39).

The optics and the detector are powered and controlled by the electronics, which consists of four electronic boards mounted in an electronic box:

- The controller provides the telemetry and telecommand handling, command interfaces for the detector and the gate encoder and driver, on-board health and safety monitoring with the collection of housekeeping data and the data processing of the detection events.
- The power converter is connected to the spacecraft’s 28 V primary power via the SERENA Control Unit SCU. It provides low voltage power to the parts of the Electronics and to the Detector.
- The high voltage converter supplies the MCPs in the detector and the mirrors, grids and the ESA in the optics with high voltages up to 3000 V.
- The gate encoder and driver controls the opening time of the gate for the ions in both the single-pulse mode and Hadamard mode.

### SERENA System. Hardware and Software Configuration

#### System Control Unit General Configuration

**Fig. 40:**
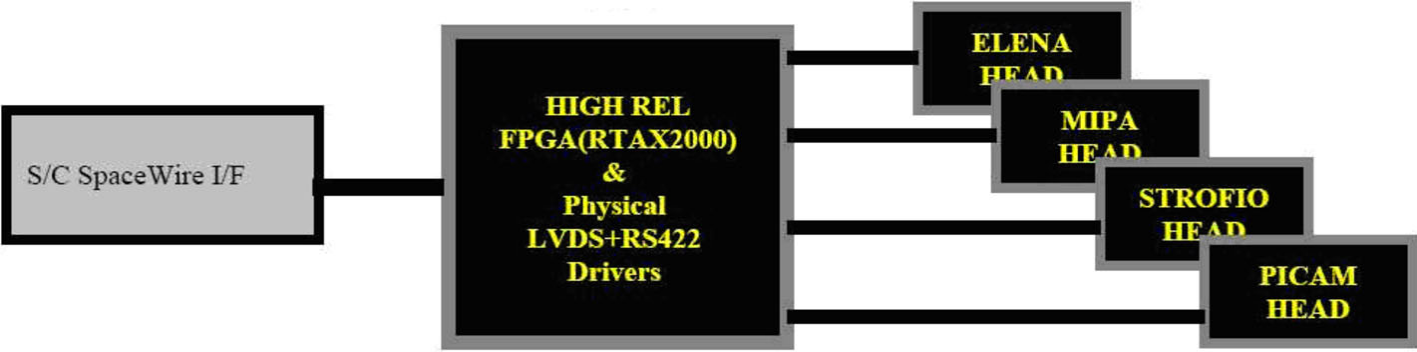
SCU Architecture

SERENA is an instrument composed of 4 units devoted to neutral and ionized particles detection in the Hermean environment, plus a System Control Unit (SCU), see architecture as in Fig. 40, to provide whole package instrument functionality control, memory and computational capability. The standard design techniques of the DHSU (Data Handling and Support Unit) are typical for space applications and are based on the development of computational blocks, independent on the specific H/W platform, and in a hardware description language scaling the performances on the available target technology. Such approach has the advantage to scale the computational performances to the latest radiation-tolerant technology available from the ASIC and FPGA space market, and gives to the project the requested flexibility in spite of minimum allocation budgets. However, for fully saving the high reliability / rad-tolerant profile required for the SCU, the FPGA solution was addressed with the add-on compression algorithm which was operated after having passed the deep health checking testing and excluded by default during in-flight operations.

#### Fault Tolerance Design

The SERENA System does not foresee a cold redundancy within its units. However the experiment configuration is made by two ion particle analyzer sensors and by two neutral particle analyzers. In this respect, if one of such unit fails the correspondent one can still partially fulfil the objective of the mission.

##### SCU

The SCU is non-redundant, while the SpaceWire interface between S/C and the SCU is redundant.

##### ELENA, Strofio, MIPA, PICAM

All sensors of SERENA are non-redundant.

#### Signal and Data Handling Electrical Interfaces

This section describes the NPA-IS system data handling interfaces, i.e. the physical/signal and the character/exchange transmission levels between the different units and the main S/C SpaceWire I/F.

From the MPO S/C p.o.v., the NPA-IS implements a standard SpaceWire Remote Terminal Unit (RTU), which is commanded and controlled by the main S/C Data Handling System. A High Bit-rate (HBR) SpaceWire protocol is implemented between NPA-IS system and the spacecraft I/F according to the ECCSS-E-50-12A standard.

A central hub, implemented in the NPA-IS System Control Unit (SCU), provides the main Point-to-Point communication serial interfaces between the SCU itself and the subsystems. One dedicated bi-directional serial interface is placed between the main NPA-IS system hub and each sensor head unit namely Strofio, ELENA, MIPA and PICAM.

##### Instrument Signal and Data Handling Interface Description

The first kind of interface between SCU and the ELENA-MIPA sub-systems implements a point to point bidirectional LVDS I/F running at 2 Mb/s Bit-rate, and Strofio a UART protocol 38.4 Kbits/s on a physical LVDS layer.

**Fig. 41:**
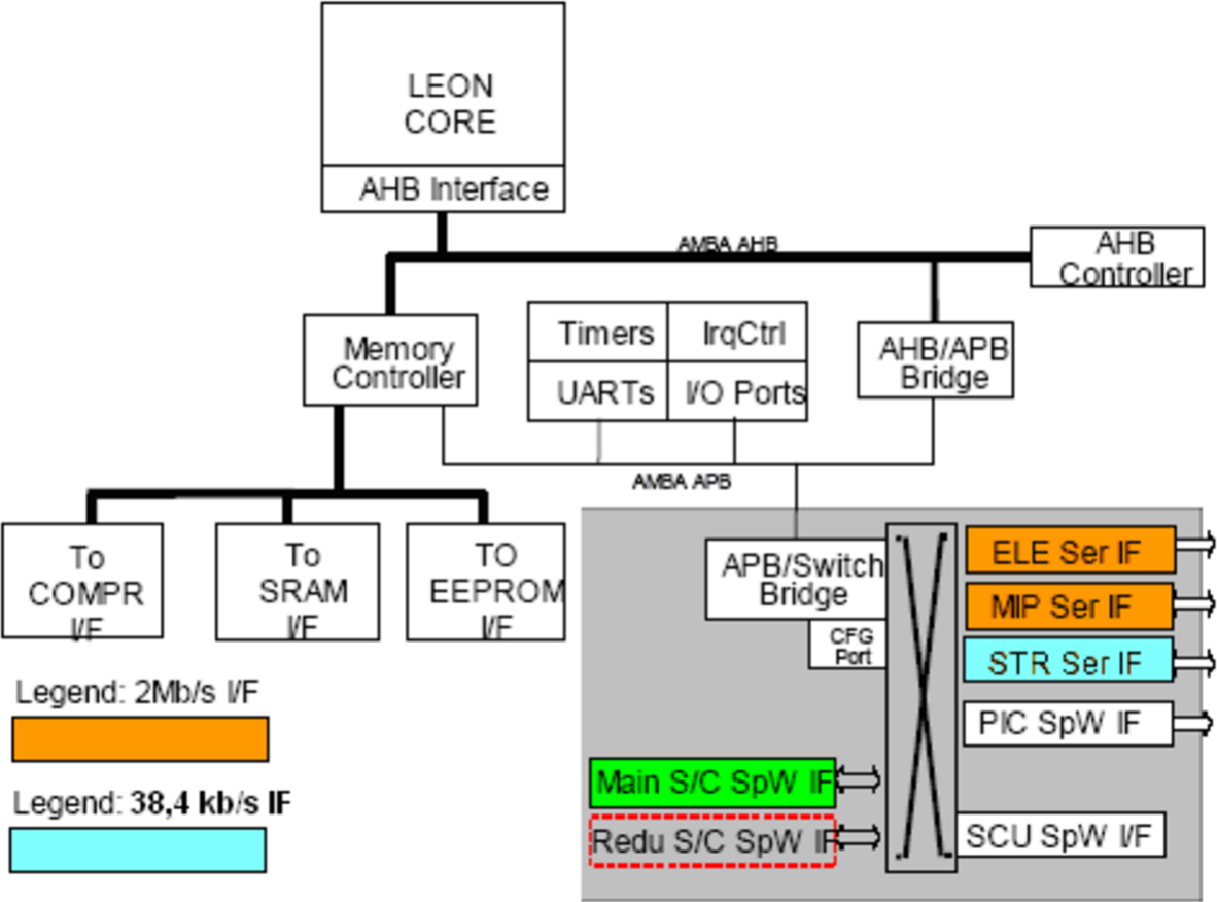
Instrument physical data communication diagram

The second kind of interface provides a 10 Mb/s bi-directional SpaceWire I/F between SCU and PICAM sub-system as shown in Fig. 41.

Regardless of the type, all the subsystems dedicated I/Fs support:

- uplink commands to the different sensor heads;
- uplink data, Look Up Tables (LUTs) & parameters;
- downlink science data from the different sensor heads to SCU system hub;
- downlink housekeeping data from the different sensor heads to SCU system SCU hub.

#### On Board Software

##### Software Concept

Each unit of SERENA is able to operate separately and to achieve its specific scientific objectives, by means of a dedicated computational resources as listed in the following:

- ELENA: a local virtual Leon 3 FT VHDL microprocessor implemented on a local RTAX2000S Actel FPGA device;
- PICAM: local virtual 8085 VHDL microprocessor implemented on a local RTAX2000S Actel FPGA device;
- Strofio: hardwired sequencer onto the local FPGA;
- MIPA: hardwired sequencer implemented onto the local FPGA controlled by SCU;
- SCU: the System Control Unit for interface of NPA-IS suite to the MPO S/C based on a local virtual Leon 3 FT VHDL microprocessor implemented on a local RTAX2000S Actel FPGA device.

The units ELENA, Strofio and PICAM, plus the SCU unit are based on a local microprocessor and therefore they require a dedicated S/W development program.

##### Software Architecture/Design Overview

The SERENA on board management and processing of the commands and of scientific data is performed by a combination of the H/W (FPGA) resident in detectors peripherals and the S/W and the H/W processing units (FPGA), resident in the SCU. The SERENA specific data processing running in SCU is referred as SERENA Data Handling System (SDHS) consists of:

- A Virtual VHDL microprocessor (custom LEON 3 CPU based) with its related instrument application firmware. This is the main high-reliability processor which will work as main SERENA IFE.
- Compression algorithms of application SW for demanding data rate compressions.
- In general the SCU On Board Software is in charge of the following tasks:
- communication with the MPO OBDH;
- management and transmission of all SERENA scientific data;
- transmission of all HK data (SCU and Sensor heads). Limited subsystem parameters processing;
- reception and routing of TC directed to the instrument, coming from ground via CDMU;
- internal management of commands from SCU to peripherals (ELENA, Strofio, MIPA and PICAM);
- handling of periphery context and tables;
- scientific sub-mode switching.

The software is divided into “Reduced” software (a) and “Main” software (b).

##### The REDUCED S/W

The Reduced S/W is a custom Real Time Operating System (RTOS) designed to support only a limited set of functionalities. The software has been developed for maintenance purposes (uploading and/or patching memory areas).

The SCU Reduced S/W is essentially devoted to:

- handle and verify the Ground TCs routing to the peripherals (ELENA, PICAM and Strofio);
- collect and transmit, according to predefined polling sequences, science and housekeeping data from the supported sensors (ELENA, PICAM and Strofio) without the possibility to process them (for instance PICAM science packets cannot be compressed by SCU with reduced S/W);
- provide SCU and sensor monitoring;
- keep the on-board time aligned with the S/C OBT.

For extending its functionalities, a dedicate SCU private command allows jumping in the Main S/W.

##### The MAIN S/W

The Main S/W is designed to meet all the SERENA SCU requirements. It manages all the SERENA data products (science and no-science data) routing these data to the S/C. The Main S/W includes a MIPA-SCU I/F module in order to support MIPA in the different operative modes. Further functionalities are summarized as follows:

- On board data compression (for MIPA and PICAM). The S/W includes different kinds of data compression. Both Loss-Less and Lossy such as: RICE, Huffman and Lin-Log;
- S/C I/F management;
- On board time update;
- HK sampling and monitoring;
- SERENA Units I/Fs management;
- Memory management.

The On Board Software is written in C language and is possible to modify/upgrade it via Telecommand (service 6).

**Fig. 42:**
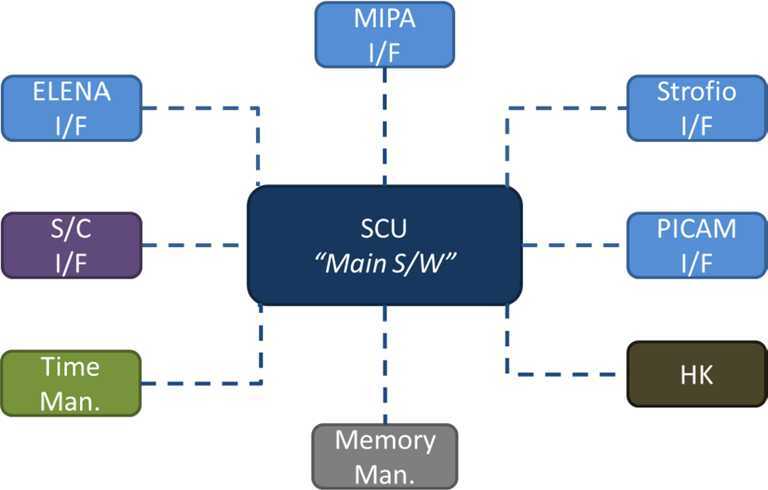
The Main SCU S/W functionalities diagram

The entire Main SCU S/W functionalities are shown in Fig. 42.

##### The on-Board Compression

On-board data compression is a very important issue to maximise the scientific return of SERENA in the MPO platform. Both reversible (loss-less) and lossy compression algorithms are implemented on SCU.

According to the above addressed methodology, the following compressions algorithms were included on SCU S/W:

- Loss-Less type 1. RICE algorithm: for MIPA Science data packets;
- Loss-Less type 2. Huffman: for PICAM Science data packets;
- Lossy.Lin-Log: for MIPA Science data packets (only in binning mode).

##### The Peripheries Firmware

The periphery subsystems are operated by a fixed sequencing of settings, operations, data processing and transmission activities acting at register transfer level. These tasks are achieved by hardware description languages running and embedded in processes hard coded at FPGA level. So far, even if a certain grade of flexibility for setting the different operating scenario and outputs from the sensor will be allowed, the sensors will host only hardwired coded firmware, with the exception of PICAM and ELENA which will embed and FPGA based microcontroller.

##### Redundancy

In order to minimize the budgets, SERENA is a package structured not to foresee H/W redundancy. So far, it does not foresee as well concurrent S/W modules performing on each H/W resource the same task. However, specific error control tasks are run at all levels to avoid data contamination, by means of H/W tools (EDAC correction) and S/W control tools (continuous memory checksums verification on main code and data memories area, watchdog, timeouts control on main intercommunication tasks). The presence of SW compression algorithms allows to perform critical timing consuming tasks as data compression among two different SW resources providing a computational redundancy in this respect.

##### Storage Capacity

All the SERENA sensor units allocate the minimum memory reservoir for supporting the highest data transfer and buffering mode as needed on each unit. Conversely, SCU has the capability to store up to 1 MByte (512 kW), where 100 kW are addressed for the RTOS and all data communication buffering according to the following:

- 2 kW S/C memory buffering
- 150 kW MIPA memory buffering, tables and compression buffer
- 2 kW PICAM memory buffering
- 4 kW ELENA memory buffering
- 2 kW Strofio memory buffer
- 128 kByte ELENA FIFO
- 32 kByte Strofio FIFO
- 512 kByte MIPA FIFO

##### Boot Behavior

After power up, the SCU operates as follows:

- The “Reduced S/W” is the default mode in which SERENA is switched on following a reset or a power-up. From this mode, the unit supports only a limited set of functionalities (mandatory services) and it is generally used for maintenance purposes. The Reduced S/W supports the communication from and to the SERENA Units (except MIPA) but no data compression is foreseen.
- The “Main S/W” is the extended mode that allows SCU to meet all the SERENA SCU S/W Requirements. In this mode, SCU is able to support ELENA and MIPA in the different modes and compress MIPA and PICAM science data. The Main S/W is also designed to sample and analyse the SCU HK parameters and, in case of failure, it runs an emergency sequence.

**Fig. 43:**
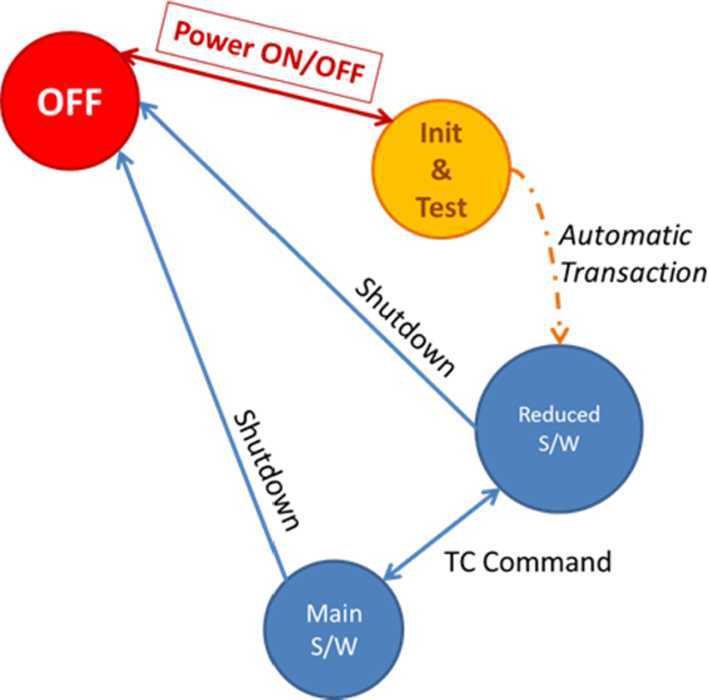
SCU S/W transition mode diagram

The SCU S/W transition mode is sketched in Fig. 43.

## Calibration Tests Summary

### ELENA Calibration and Test

**Fig. 44:**
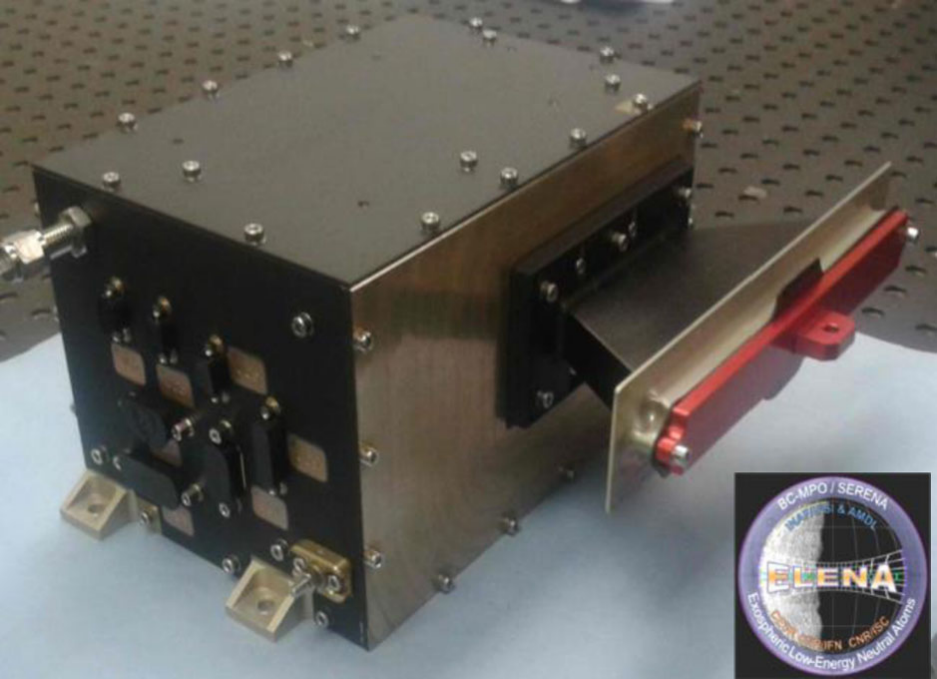
ELENA Proto-Flight Model (PFM)

**Fig. 45:**
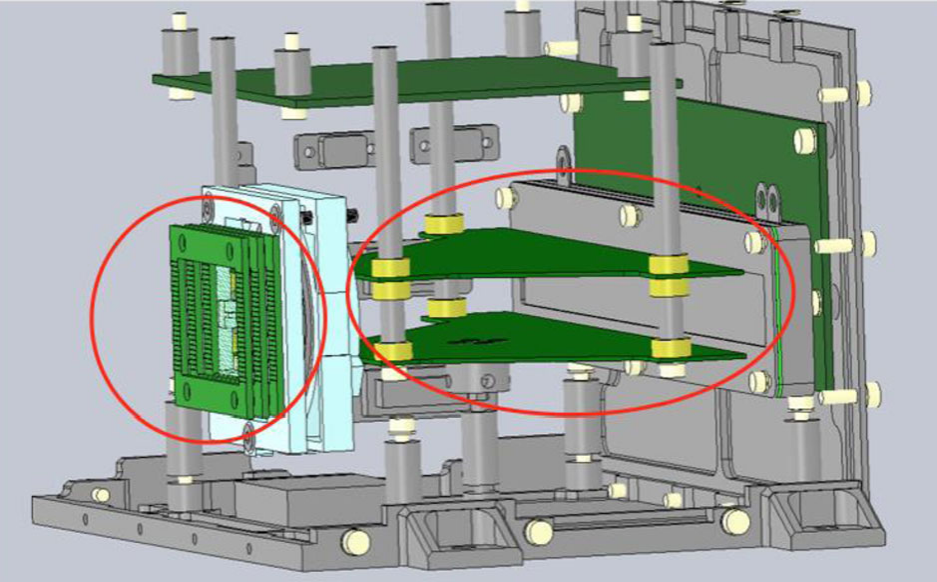
ELENA internal design. The red-circles highlight the external and internal charge particle-deflectors

The ELENA PFM is shown in Fig. 44, and its internal design is schematically shown in Fig. 45.

The ELENA unit have been fully calibrated with neutral atoms beam at the MEFISTO calibration facility (Messkammer für Flugzeitinstrumente und time-offlight, University of Bern, Switzerland). ELENA subsystems (ion external and internal deflectors, UV filter transparency and MCP efficiency) have been previously tested at the ENA-Lab facility at IAPS-INAF, Rome.

The performances have been evaluated measuring the following items:

- Dark counts
- Ion rejection capability
- Angular resolution
- Absolute calibration for neutral particles of different species in the ELENA energy range.
- UV-filter UV transparency

#### Calibration/Performance Results

The ELENA PFM have been calibrated at the MEFISTO facility at Bern University for verifying two peculiar scientific objectives: (1) capability to analyse the neutral particles angular distribution to image the planet surface emission areas and exospheric interaction with SW, (2) angular distributions of the ions, within the FOV to evaluate the SW precipitation by looking the mirrored SW from the close-to-nadir direction allowing to add a useful flow direction out of MIPA FOV. To verify the achievement of these two science objectives the calibration must include also the verification of the noise removal. Eventually the calibration deals with the quantization of dark counts, ion deflection efficiency, UV transparency, angular resolution, counts rate/flux.

ELENA FOV range -45^∘^ to +30^∘^ is reached with 28 sectors (form 6 to 32), thanks to the discrete anodes read-out system behind the MCP detector. The ELENA perpendicular direction corresponds to sector 20.

##### Dark Counts

Dark counts background has been controlled by measuring the counts rate without any beam.

The integrated counts are of the order of 30 counts/s as expected.

##### Ion Deflection Efficiency

The ion rejection efficiency has been verified in both models: PFM (on board of the MPO and FS). At the Mefisto facility in Bern collimated beams of H^+^ at energies: E=1 and 3 keV and O^+^ at 4 keV have been used for verifying the rejection efficiency in the FS and H^+^ at energies: E=1 keV in the PFM. The beam extension measured by a Faraday cup in the case of 3-keV H^+^ was about 7 mm and the intensity 6⋅10^6^ p.cle/cm^2^/s. The two deflectors (external grids and internal plates) are shown in Fig. 45. The test consists in ramping-up the HV system (in several Voltage steps), up to input energy and over. The voltages are generated by a unique HV supply for External (grids) and Internal (plates) deflectors; nominal configuration is HV neg = −1 kV and HV pos = +4.5 kV).

**Fig. 46:**
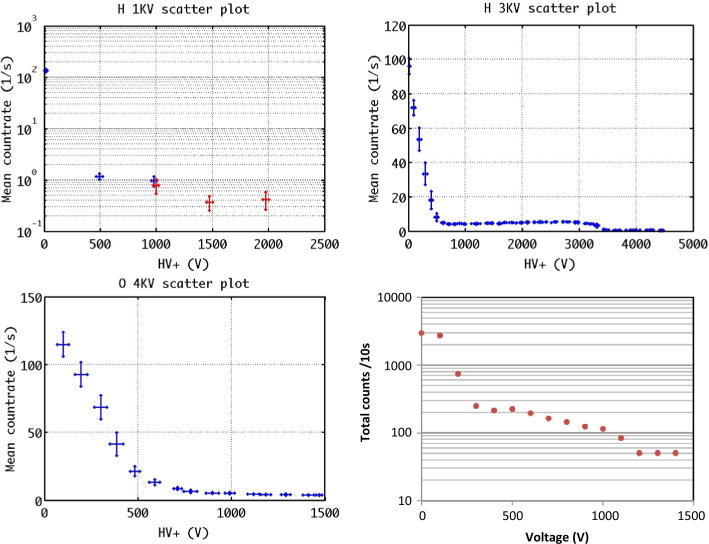
Counts vs PP applied. FS test: 1-keV (*above left*) and 3-keV (*above right*) H^+^ 4-keV O^+^ (*below left*). PFM test. PFM test: 1-keV H^+^ (*below right*)

Test results are shown in Fig. 46. These results show that the bulk of the ions signal is removed by the internal deflector at low PD applied; while, increasing PP above the ion energy, also the neutrals generated by the interaction of ions with the entrance grids are fully removed by external deflectors. Results are summarized in Table 3. The required ion rejection R_ion_ is of the order of 99.5% for protons and 90% for heavy ions (see Sect. 4) so the requirement has been successfully achieved.

**Table 3:** ELENA ion rejection capability for different species/energies

Species	E (keV)	R_ion_ (%) @ *V*_app_<V_th_	R_ion_ (%) @ V>V_th_	R_ion_ (%) @ nominal
H^+^	1,0	97.3-99	98.3-99.8	99.0 ± 0.8
	3,0	96	99.5	99.5
O^+^	4,0	97	NA	97

##### Angular Resolution

**Fig. 47:**
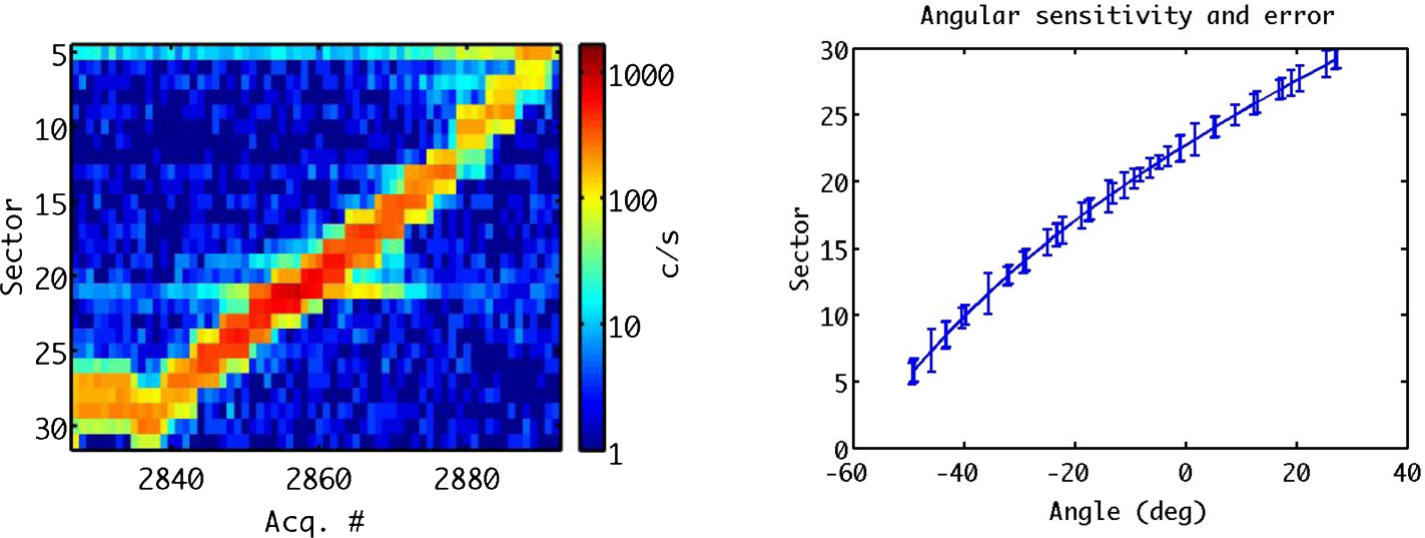
1-keV O angular scan vs acquisition number (*left*), T_int_=10 s. The sector distribution of the scanning as a function of incidence angle (*right*) shows the angle-sector correspondence and spread. Note that the efficiency is lower at higher angle as shown by the normalization factor (below)

Different energies and species and charge states have been tested in the PFM for evaluating the angular resolution and the absolute calibration at the Mefisto facility in Bern. In Fig. 47 an example of angular scan is shown. Note that the anodes are disposed on two rows so that there is an over-sampling of angles, which further improves the angular resolution.

The verified angular resolution is < 5^∘^, in agreement and even better than the science performance requirement.

##### Angular Response

**Fig. 48:**
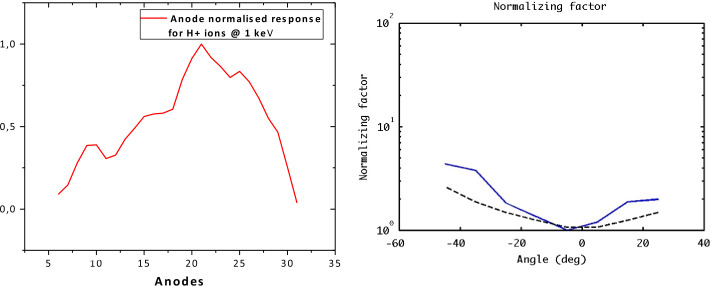
(*left*) Normalized angular scan counts for 1-keV H^+^. (*right*) Observed sector normalization factors for 1-keV O (blue line), and theoretical curve of the normalization factor (dashed black line)

The angular response trend (AR) is as expected according to the input direction (Fig. 48). The minor responsivity at higher angles is due to the geometric shadow of the finite width of the membranes. An extra reduction of responsivity at high entrance angles could be due to MCP efficiency reduction at wide incidence angles. Final AR values for each sector are reported in Table 4.

**Table 4:** ELENA angular response factor (AR) for each sector

Anode	Angular response factor
31	0.03724
30	0.25408
29	0.46559
28	0.55005
27	0.67279
26	0.77142
25	0.83367
24	0.7979
23	0.86344
22	0.91817
21	1
20	0.91206
19	0.78536
18	0.6056
17	0.58136
16	0.57593
15	0.56097
14	0.49015
13	0.42305
12	0.32747
11	0.30613
10	0.38881
9	0.38615
8	0.27913
7	0.14541
6	0.08946

##### Calibration Curve

The expected counts $C_{\mathit{ei}}$ on a single anode are:

$$ C_{\mathit{ei}} =F_{t} {\cdot} r{\cdot} T{\cdot} \mathit{AR}_{i}\ \varepsilon {\cdot} K_{d}{\cdot} K_{M}{\cdot} A $$

where:

$F_{t}$ = total input flux

$r$ = fraction of $F_{t}$ impinging on the single sector, estimated as $C_{mi} /S_{s}$ where $C_{mi}$ are counts on the sector $i $ and $S_{s}$ are total counts for all sectors (sector sum)

$T$ = shutter membrane transparency = 10^−2^

$AR_{i}$ = normalized angular response of each sector

$\varepsilon $($E$) = MCP efficiency (Rispoli et al. 2013)

$K_{d}$ = reduction factor due to electronic readout loss time (1.6 s/Tint→84%, where Tint=10 s)

$K_{M}$ = reduction factor for electronic memory load (93%)

$A$ = area = 1 cm^2^

**Fig. 49:**
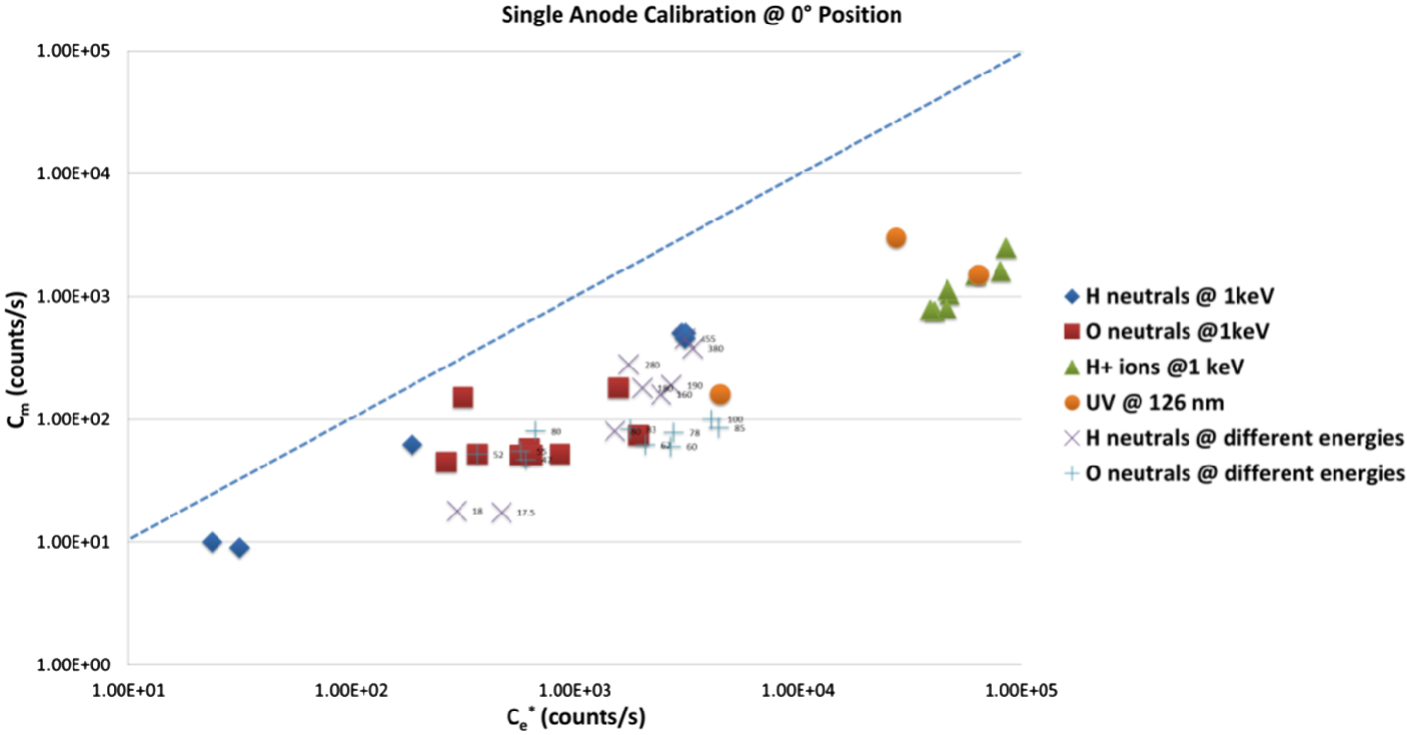
Expected counts at 0^∘^ angle to the normal for different flux intensities, energies, species and charges vs real counts at the peak sector. Comparison with the 1 by 1 curve (dashed line)

Fig. 49 shows the sector integrated counts $C_{m}$ vs $C$_*e*_ @ 0^∘^ in bi-log scale (note that $AR$(0^∘^) = 1). The counts are lower than expected especially at higher count rate probably due to a different rate response of the read-out system composed by MCP detector plus the acquisition chain. The best-fit curve obtained using all the tested particles is: $C_{e} = $ 0.51 ⋅ *C*_*m*_^1.61^. The estimated uncertainty on the $C_{e}$ is in the range 50%-75%.

Same analysis have been performed for the other anodes / incident directions. The calibration fit curve results applicable to all the anodes by applying the angular response.

It is possible to finally obtain the absolute calibration for each sector, by assuming a specific energy and species, that is, a specific MCP efficiency, $\varepsilon (E)$. For $T_{\mathit{int}}{=}10$ s the calibrated flux is:

$$ F_{i} =64.5\cdot {C_{\mathit{mi}}}^{1.61}/\varepsilon (E)/\mathit{AR}_{i} [1/(\mbox{cm}^{2}\,\mbox{s})] $$

##### UV Transparency

At the Mefisto facility in Bern a collimated UV beam (at $\lambda =126$ nm) of intensity estimated as ∼1.5 10^11^ ph/s cm^2^ and extension width about 1 cm^2^ has been used for measuring the Transparency of ELENA to UV at different incidence angles.

**Fig. 50:**
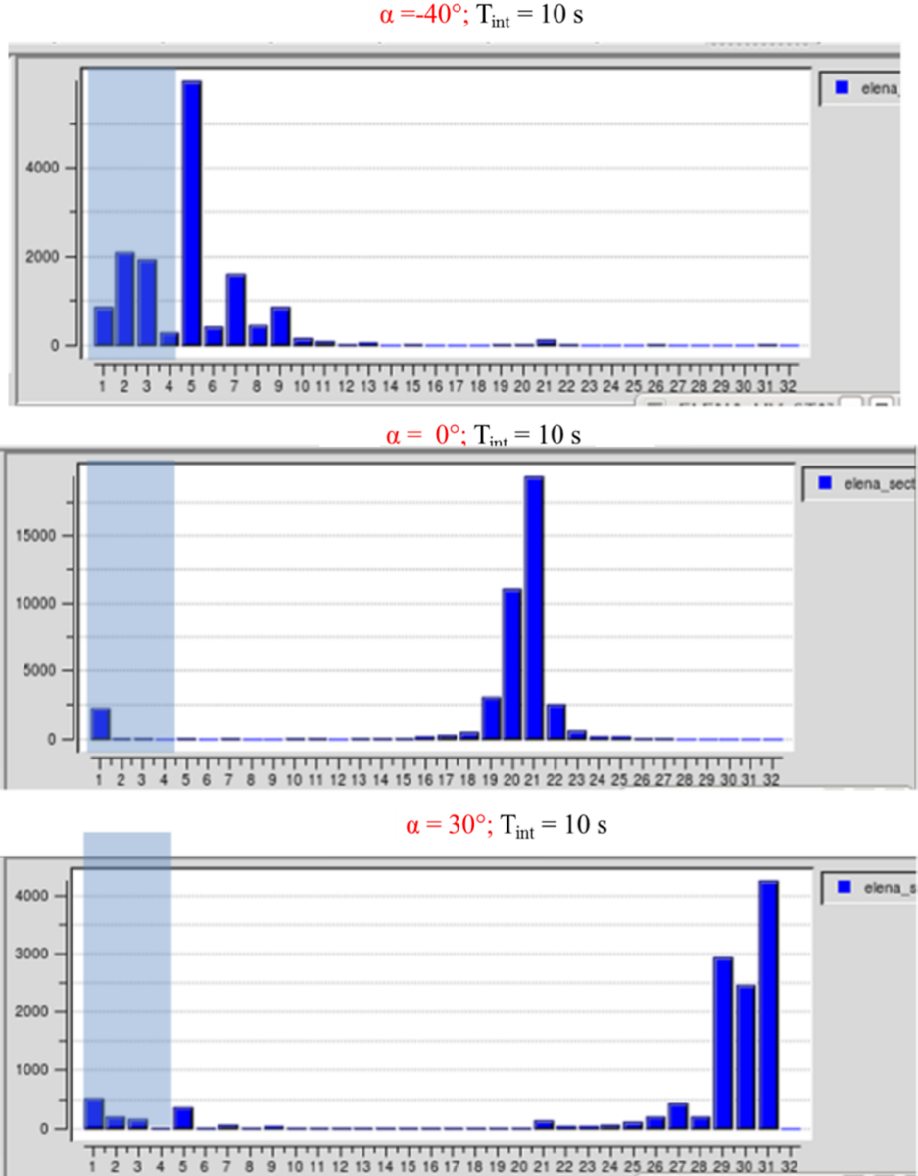
Counts/s of UV at ELENA when a source of intensity 1.5 10^11^ ph/(cm^2^ s) at -40^∘^ (above), 0^∘^ (middle) and 30^∘^ (below) has been used, T_int_=10 s

The ELENA measured counts at integration time 10 s are shown in the histograms in Fig. 50. Note that the anodes are disposed on two rows so that small beam impinging in one raw is shown as a saw tooth shape.

The measured ELENA grids transparency to UV, considering all sectors integrated counts and peak integrated counts could be evaluated as

$$ T_{\mathit{UV}} = \Sigma (C_{\mathit{ei}}/\mathit{AR}_{i}/F/\eta \square/A) $$

Where:

$T_{UV}$ = UV transmittance of the membranes

$\eta $ = efficiency of the system = $\varepsilon $($E$) $K_{d}\ K_{M}$

$F$ = Photon flux.

$C_{ei} = $ 0.51 ⋅ *C*$_{mi}$^1.61^=Expected counts calibrated according to correction function at 0^∘^

$\mathit{AR}_{i}$ = normalized angular response of each sector described

$\varepsilon $(*Ly*$\alpha $)=MCP efficiency = 0.02.

Average $T_{\textit{UV}}$ between -25^∘^ and +25^∘^ is 5.5 10^−5^ considering the total sector integrated counts, in good agreement with the estimated $T_{\textit{UV}}= 3\ 10^{-5}$ (see Sect. 4). The estimated uncertainty of this parameter is between 50%-75%.

#### Calibration Summary

The ELENA PFM unit has been full calibrated with neutral atoms beam at the MEFISTO calibration facility of University of Bern. The actual performances are summarized in Table 5.

**Table 5:** Summary of ELENA performances

Parameter	Required	Actual	Comments
Energy range	>0.1 – 5 keV	>0.100 – 5 keV	
Viewing angle	5^∘^×70^∘^	4.5^∘^×76^∘^	
Angular resolution	5^∘^×8^∘^	4.5^∘^×4.5^∘^	
Optimal temporal resolution	<15 s	>=5 s	
Efficiency		30% for 500 eV	Energy dependent
Pixel Geometric factor	≥ 10^−5^ cm^2^ sr	1 10^−6^ - 2 10^−5^ cm^2^ sr	Counts rate dependent
Integral Geometric factor	≥ 10^−3^ cm^2^ sr	3 10^−5^ - 6 10^−4^ cm^2^ sr	Counts rate dependent
UV transparency T_UV_	< 3 10^−5^	5 ± 3 10^−5^	
Ion rejection R_ion_ (1 keV)	< 99.5%	<99.0 ± 0.8%	

### Strofio Calibration and Test

**Fig. 51:**
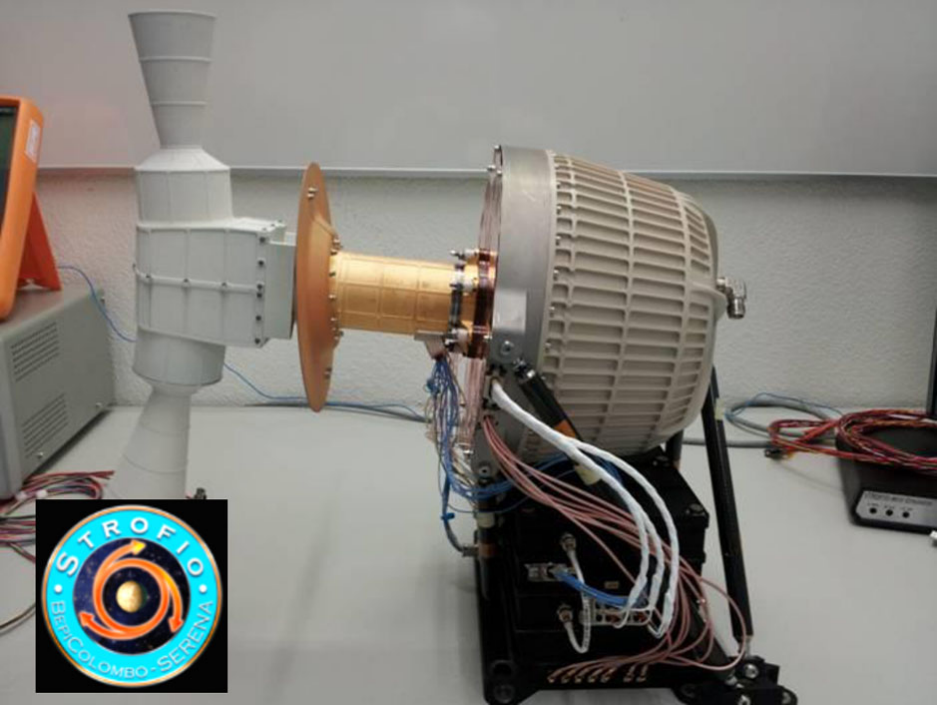
Strofio Flight Model/FM)

The Strofio FM is shown in Fig. 51.

The ionizing source has been developed and calibrated using the MEFISTO facility at the University of Bern (see De Angelis et al. (2011)). Objects of the source calibration are:

- Characterize the efficiency of ionisation for different ion species
- Optimize the field of view and focusing properties of the source
- Verify operations at different temperatures

MCP detector has been characterized as a “stand alone” system at the Applied Physics Laboratory. Gain uniformity and gain variation with applied voltage has been measured and documented, using laboratory equipment.

The ion optics has been developed and calibrated using laboratory electronics at the ion mass-spectrometry facility at the Southwest Research Institute. The assembled ionizing source, ion optic, MCP, and flight electronics has been tested at the neutral mass-spectrometer facility at SwRI, after integration and prior to the environmental tests.

Final calibration of the compete instrument has been performed at the CASYMIR facility at the University of Bern

#### Calibration Metric Test

There are no differences between the current system that has been calibrated and the expected operational system schematized in Fig. 20.

Several parameters were determined and optimized during calibration activities. These include:

1. 2 angles: elevation and azimuth Field of View (FOV)
2. Energy (velocity) filter
3. Neutral/ion
4. 30 optimized voltages

Table 6 shows the key measurements that were verified during the Strofio calibration efforts.

**Table 6:** Key measurements and parameters obtained during Strofio calibration

Parameter	Value	ERD Reference
Voltages	30	
Sensitivity	>0.1 (counts/s)/(particles/cm^3^) for H_2_O (corresponds to 0.1A/torr ion yield and 6% transmission)	ERD_1049
Mass Resolution	m/Δm ≥ 60	ERD_1035
Mass Range	H (1 amu) through Fe (56 amu)	ERD_1028, ERD_1030
FOV	±10^∘^	ERD_1061
KOZ	±20^∘^	ERD_1059
Velocity Filter	Energy range: 0-10 eV	ERD_1784, ERD_1785

#### Calibration/Performance Results

##### Voltage Optimization

Voltages were optimized for best resolution and maximum sensitivity for Strofio when operating the primary or the secondary source.

##### Efficiency

**Fig. 52:**
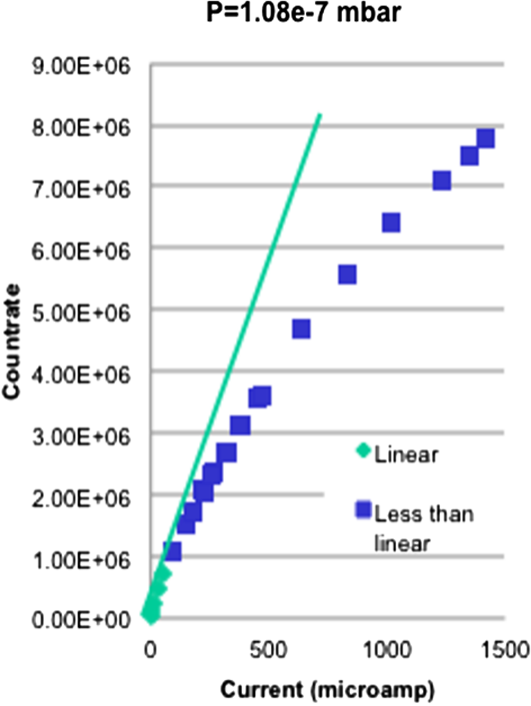
Relationship between count rate and ionization current at a pressure of 1.08×10^−7^ mbar

**Fig. 53:**
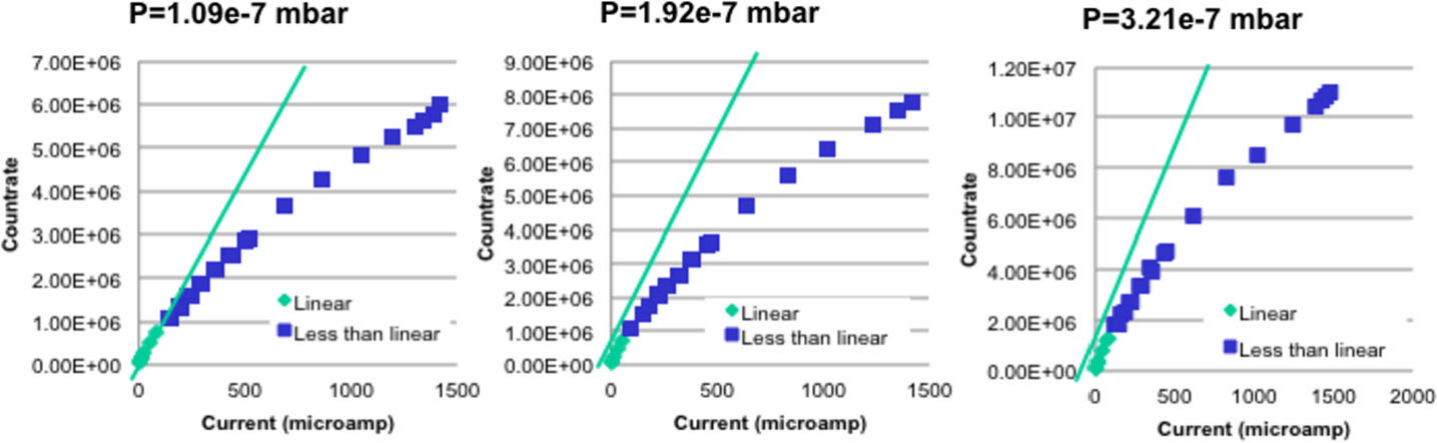
Multiple pressure scans

Figure 52 shows a relationship between count rate vs. the ionization current at constant pressure as the ionization current changes from 0.001 to 1.4 mA. Below 0.08 mA the relationship is linear (as predicted by theory), above 0.08 mA, an increase in current produces an increase in the count rate, but this is significantly less than that predicted by the linear theory. This is due to space charge density of the ionizing electrons (Gauss’s law) that creates a local potential in the ionizing region. This potential tends to “trap” the ions in the ionizing region, effectively reducing the sensitivity of the sensor. Figures 52 and 53 show the relationship between the count-rate vs. current at different constant pressures.

If the effect of space charging was negligible, we would expect to find;

$$ \mathit{Counts}=k*P*I $$

Where:

$P $ = pressure

$I $ = current.

Space charge reduces the number of counts as:

$$ \mathit{Counts}=k* \left ( P*I \right )^{\alpha }, $$

with $\alpha <1$.

**Fig. 54:**
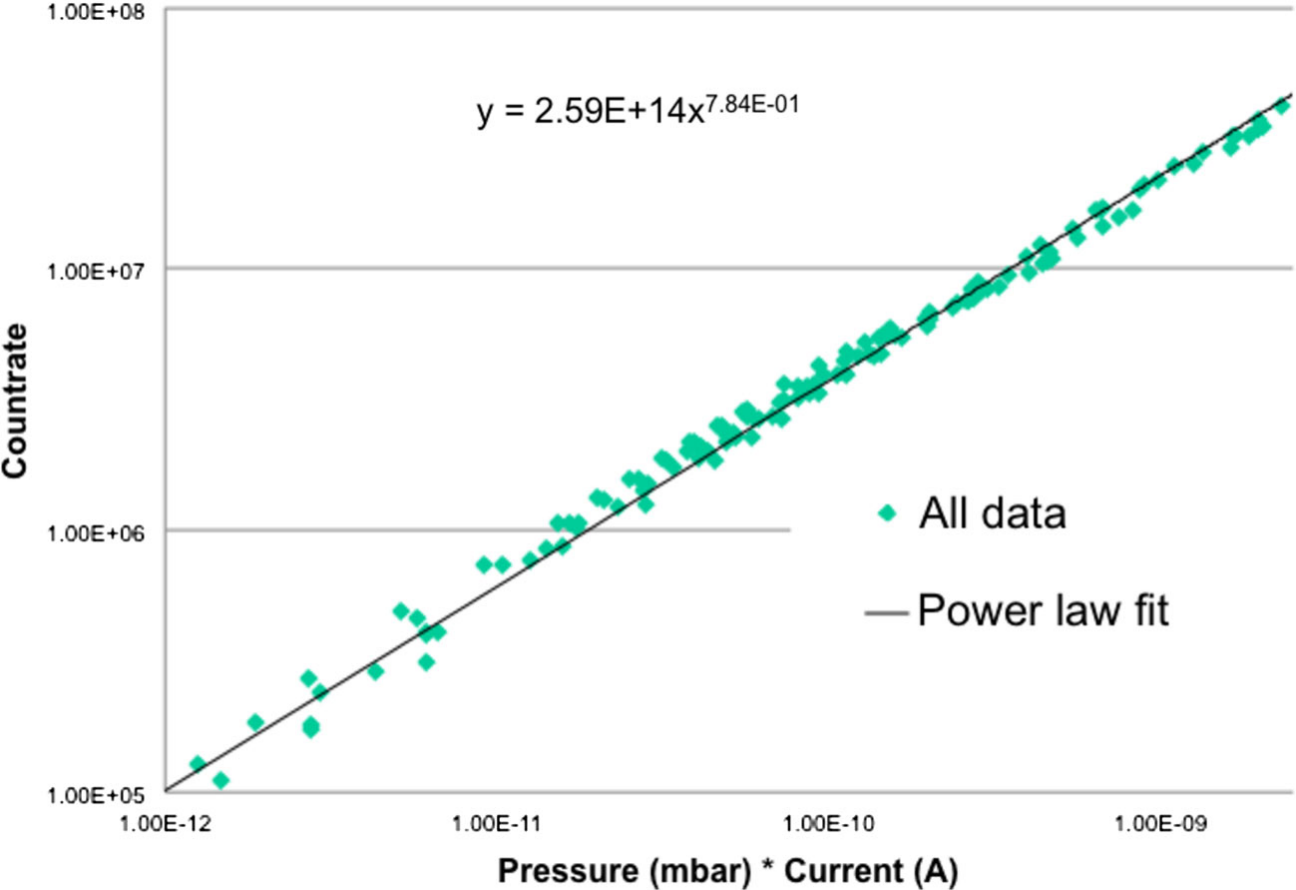
Relation between countrate vs Pressure × Current

Figure 54 shows the results of all scans combined, and that the relation between the count-rate vs. Pressure (mbar) × Current (A) can be approximated by a power-law of the form:

$$ \mathit{Counts}=2.59\times 10^{14} \times (P*I)^{0.784}. $$

Based on these results and the empirical power-law shown in Fig. 54, we expect the count rates at Mercury listed in Table 7.

**Table 7:** Expected count rates at Mercury under different pressures and constant current

n (1/cm^3^)	P (mbar) (600 K)	I (A)	CR (1/s)
10	8.28E-16	1.00E-03	1.73E+00
100	8.28E-15	1.00E-03	1.05E+01
1000	8.28E-14	1.00E-03	6.38E+01
10000	8.28E-13	1.00E-03	3.88E+02
100000	8.28E-12	1.00E-03	2.36E+03

These results indicate that Strofio has a slightly better sensitivity than the baseline value of 0.14 counts/sec/1 cm^3^ at 1 mA, almost a factor of 2 better at maximum emission of 1.5 mA.

##### Mass Resolution and Range

**Fig. 55:**
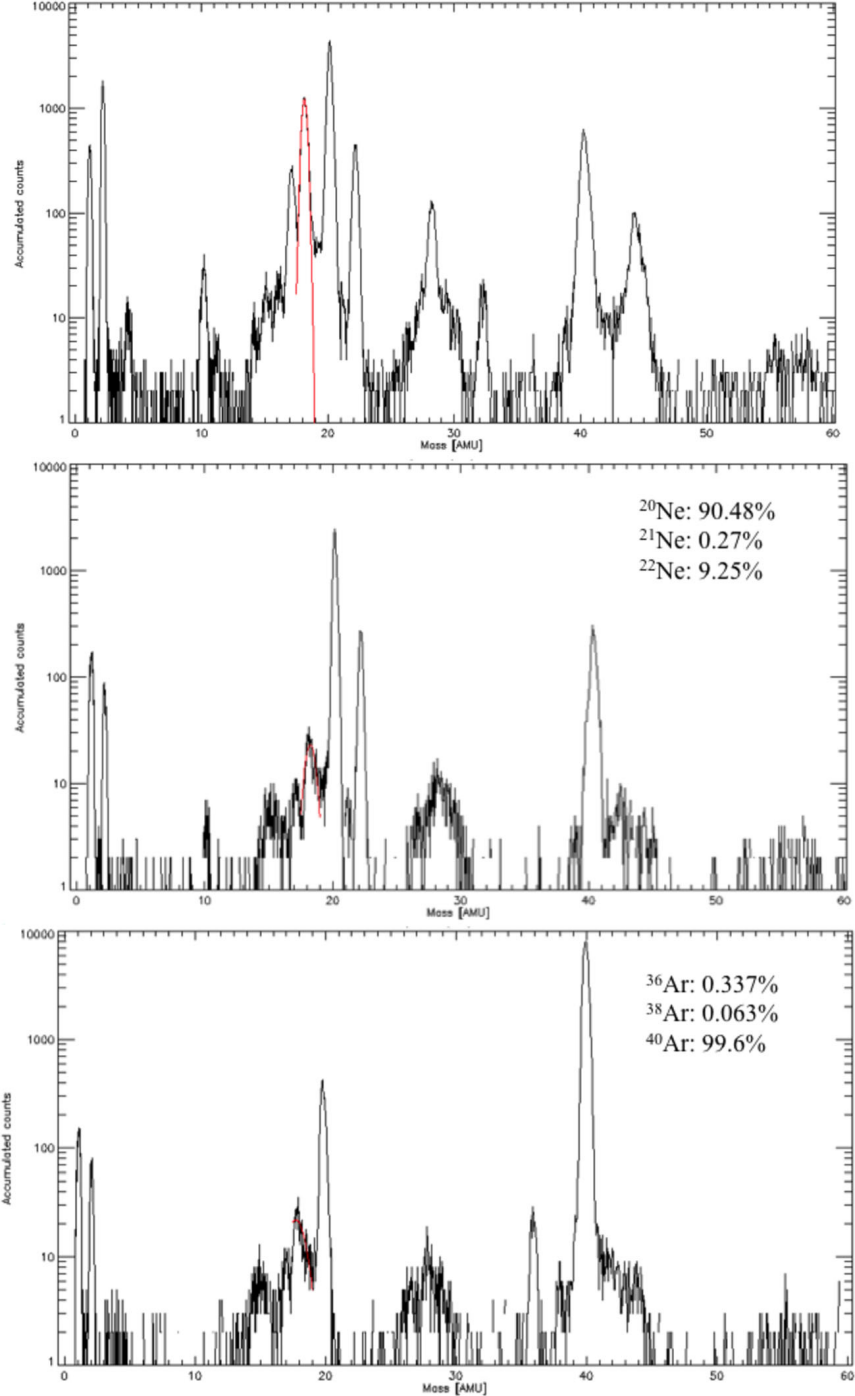
Top: Strofio mass resolution without a velocity filter. Middle: mass resolution of Ne isotopes with a velocity filter. Down: mass resolution of Ar isotopes with a velocity filter

**Fig. 56:**
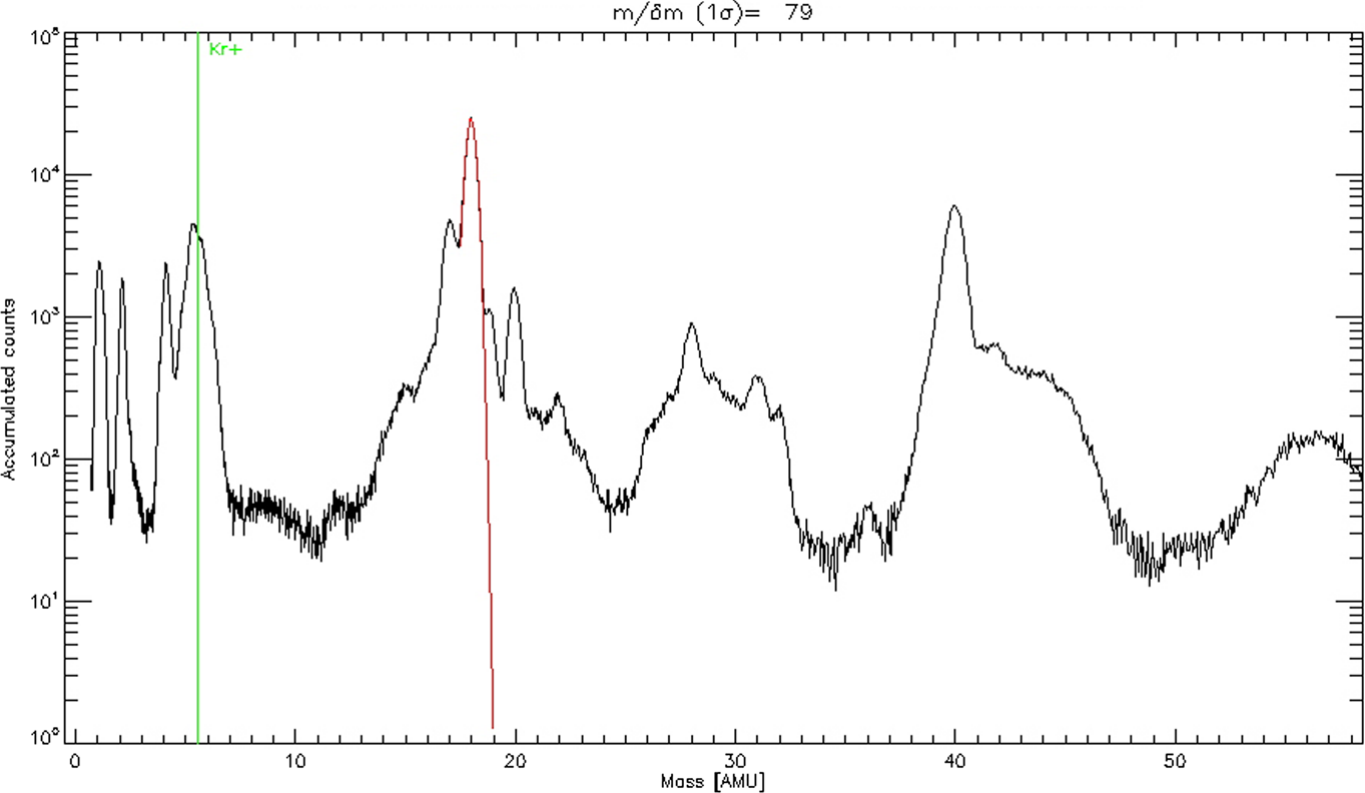
Strofio mass range for two turns

Strofio mass resolution is determined by spatial resolution on the anode and established during voltage optimization. Strofio mass range is determined by the path length in the reflectron and has two inherent ranges corresponding to one turn and two turns. The mass range can be tuned in flight. Figures 55 and 56 show Strofio mass resolution in terms of the accumulated counts versus mass (AMU) for no velocity filter (Fig. 55, top), Ne isotopes with a velocity filter (Fig. 55, middle), Ar isotopes (Fig. 55, down), and the mass range and resolution with two turns (Fig. 56). Figure 56 shows that using two turns, Strofio will detect the heavier species (M>64 AMU), albeit with degraded mass resolution.

Figures 55 and 56 indicate that the nominal mass resolution for Strofio is $\frac{M}{\Delta M} \sim 84$, around m=18.

##### Field-of-View

**Fig. 57:**
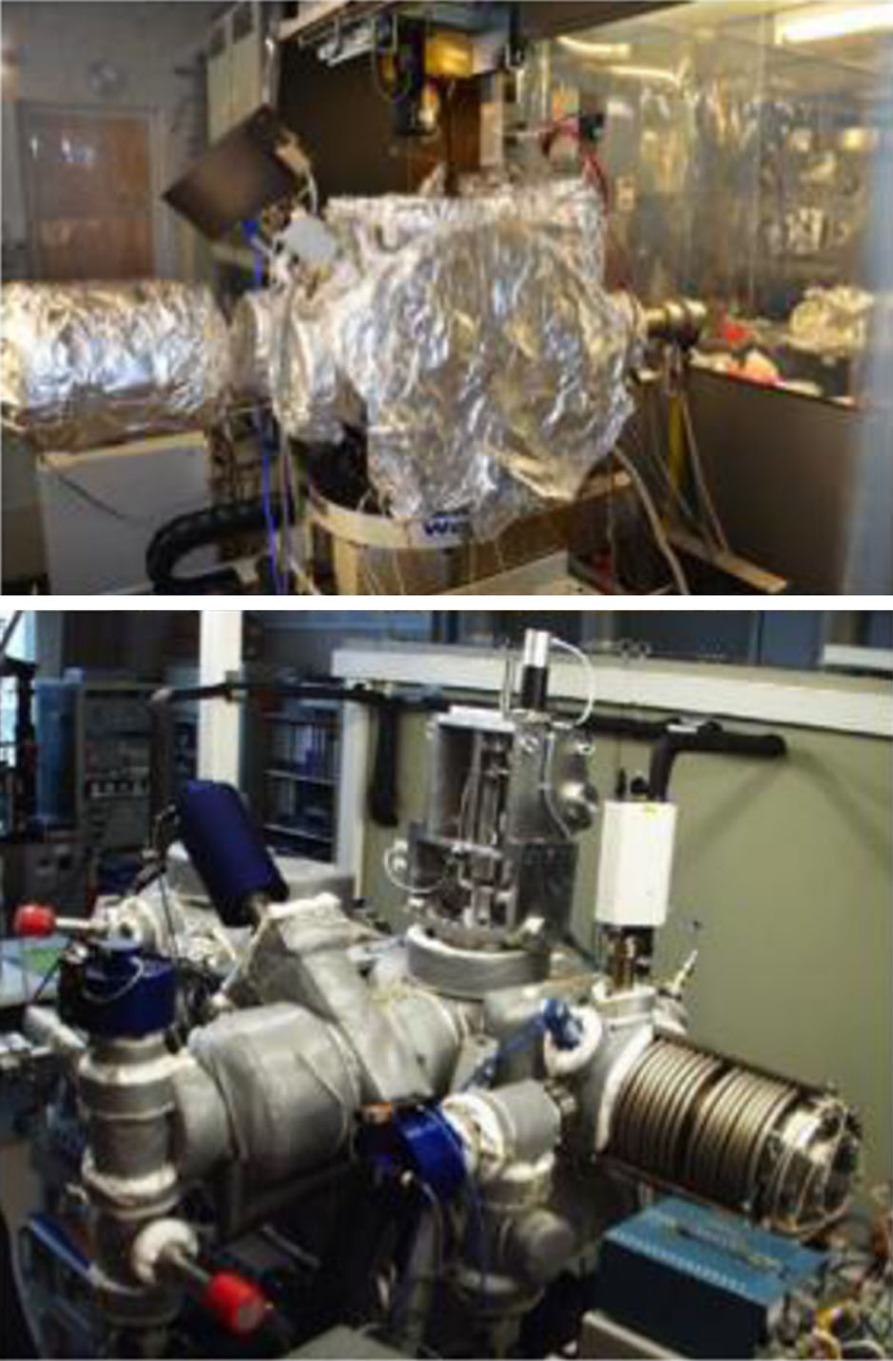
Photo of CASYMIR and the beam facility at the University of Bern calibration facility

Strofio was calibrated at the Calibration System for the Mass Spectrometer Instrument ROSINA (CASYMIR), shown in Fig. 57, at the University of Bern (Westermann et al. 2001; Graf et al. 2004).

The facility has the following key properties:

- Pressure as low as 5×10^−10^ mbar (3.8×10^−10^ torr) to 1×10^−5^ mbar in main chamber
- Particle density range: 10^7^ to 10^11^ cm^−3^
- Pressure absolute measurement accuracy ∼3% below 1×10^−6^ mbar
- Atomic/Molecular neutral beam
- Pure gas absolute beam intensity ∼12%
- Stability ∼7-8% for N_2_
- Beam velocity: 300 to 3000 m/s
- Beam intensity: 10^12^ to 10^15^ cm^−2^ s^−1^

Strofio was calibrated using the following beam properties:

- Pressure ∼10^−9^ mbar with beam
- Species: Ar, Ne, H_2_, He, N_2_, …
- Velocities 1-3 km/s
- Beam size 2-10 mm diameter

CASYMIR had a dedicated chamber specifically for Strofio calibration that was ready for measurements after 2 days with the following characteristics:

- Pressure ∼10^−10^ mbar
- LN_2_ cold plate
- Up to $80\ ^{\circ}$C heating system
- X and Z movement via external table system
- $\varepsilon $ rotation via manual actuator

**Fig. 58:**
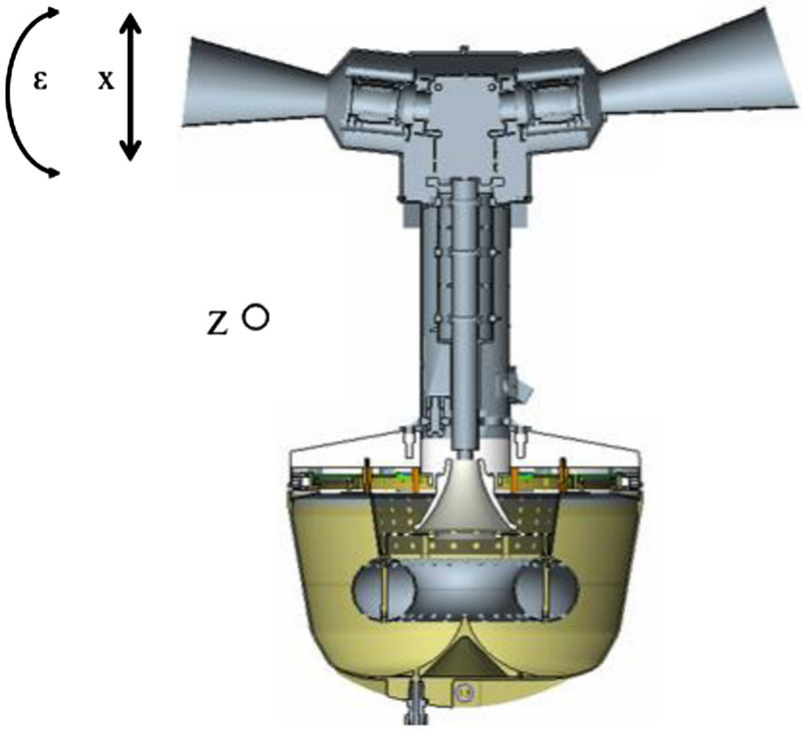
Scan directions with respect to Strofio FOV

The Strofio FOV is determined by mechanically scanning the instrument as shown in Fig. 58. The scan directions are:

- X with table
- $\varepsilon $ with internal mechanism
- Z with table (in-out of the page)

**Fig. 59:**
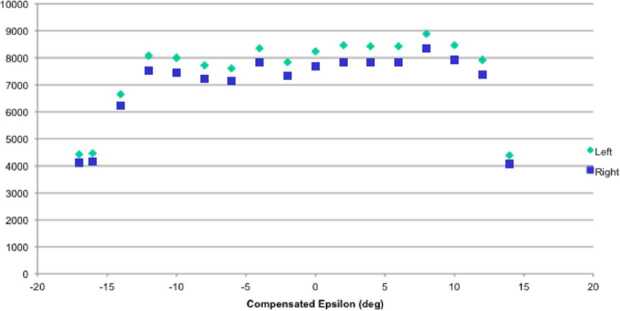
Angular acceptance and response in the $\varepsilon $ direction

Figure 59 shows that the angular response over a FOV of $\pm {12}^{\circ } $ for Ne (without a velocity filter) is relatively uniform.

##### Background Suppression Using a Velocity Filter

Outgassing from the BepiColombo spacecraft will limit the capabilities of the Strofio mass spectrometer to measure small amounts of exospheric gases. Mercury’s exosphere has a total pressure of about 10^−10^ mbar at the surface and <10^−11^ mbar at an altitude of 400 km. Assuming that the composition of background gases from spacecraft outgassing are similar to that observed by the DFMS instrument on the Rosetta spacecraft, we estimated the signal-to-noise ratio for Ar, Ne, and H_2_O that Strofio would be expected to observed at Mercury.

**Fig. 60:**
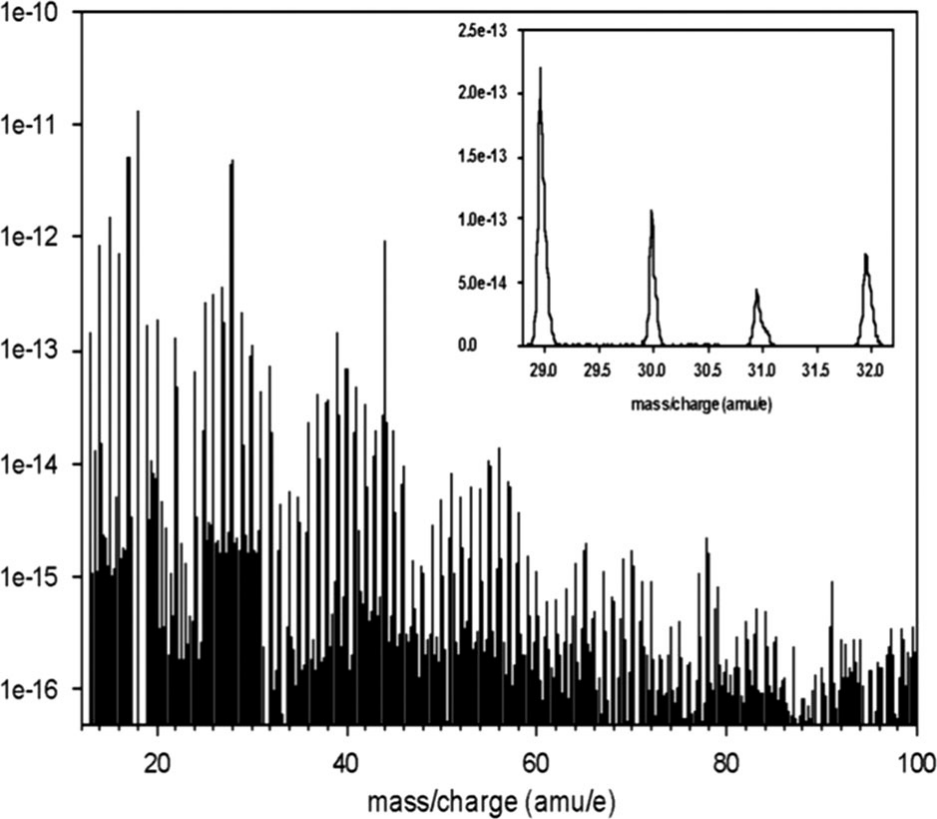
Mass composition of rest gas around Rosetta

Figure 60 shows the mass composition of the rest gas round Rosetta. All mass lines are essentially occupied by fragments of various organic compounds.

Table 8 provides an estimate of the expected densities of various species around Mercury compared with background gases around BepiColombo, extrapolated from the Rosetta in flight data. Blue are species already measured around Mercury, white species estimated to be present, but not measured yet; red highlight species that Strofio will not be able to measure, yellow: species with small S/N ratio; green: species with good S/N ratio. Two cases are presented: without and with background rejection

**Table 8:**
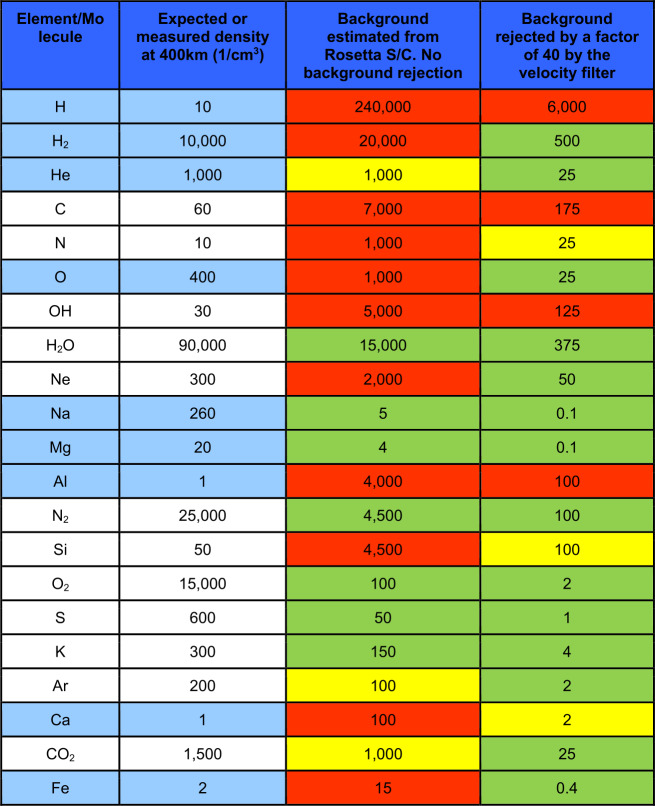
Forecast of Signal around Mercury

To enhance the Signal-to-Noise (S/N) ratio, a velocity filter was implemented in Strofio. This filter discriminates between the gas evaporating from the spacecraft and the exospheric gas evaporation from Mercury by using the velocity of the spacecraft: the exospheric component will have an apparent velocity in the sensor of 2-3 km/s, while the spacecraft gas will be at rest.

**Fig. 61:**
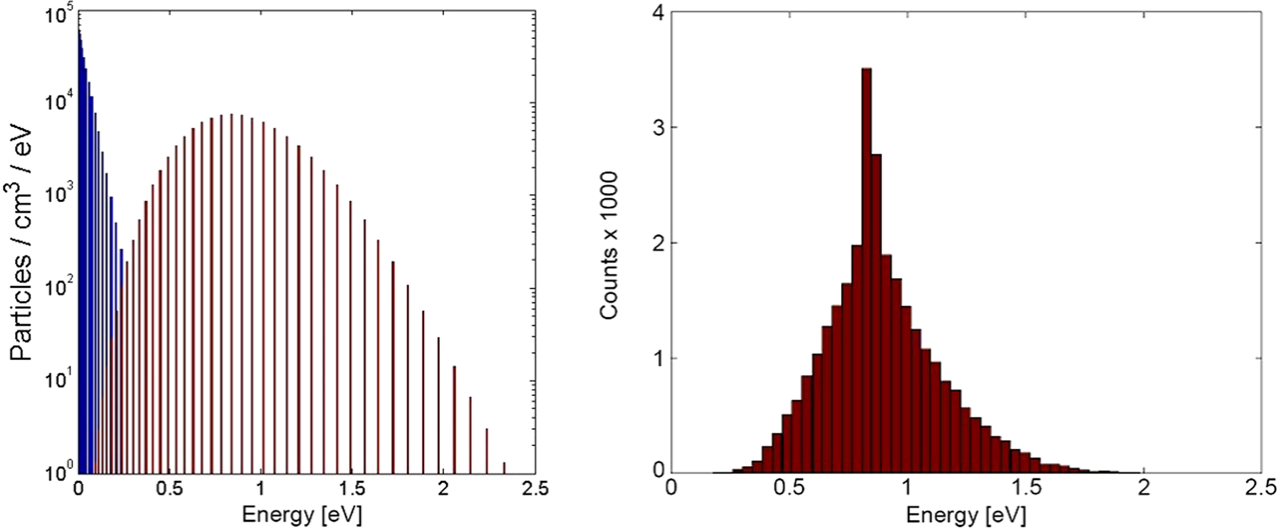
(**a**) Input H_2_O distributions; blue: from spacecraft; red: from Mercury. (**b**) End-to-end transmission using a velocity filter

Figure 61a shows the simulated distribution functions of both neutral components as they are about to be ionized. Blue: from spacecraft, 500 K ∼0.065 eV and Red: from Mercury, 540 K ∼0.069 eV collected with 3 km/s ∼0.85 eV.

Figure 61b shows that the end-to-end transmission using a velocity filter tuned at about ∼0.4 eV. This idealized velocity filter would suppress the H_2_O from the spacecraft by a factor of 40.

**Fig. 62:**
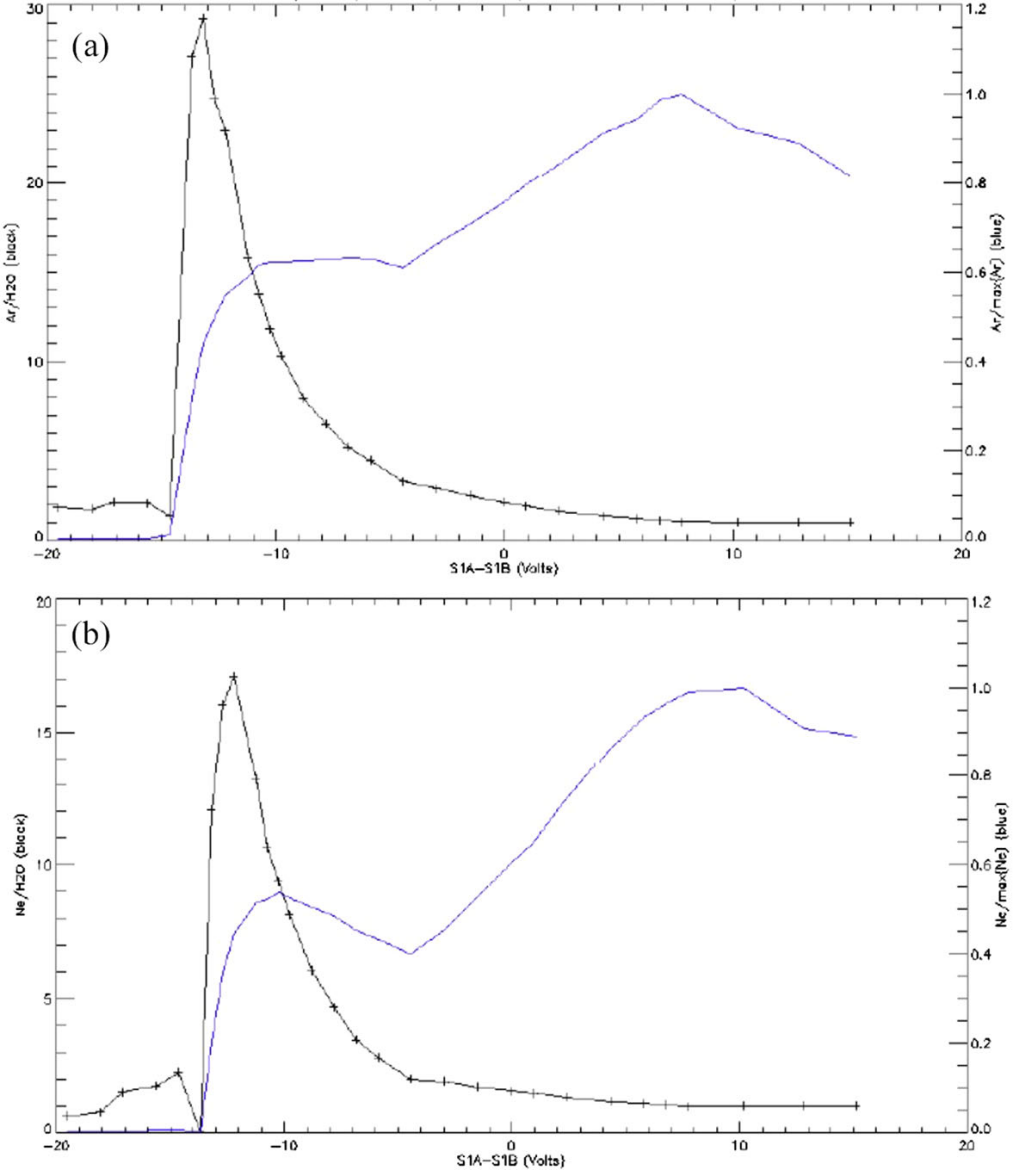
Velocity filter effects on Strofio response for (**a**) Ar and H_2_O, (**b**) Ne and H$_{2}\mbox{O}$

Figure 62 shows the measured performances of the velocity filter. The free parameter is the difference between two voltages in the ionizing source S1A - S1B. The blue curve (scale on the right) shows the total transmission of a beam of Argon (Fig. 62a) and Neon (Fig. 62b) at 3 km/s as a function of S1A-S1B: decreasing this value produces a loss of signal in both Ar and Ne of the order of 50%. As predicted by the simulation, ambient water (always present as rest gas in the chamber) is affected to a much larger extent by S1A-S1B. The black curve (scale on the left) shows the dependence between the original beam/rest gas ratio and the voltage difference. For a setting of S1A-S1B of -10 V, the S/N is increased by a factor of 30 (Ar) and 18 (Ne).

##### UV Contamination Tests

**Fig. 63:**
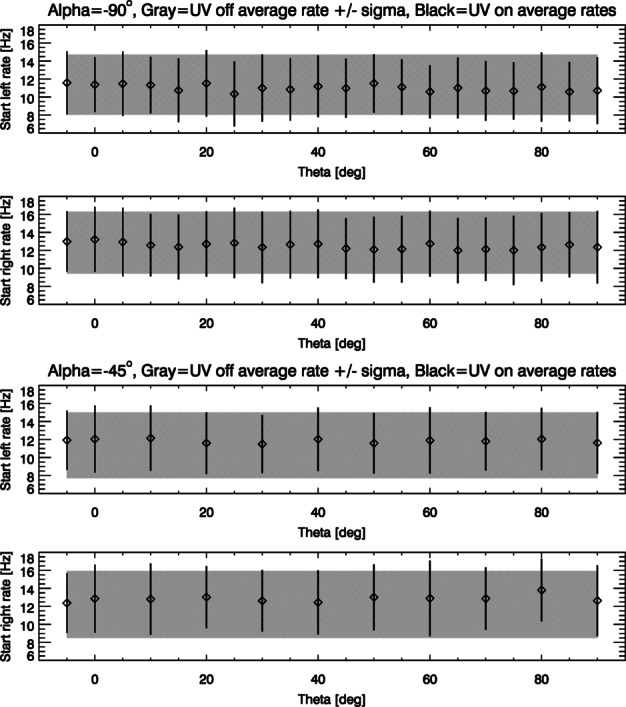
Results of UV contamination tests for Strofio MCPs

UV contamination for Strofio were conducted at Bern by illuminating the sensor with UV intensity corresponding to 1 Sun in Lyman alpha over the whole sphere. Figure 63 shows the results of two particular scans. These indicate that Strofio is not sensitive to UV at the 1 counts/second level.

#### Calibration/Performance Technical Summary

*Calibration facilities:*

University of Bern

Southwest Research Institute

*Production Centre:*

Southwest Research Institute

*Calibration task:*

Several parameters were optimized and determined during calibration activities. These include:

1. 2 angles: elevation and azimuth
2. Energy (velocity)
3. Neutral/ion
4. 30 optimized voltages

##### Calibration Summary

The calibration summary is shown in Table 9. Strofio has been exercised over 1000 hours without malfunctioning. Parameters have been determined to calculate physical parameters (density) from measured quantities (counts) and instrumental parameters (emission current).

**Table 9:** Summary of Strofio performances

Parameter	Required	Actual
Energy range	< eV	0.01-50 eV
Viewing angle [deg]	-	24^∘^ × 24^∘^
Mass resolution *M*/Δ*M*	>50	84
Mass range	-	1-64 amu
Sensitivity	> 10^−1^ (counts/s)/(particles/cm^3^)	0.14 (counts/s)/(particles/cm^3^)
Temporal resolution	< 15 m	10 s

All special mode of operation (i.e. velocity filter) have been tested and characterized. The relevant voltage settings have been determined, as well as the sensitivity to variation of said voltages.

Background rejection capability for UV and Spacecraft chemical Background have been characterized.

In summary: Strofio has the appropriate sensitivity, Field of View, mass resolution, and mass range to measure the chemical composition of the exosphere of Mercury.

### MIPA Calibration and Test

#### MIPA Calibrations Overview

**Fig. 64:**
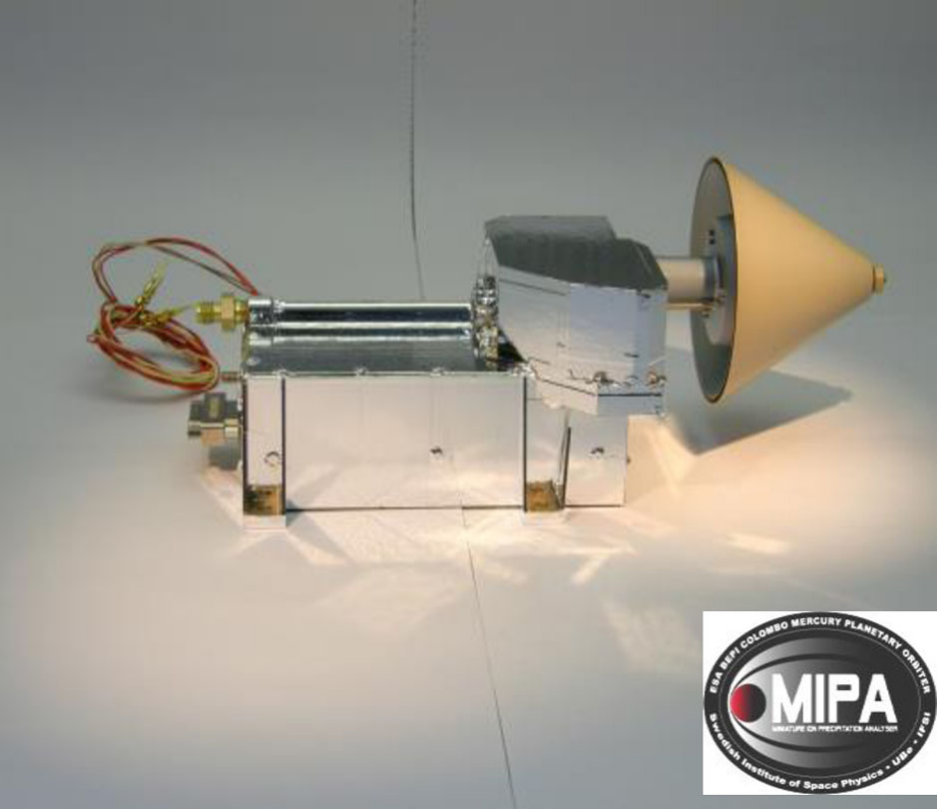
MIPA Flight model

The MIPA FM is shown in Fig. 64.

The instrument was calibrated at the Swedish Institute of Space Physics, Kiruna. For calibration plan see BC-SRN-IF-00030 (De Angelis et al. 2011). For AIV activities see BC-SRN-DS-40001 (Svensson and Barabash 2008).

Solar balance test of the MIPA deflector system was performed at University of Bern.

**Fig. 65:**
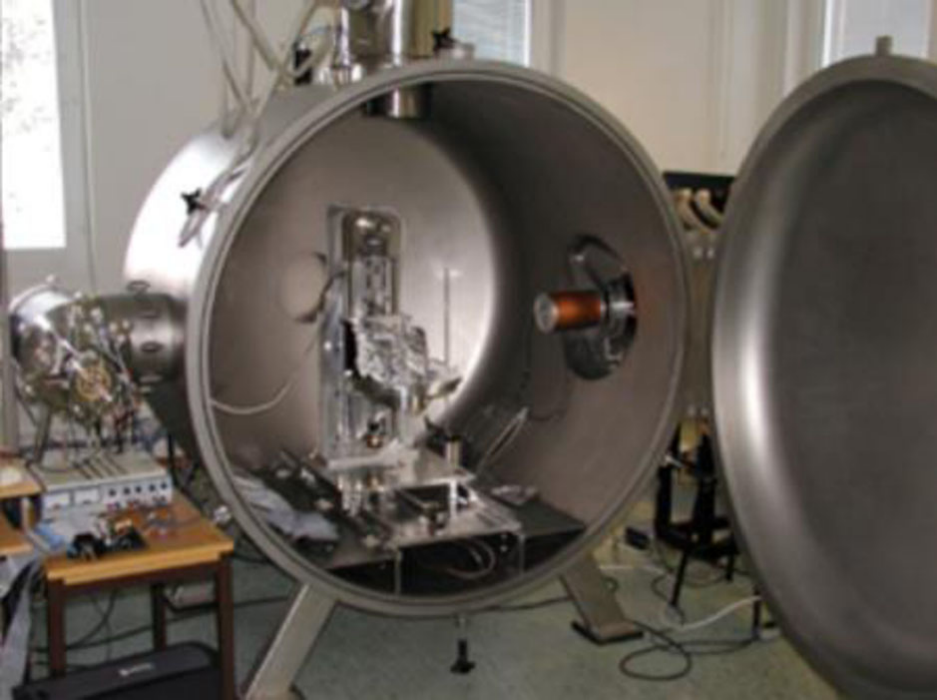
IRF-Kiruna calibration facility

MIPA FM calibrations were performed at the IRF calibration facilities (Fig. 65) in March – June 2013 according to the MIPA Calibration plan (BC-SRN-PR-40000) and reported in the MIPA FM Calibration Report (BC-SRN-TN-40020). The calibration facility generates large diameter (10 cm) parallel beams of selectable ions in the energy range 100 eV – 50 keV. The bean intensity is measured with the Faraday cup. For angular response calibrations the system is equipped with 4-degree turn table.

First MIPA detectors (CCEM) are characterized, then energy and mass resolution, and angular response for each pixel are calibrated. Based on these measurements geometrical factors for each pixel are established.

#### Detector Calibration

**Fig. 66:**
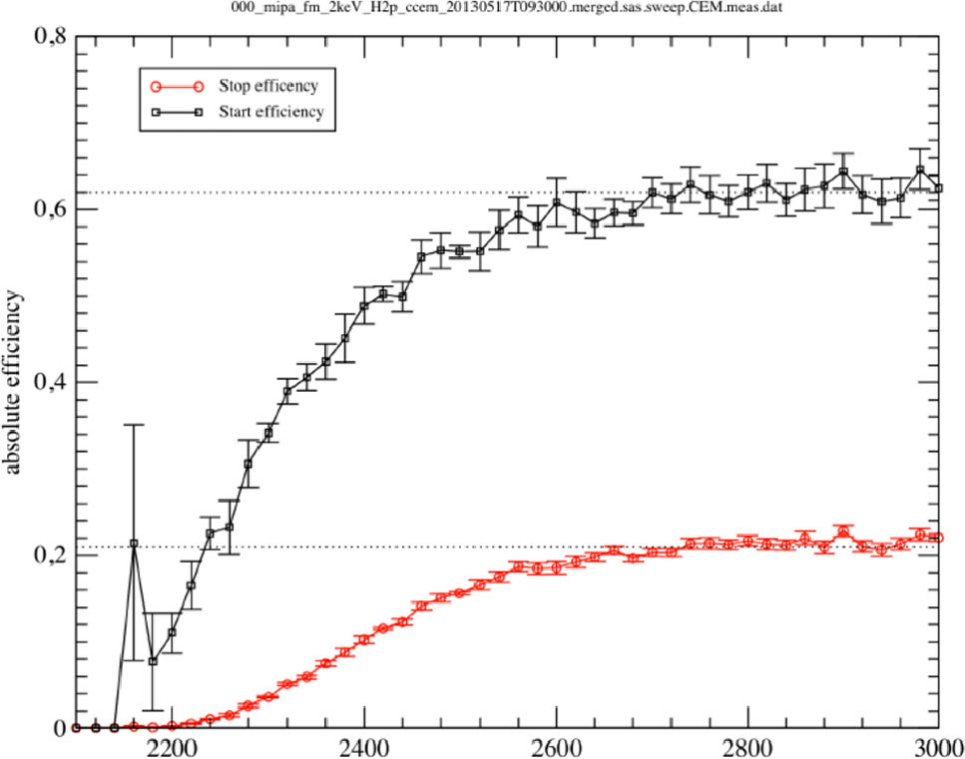
Detector efficiencies as a function of the applied voltages

The detector efficiencies as a function of the applied voltages are shown in Fig. 66.

**Fig. 67:**
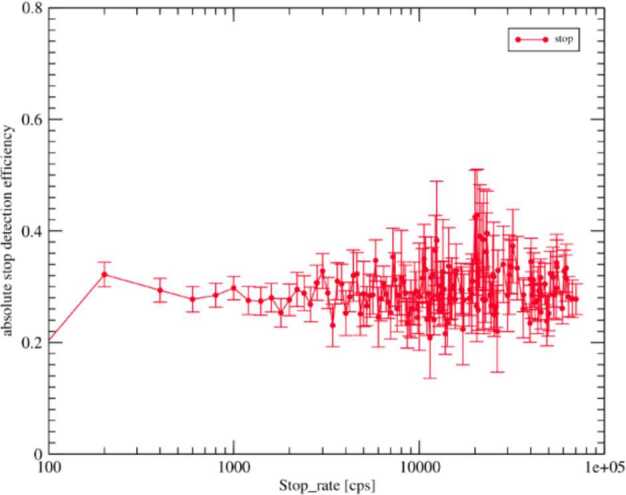
Dependence of the STOP efficiency on the count rate

The operation CCEM voltage is 2600 V. The achieved efficiencies for STOP and START channels are 0.6 and 0.2 as expected. Also, the detector dynamical range was calibrated to establish that the efficiency does not depend on the count rate (Fig. 67). No variations in the STOP count rate up to 8⋅10^4^ cps are visible. The maximum start rate with less than 15% dead time correction on coincidence events achieved was 2⋅10^5^ cps while the noise floor is < 0.001 cps for START. The noise floor for correlated counts is even less, the measurements only allow to establish the upper limit). The achieved dynamical range is > 2⋅10^7^ (only lower limit was established).

#### Instrument Calibration

##### Energy and Mass Coverage and Resolution

**Fig. 68:**
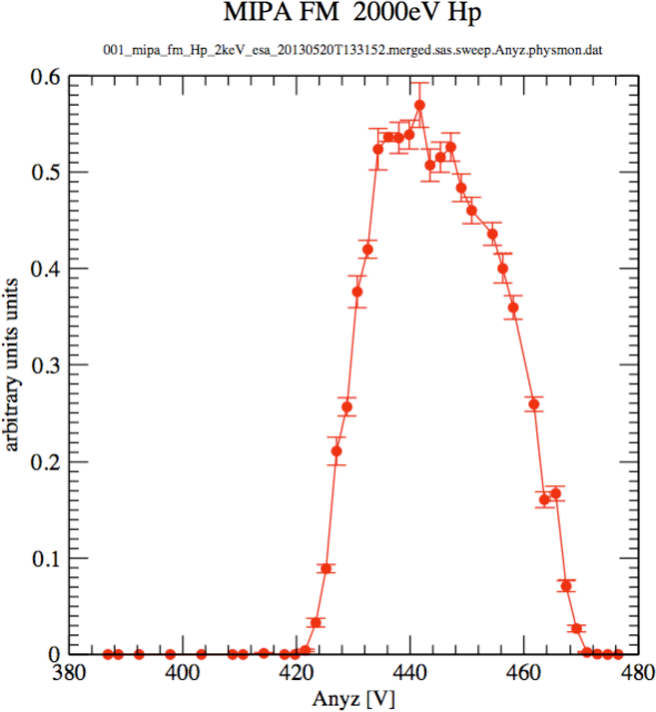
MIPA ESA energy response

**Fig. 69:**
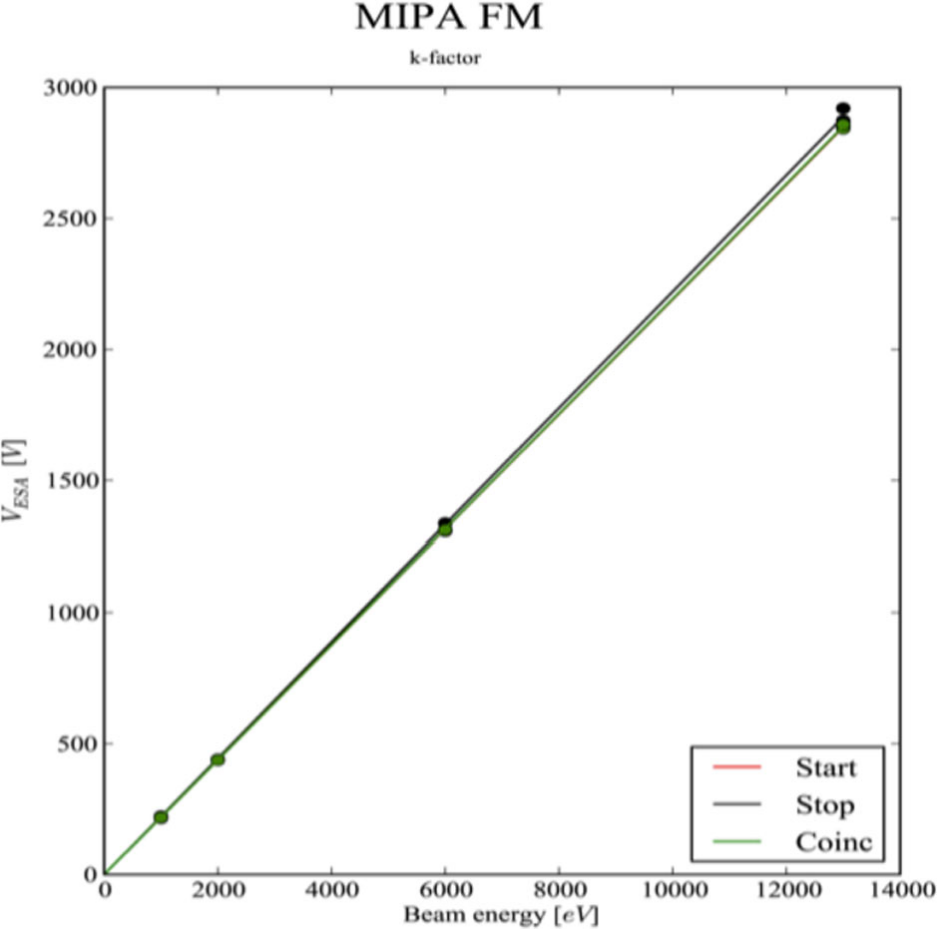
The ESA voltage as a function of the beam energy to establish the analyser constant

Typical ESA response (dependence of the instrument response on the analyser voltage for a fixed incoming ion bean energy) is shown in Fig. 68. The analyser constant is 4.52 (Fig. 69) and the energy resolution 7.2%.

**Fig. 70:**
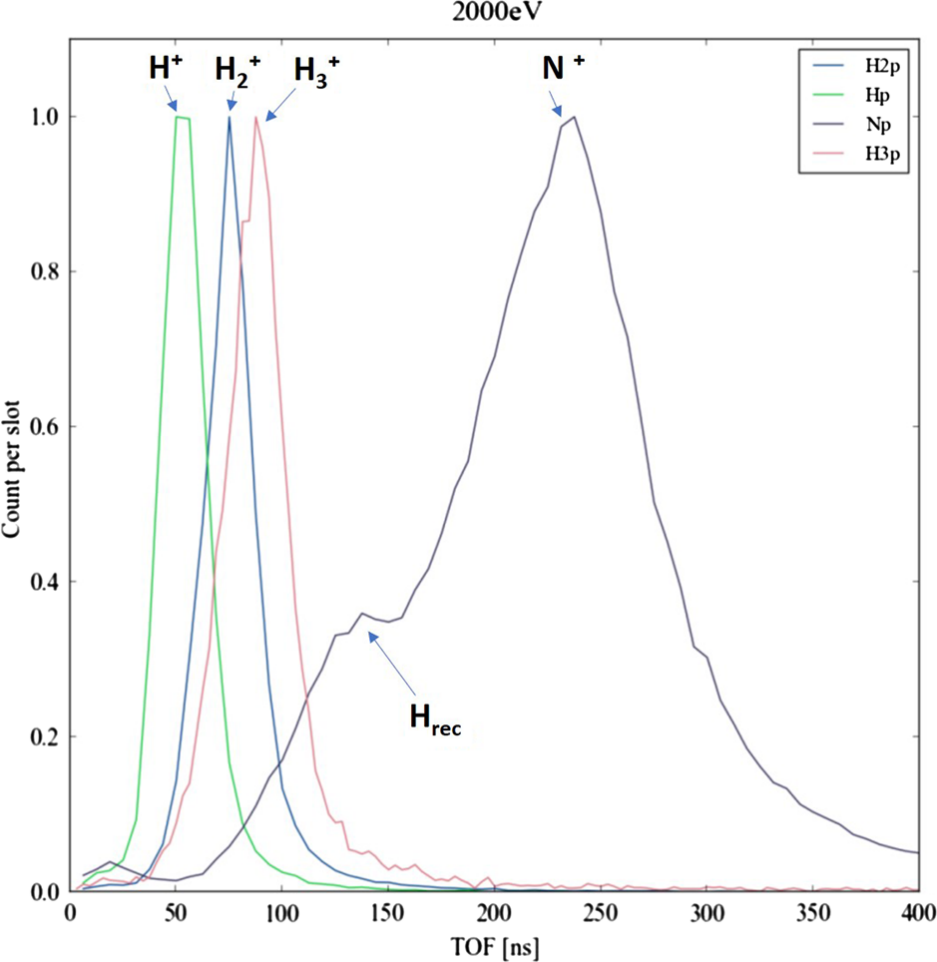
TOF spectrum for ions with M/q = 1, 2, 3, 14

The TOF spectra for different ion species are shown in Fig. 70. The measured mass resolution 2 – 5 depending on mass.

##### Angular Coverage and Resolution

**Fig. 71:**
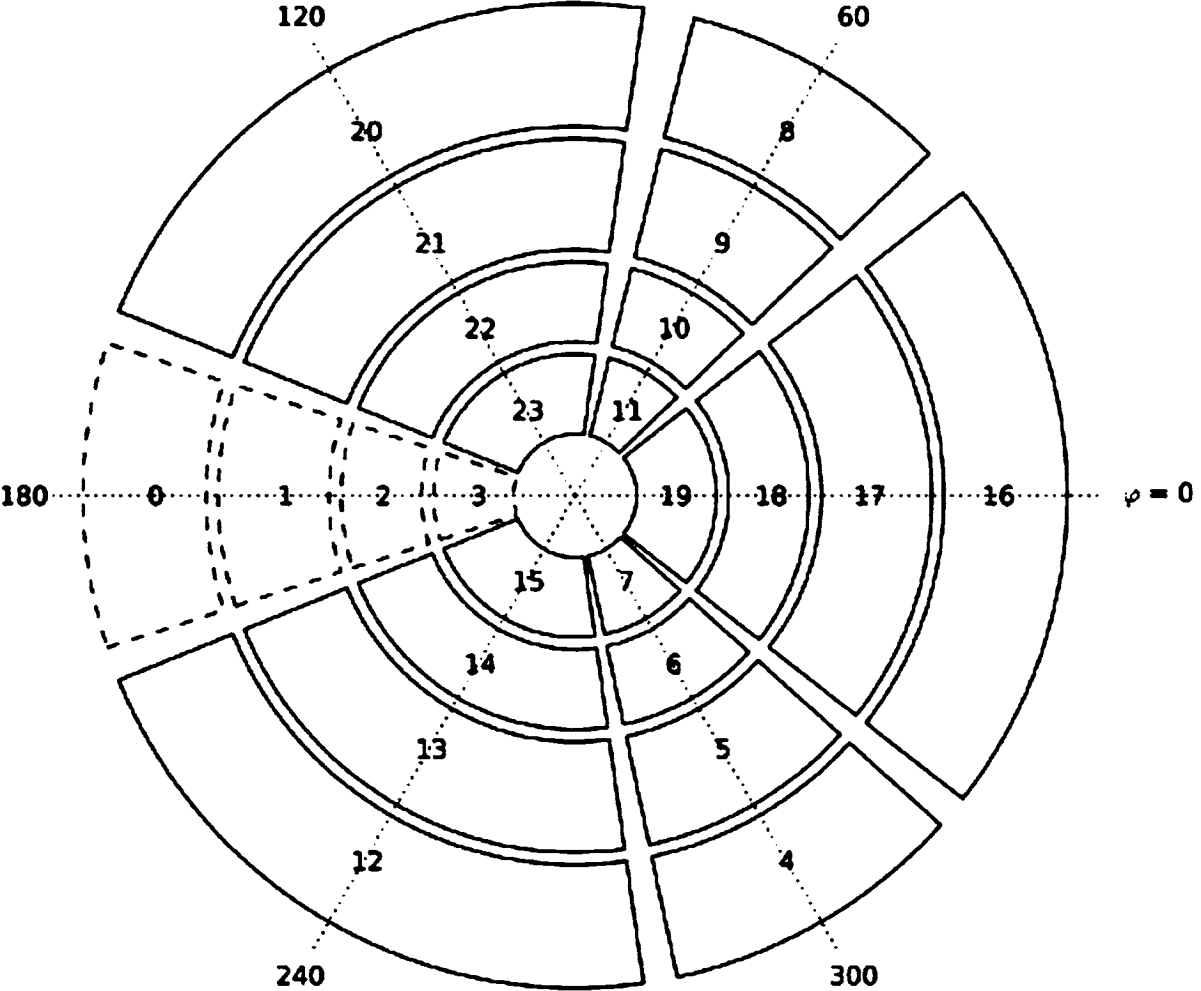
MIPA angular sectors scheme. Note that the Zenith direction is D0 = $180^{\circ}{\times}90^{\circ}$ with $\Delta \alpha {=}30^{\circ}{\times}20^{\circ}$)

In MIPA, 24 (48) directional pixels are available, following the calibrated response for each pixel, they were distributed over 2$\pi $ MIPA FOV with the exception of 8^∘^ azimuth × 90^∘^ polar blind sector in the zenith direction in correspondence to the magnetic boom and 13^∘^ × 24^∘^ in the direction of Low gain antenna boom (see BC–SRN–AN-40003 for justification). MIPA sector scheme is shown in Fig. 71.

MIPA has three different general configurations to obtain a pixel anywhere in the angular space:

- Only one plate is on high voltage, when the two others are at 0 V: to obtain the main directions D1, D2, D3, symmetrically aligned with the middle of their plate (pixels from 0 to 11, the narrowest pixels).
- Two plates are on high voltage, the third one is at 0 V: to obtain the ‘secondary’ directions D12, D23, D31, aligned right in between two plates (pixels from 12 to 23, wider pixels).
- There is also the possibility to power two plates with different voltages, the third being at 0 V: to obtain pixels somewhere in between a principal and a secondary direction. (24-31)

**Fig. 72:**
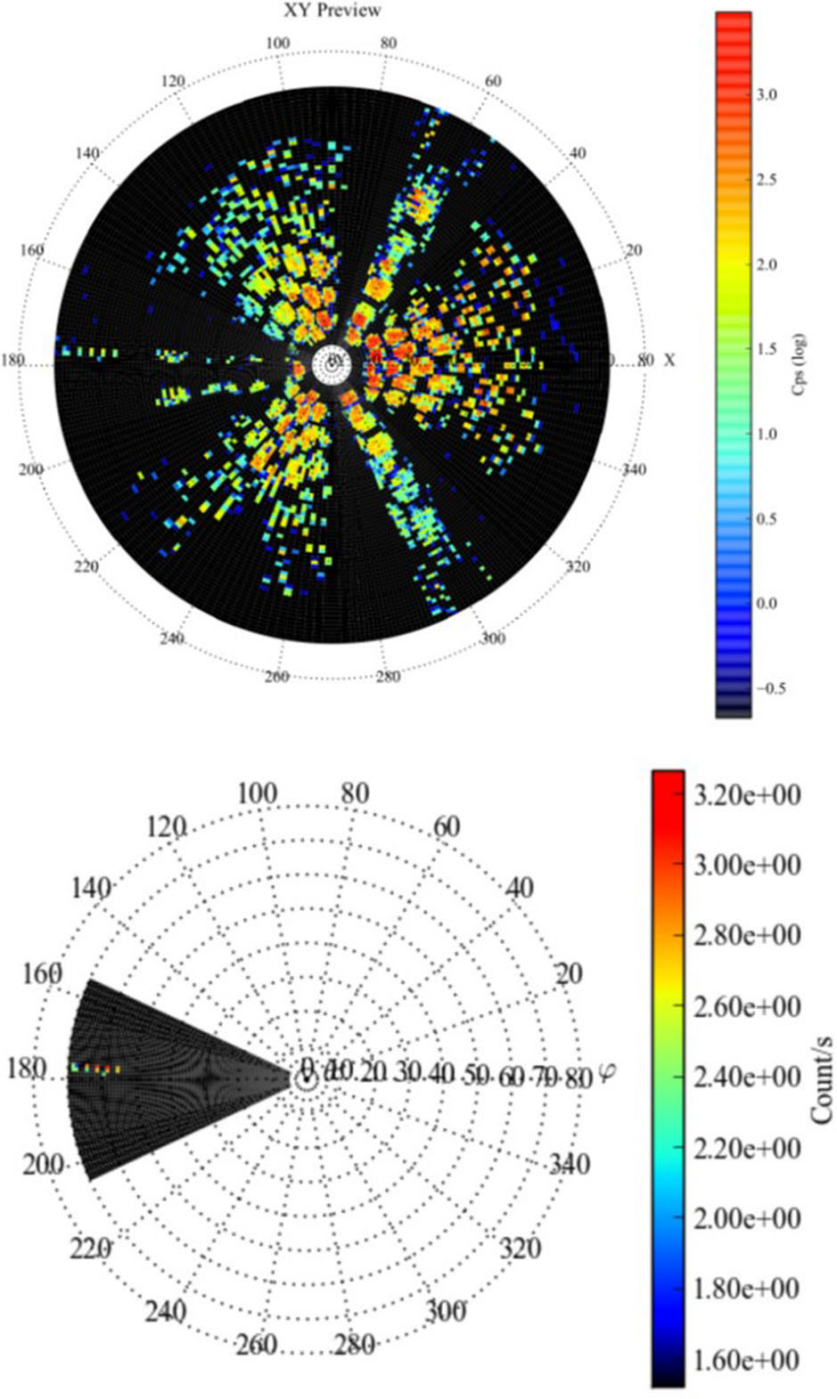
(left) MIPA calibration with 2 keV N^+^ in the Zenith direction (D0 sector). (right) Full coverage at 2 keV N^+^. From BC-SRN-TR-40020-01-00 _MIPA FM Calibration Report

**Fig. 73:**
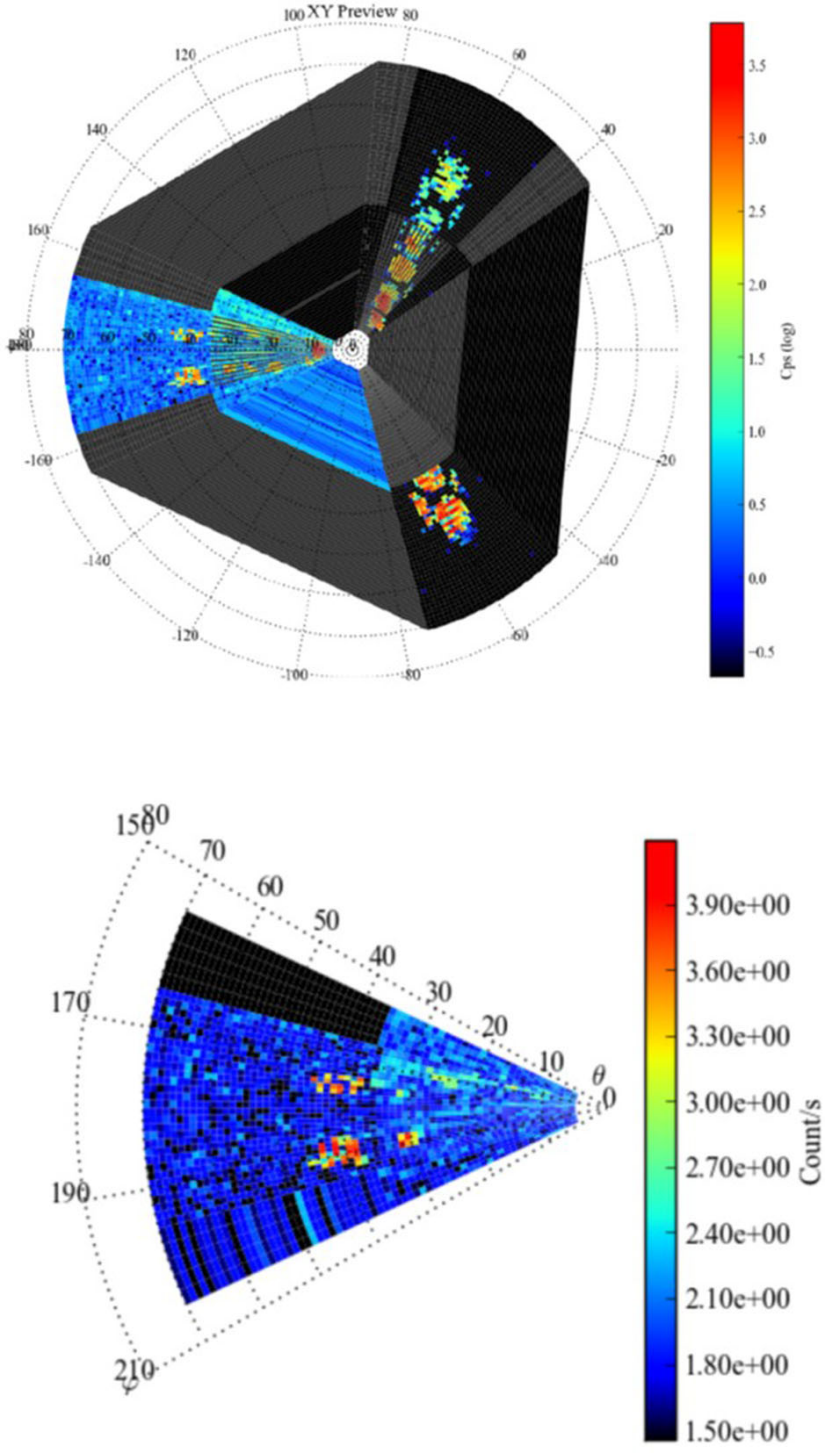
(left) MIPA calibration with 9 keV N^+^ in D0 sector. (right) Full coverage at 9 keV N^+^. From BC-SRN-TR-40020-01-00 _MIPA FM Calibration Report

**Fig. 74:**
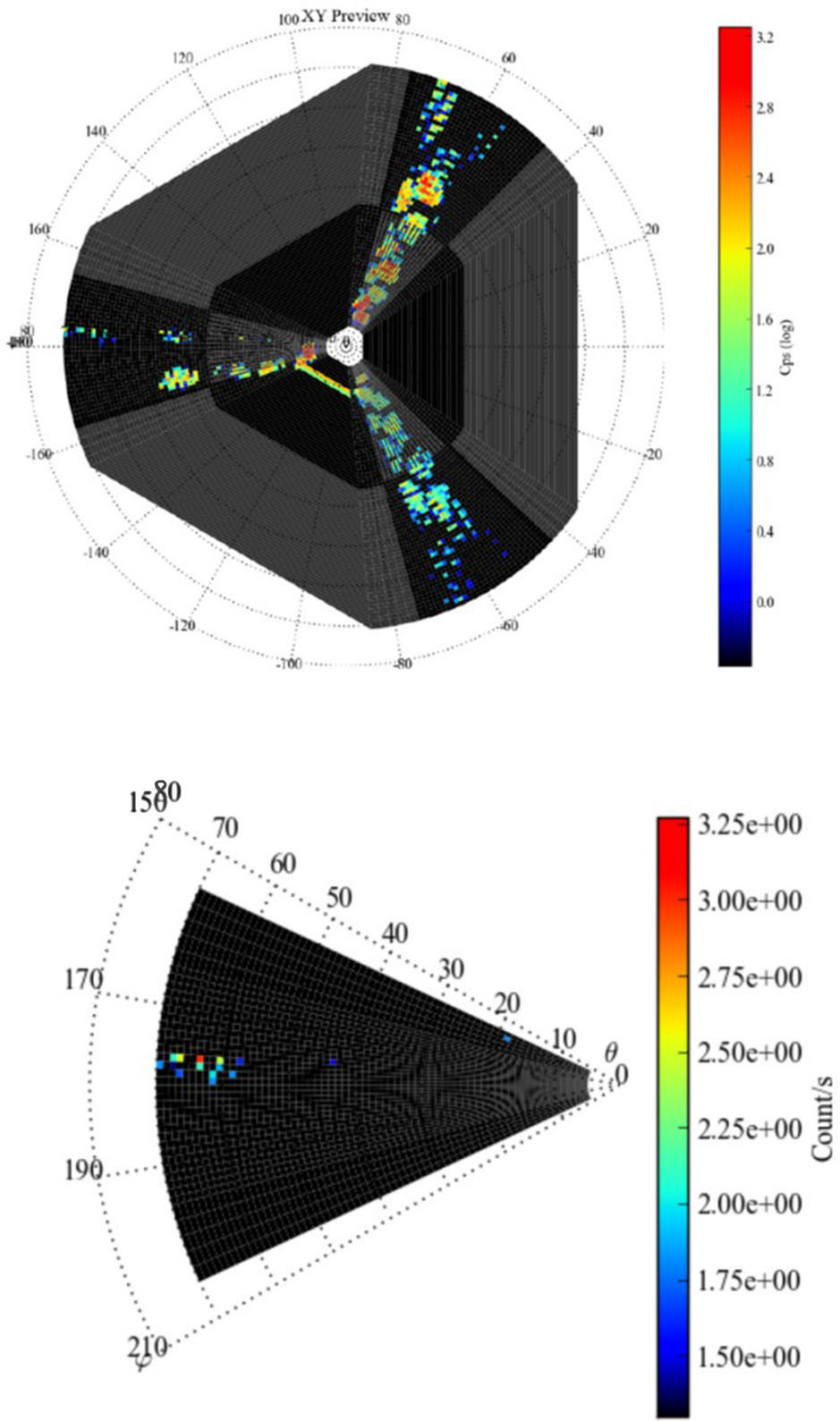
(left) MIPA calibration with 500 eV N^+^ in D0 sector. (right) Full coverage at 500 eV N^+^. From BC-SRN-TR-40020-01-00 _MIPA FM Calibration Report

**Fig. 75:**
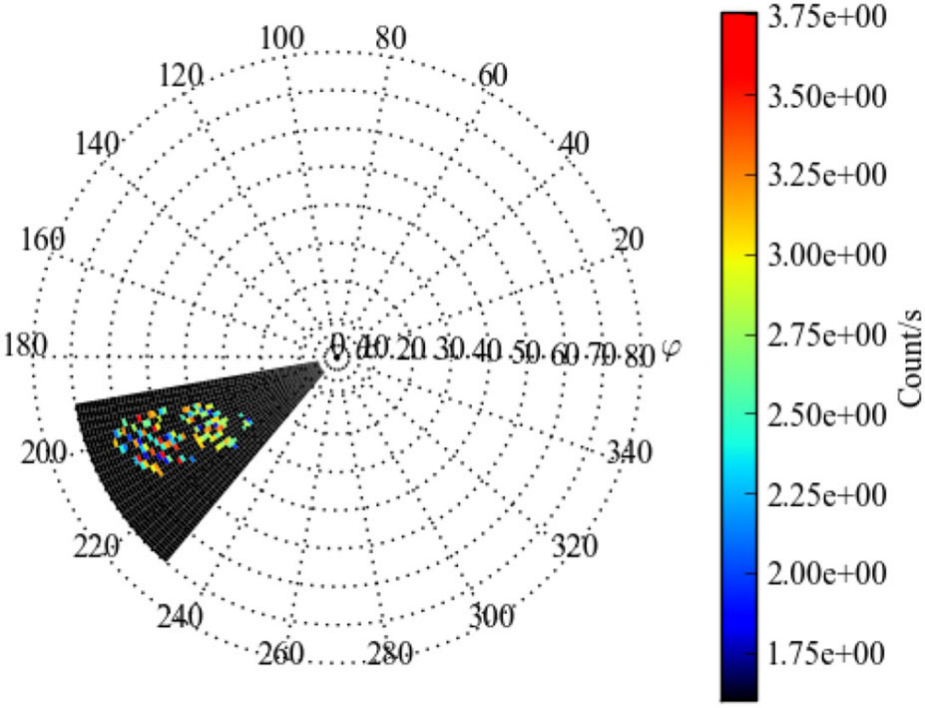
MIPA calibration with 2 keV N^+^ in D12-1 sector. From BC-SRN-TR-40020-01-00 _MIPA FM Calibration Report

In Figs. 72, 73, 74, and 75, some examples of calibration from the MIPA calibration report (BC-SRN-TR-40020-01-00_MIPA FM Calibration Report) are shown.

**Fig. 76:**
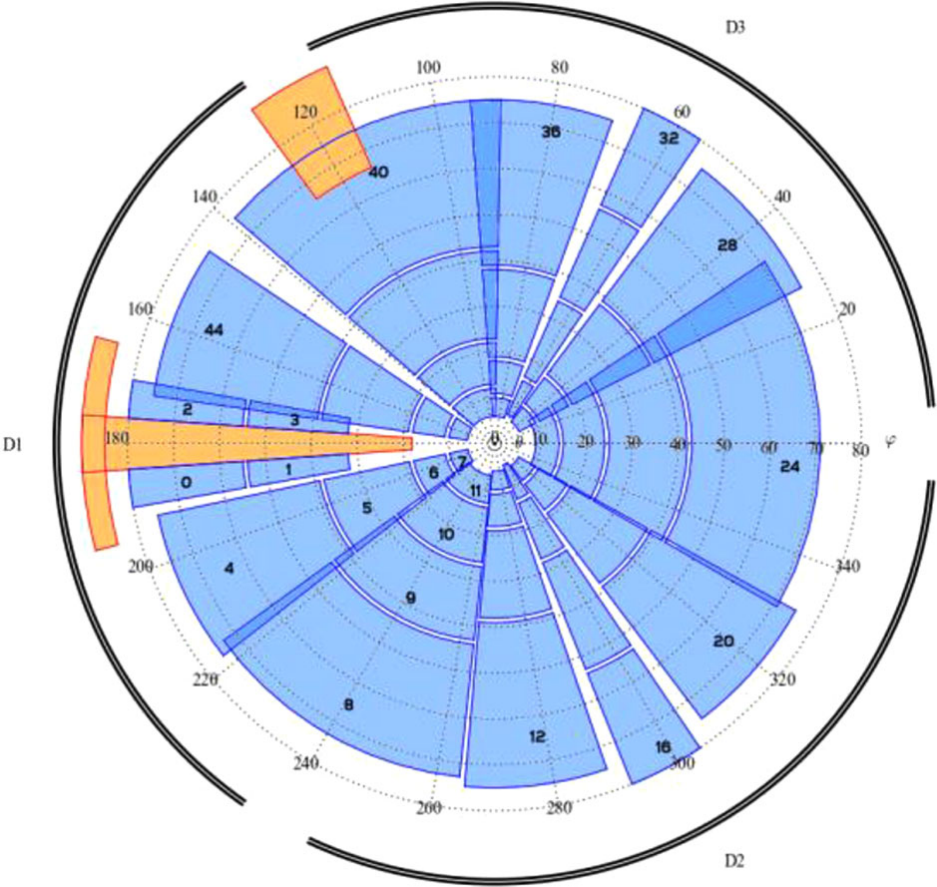
Placement of MIPA pixels based on detailed calibration data processing

Finally, the obtained angular sectors for MIPA are schematized in Fig. 76.

#### Achieved vs. Proposed Performances

MIPA geometrical factor is optimized for covering the very high fluxes (up to 10^9^ part./(cm^2^ s sr)) of the precipitating SW and magnetospheric ions, that eventually induce ion-sputtering process. MIPA with its high energy and time resolution may respond efficiently to abrupt and fast changes of the precipitating ion fluxes.

Given the instrument performances (summarized in Table 10), the parameters listed thereafter refer to the maximum range or best resolution that can be achieved.

- MIPA shall measure ions in the energy range from 15 eV up to 15 keV
- MIPA shall have a limited mass resolution capability to discriminate between protons, alpha particles and heavy ions of planetary origin
- MIPA shall be able to measure the intense fluxes of SW particles without saturating except possibly in extreme events
- MIPA shall measure the particle flux within loss cone
- MIPA shall measure a full energy – angle distribution within 1 min

The achieved MIPA performances with respect to the performances in the SERENA proposal (performance as selected) is compared in Table 14.

**Table 10:** Summary of MIPA performances

Parameter	Required	Actual	Comment
Energy range	500 eV – 10 keV	15 eV – 15 keV	
Energy resolution Δ*E*/*E*	< 30%	7.2%	Coincidence channel
Field of view	2□	80^∘^ × 360^∘^, 24 or 48 angular pixels	Extended to maximum, with the exception of 8^∘^ azimuth × 72^∘^ polar blind sector in the zenith direction in correspondence to the magnetic boom, 13^∘^ × 24^∘^ in the direction of Low gain antenna boom
Angular resolution (FWHM)	Δ*α* < 25^∘^	40^∘^ × 20^∘^ (max pixel) 5^∘^ × 15 (min pixel)	direction dependent
Mass range, amu	1 - 23	1 – 50	
Mass resolution	H, alfa, heavy	*M*/Δ*M* ∼2	
Sampling time		7.81 ms	
Time resolution	< 5 min	18 s, Full Angular–Energy cycle (24A × 96E)	Full FOV coverage achieved, Number of energy steps increased For the (8A × 32E) coverage, the time resolution is 2 sec (fast mode)
Efficiency, *E*		12%	
Geometrical factor, w/o *ε*,	10^−6^≤GF≤10^−4^ cm^2^ sr	∼2⋅10^−6^ cm^2^ sr eV/eV -> 2⋅10^−3^ cm^2^ sr eV at 1 keV	Azimuth dependent
Dynamical range		2⋅10^7^	

### PICAM Calibration and Test

#### PICAM Calibrations Overview

**Fig. 77:**
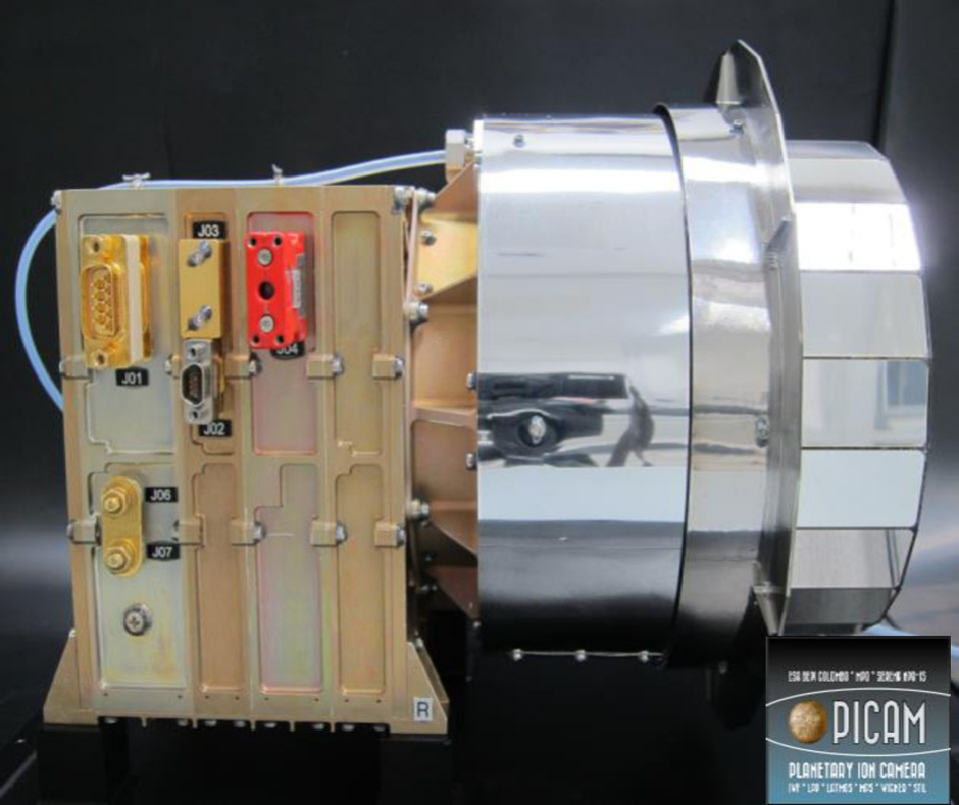
PICAM Flight Model

The PICAM FM is shown in Fig. 77.

The performance of the instrument is mainly influenced by the ion optics, which guides the ions from the entrance slit to the detector. Each of the different parts of the optics has been modelled separately by numerical simulations to characterize its behaviour and to identify its influence on the angular and energy range and resolution, the mapping of the angular distribution of the ions onto the detector, the instrument’s efficiency and geometrical factor, etc. In addition, the behaviour and sensitivity of each individual pixel of the detector had to be tested in order to be able to compare the count rates of the different pixels. Moreover, although PICAM is symmetric about an axis perpendicular to the detector, each sector behaves a bit different due to unavoidable asymmetries induced by the manufacture of the mechanical parts and the assembling of the instrument.

Measurement results obtained in the lab for various beam directions and energies are used to correct for the different performance of the sectors and the corresponding pixels. Inflight calibration by comparison of measurements of PICAM and other instruments, in particular MIPA, will be used for calibration improvements.

#### Lab Calibration

For the measurements, basically two types of ions have been used, He^+^ and $\mbox{N}_{2}^{+}$, and the ion beam was sent through a small aperture before reaching the entrance slit of the instrument. The current of the beam was regularly measured by means of a Faraday cup prior to any changing of the setup. Different ion energies between 200 eV and 2 keV have been used at different angles in the range 25^∘^-90^∘^ polar and 0^∘^-360^∘^ azimuthal, for testing the instrument’s angular, energy and mass resolution.

The calibration was based on a raster of 104 measurement points, which accounts for the symmetrical design of PICAM and the layout of the MCP, which divides the MCP in six sectors with 6 pixels each.

Each measurement at a certain point was repeated with different settings as to the ions species and kinetic energy. More than 10000 measurements have been taken to generate a database for calibration analysis.

The main target was to verify the angular mapping properties.

#### Angular and Azimuthal Coverage and Resolution

In order to check the overall performance and the axial symmetry of the instrument, a beam of 0.2, 0.5 and 1 keV He^+^ ions, respectively, was directed toward the centre of each sector at different elevation angles between 25^∘^ and 90^∘^. For each elevation angle the count rates of each pixel were recorded by operating PICAM in measurement mode 1 (i.e., full angular resolution, without sweeping through the energies. A comprehensive list of modes is given in Table 11). After a time interval of 40-160 sec, the data recording was terminated and identical measurements were repeated for the next elevation angle. In this way the count rates of all pixels according to the incoming ion beam have been obtained.

**Table 11:** Result of statistical analysis on PICAM elevation response for He^+^

Pixel no.	Corresponding elevation		Pixel no.	Corresponding elevation	
	Peak	Range		Peak	Range
0	90.4^∘^	89.0^∘^ - 91.8^∘^	16	55.6^∘^	48.4^∘^ - 64.6^∘^
1	68.1^∘^	64.9^∘^ - 78.2^∘^	17	51.6^∘^	36.6^∘^ - 59.1^∘^
2	87.2^∘^	84.4^∘^ - 90.1^∘^	18	64.9^∘^	56.6^∘^ - 73.3^∘^
3	84.6^∘^	73.8^∘^ - 93.2^∘^	19	32.8^∘^	15.8^∘^ - 49.8^∘^
4	73.1^∘^	69.2^∘^ - 84.2^∘^	20	61.2^∘^	48.8^∘^ - 72.7^∘^
5	67.7^∘^	60.3^∘^ - 75.3^∘^	21	49.2^∘^	38.4^∘^ - 58.2^∘^
6	78.3^∘^	75.4^∘^ - 85.0^∘^	22	47.9^∘^	37.5^∘^ - 58.2^∘^
7	61.4^∘^	50.7^∘^ - 69.7^∘^	23	35.1^∘^	21^∘^ 4 - 9.1^∘^
8	82.7^∘^	72.9^∘^ - 85.0^∘^	24	55.6^∘^	45.9^∘^ - 65.4^∘^
9	69.7^∘^	62.9^∘^ - 83.2^∘^	25	15^∘^	- 19.1^∘^
10	67.2^∘^	59.1^∘^ - 71.4^∘^	26	39.4^∘^	24.1^∘^ - 55.2^∘^
11	62.5^∘^	49.1^∘^ - 65.0^∘^	27	32.9^∘^	17.8^∘^ - 48.0^∘^
12	73.9^∘^	69.1^∘^ - 78.7^∘^	28	32.2^∘^	17.3^∘^ - 47.0^∘^
13	48.5^∘^	37.3^∘^ - 59.6^∘^	29	26.5^∘^	24.3^∘^ - 35.1^∘^
14	73.0^∘^	63.7^∘^ - 79.1^∘^	30	34.6^∘^	16.8^∘^ - 52.4^∘^
15	62.7^∘^	52.6^∘^ - 66.4^∘^			

##### Statistical Analysis Approach

For PICAM data to be used in a scientific context, it is crucial to know the direction of the detected ions. By using the calibration data, it is possible to combine the most frequent activation of a pixel with the set incoming angles. In this way a statistical analysis reveals the most probable source angle for an event on the detector.

For such analysis the following steps are taken:

- All counts are summed up over all the energies channels (typically 32), and also the measurement samples (typically 5 to 6)
- To reduce the noise, the data with the maximum flux below 5000 counts cm^−2^ s^−1^ are removed
- To be able to calculate the probability, all the data are normalised to have the maximum flux equal to 1000000

For each detector pixel, a skymap of all the ions that were detected by that particular pixel, based on azimuth and elevation of the incoming ions, is created. Combining the skymaps of all the pixels, the mapping of the angular distribution of the ions onto the detector is possible.

**Fig. 78:**
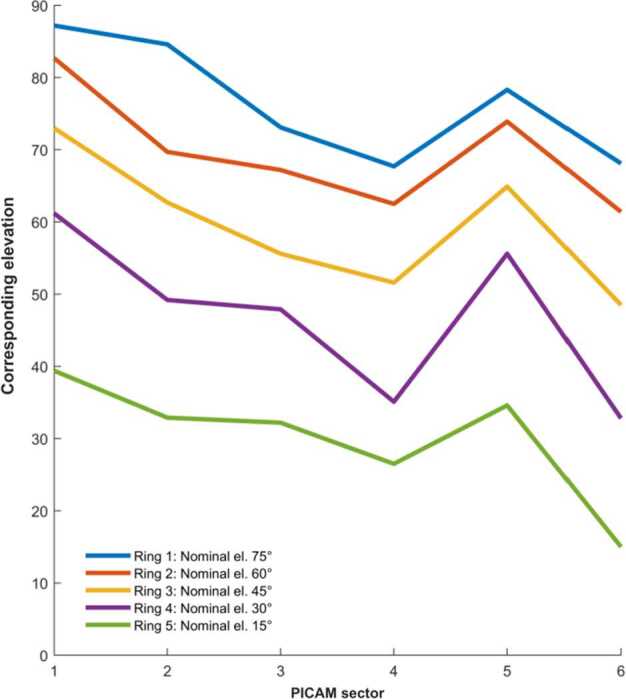
Line plots for each PICAM ring and their elevation peak values (Nominal elevations are 15^∘^ to 75^∘^ from ring 5 to 1)

Figure 78 and Table 11 represent the result of such analysis. The table shows the result of statistical analysis on PICAM elevation response for He^+^. For each pixel, the elevation peak value and the elevation range are shown in degrees. Figure 78 demonstrates the line plots for each PICAM ring and their relevant elevation peak values, where X-axis is the PICAM sector, and Y-axis the corresponding elevation. The nominal elevations for ring 5 to 1 are expected to be 15^∘^ to 75^∘^.

However, as each PICAM sector responds slightly differently from the other, the line plots are not necessarily equal to their nominal values. Instead, these actual values are being used to accurately estimate the most probable source angle for an event on the detector.

##### Computational Estimation of the Beam Geometry on the Detector

Due to possible particle interactions with the instrument parts, the beam of ions may vary from the entrance until arriving at the MCP. This variation could happen in both shape and intensity of the distribution. A study was done on this feature by comparing the real measurement with the calculated shape of the beam.

For such computation, the following steps are taken:

- A 2D normally distributed beam of ions, consisting of 100000 particles is generated.
- The centre of the beam can be at any arbitrary point in X and Y direction on the PICAM MCP plane, with a resolution of 0.2 mm.
- The beam can vary from circular to oval shape, and rotating from 0 to 180 degrees in 2 degree steps

A catalogue of 90× 61× 61×16×16 beam patterns is generated and each pattern is binned to 31 pixels of PICAM. Then the patterns are compared to actual PICAM measurements to find the best beam pattern for a particular measurement. Putting together the beam patterns found for all the comparisons, it is possible to determine the intersection set of them and accordingly the most probable beam geometry on the detector.

**Fig. 79:**
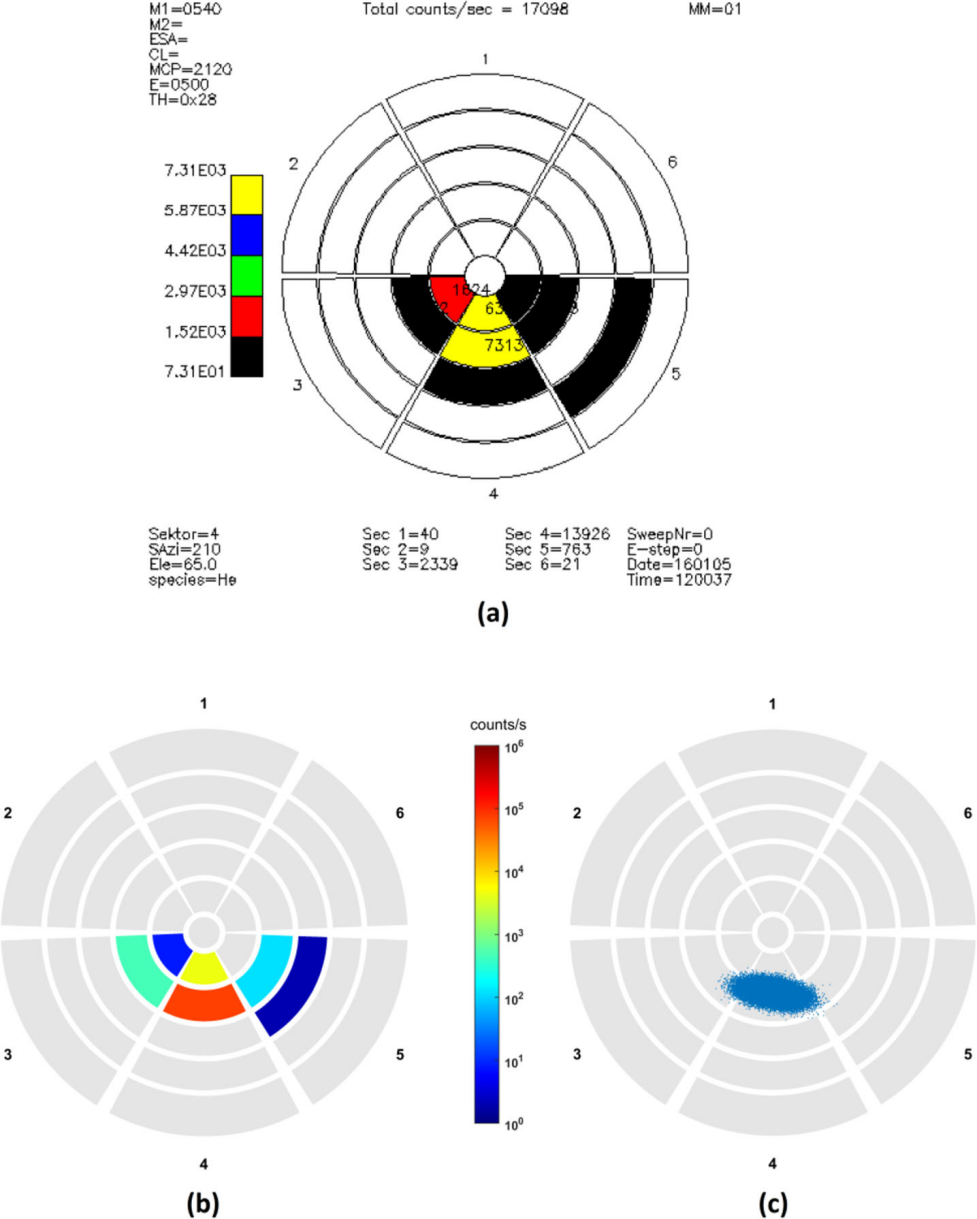
Panel (**a**) shows the azimuth and elevation mapping of the incoming ion beam (He^+^, 500 eV) entering the centre of sector 4 (azimuth=210^∘^ elevation=65^∘^). Panel (**b**) and (**c**): The best fitting of the ion beam pattern, resolved by numerical calculations. Panel **b** represents the actual number of counts from each pixel, when the particles are landing on the detector as shown in panel (**c**)

Figure 79 demonstrates the results of such computations and their comparison. Panel (a) shows an actual measurement for azimuth=210^∘^ and elevation=65^∘^. Panel (c) illustrate the probable ion beam pattern, resolved by numerical calculation. Plot (b) represents the actual number of counts from each pixel, when the particles are hitting the detector. (Note: as shown in panel (c), the beam is more spread in the azimuthal direction. This is consistent with actual measurements where additional counts are detected by the neighbouring pixels, in particular in the inner rings, e.g. Pixel 16.)

#### Energy Resolution

**Fig. 80:**
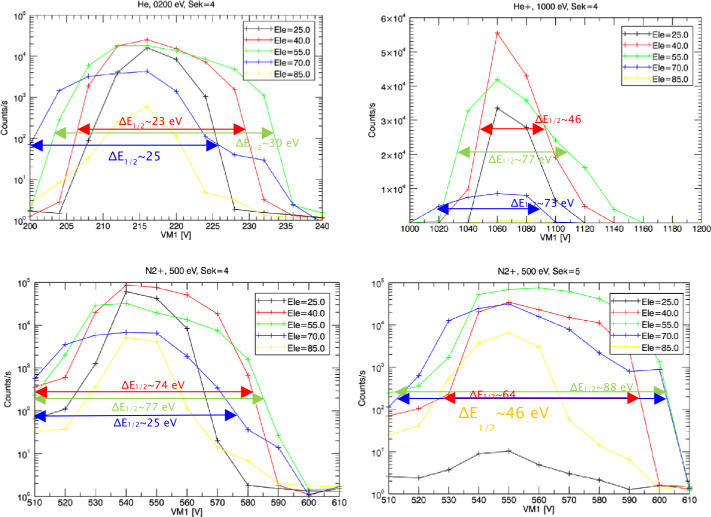
Count rates obtained with the flight model versus M1 voltage for different directions of the incoming ion beam (upper panel: He+ at 200 and 1000 eV, respectively; lower panel: N2+ at 500 eV). The half width $\Delta $E1/2 of the energy distribution for each scan is shown

To determine the energy resolution of the instrument, the count rates for different M1 voltage settings were measured for different species and ion energies. Similar to the angular resolution the results depend on the sector, the elevation angle, and on the ion energy to a certain extent. In all cases, the energy pass band stays below 15% at half intensity. Two examples are shown in Fig. 80.

#### Mass Resolution

##### Single Pulse Mode

TOF-measurements of three different species have been carried out for the flight model: He^+^, N^+^ and $\mbox{N}_{2}^{+}$. The beam was run in a full elevation sweep and was directed toward the symmetry axis of a sector. The ion beam intensity had to be adjusted manually for each species and was different for each measurement, leading also to different count rates.

**Fig. 81:**
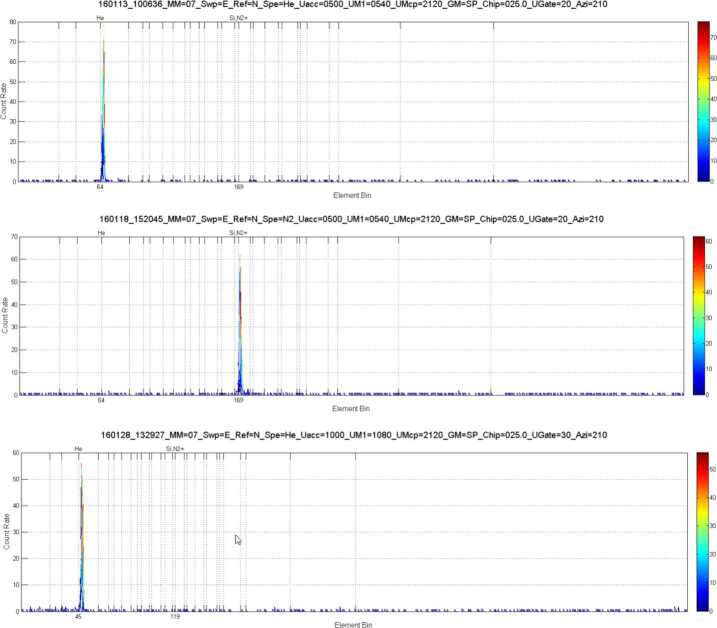
ToF spectra in single pulse mode for two different species (He^+^, $\mbox{N}_{2}^{+}$) obtained at 500 (upper panels) and 1000 eV (lower panel)

Figure 81 illustrates results of time of flight in single pulse mode for ion energies of 500 eV (first panel He^+^, second panel $\mbox{N}_{2}^{+}$) and 1000 eV (last panel He^+^). The species appear as narrow peaks and can clearly be identified.

##### Hadamard Mode

**Fig. 82:**
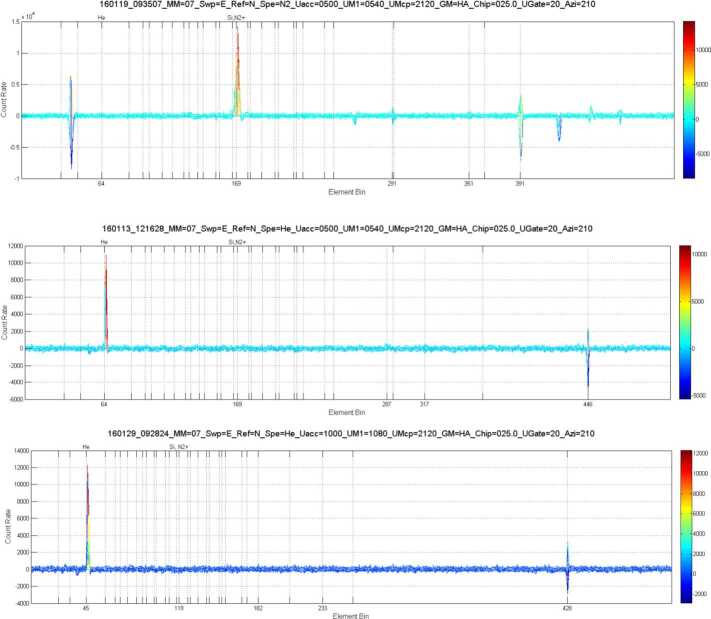
ToF spectra in Hadamard mode for 500 (N2+, He+, first and second panel) and 1000 (He+, third panel) eV ions measured with the flight model

Analogous measurements (500 and 1000 eV ions) were done in Hadamard mode. Examples are shown in Fig. 82. The additional peaks at the left and right end are intrinsic characteristics of the Hadamard mode and do not reflect real ions. However, this feature of the Hadamard mode can be taken into account during data analysis.

##### Transparency and Sensitivity

**Fig. 83:**
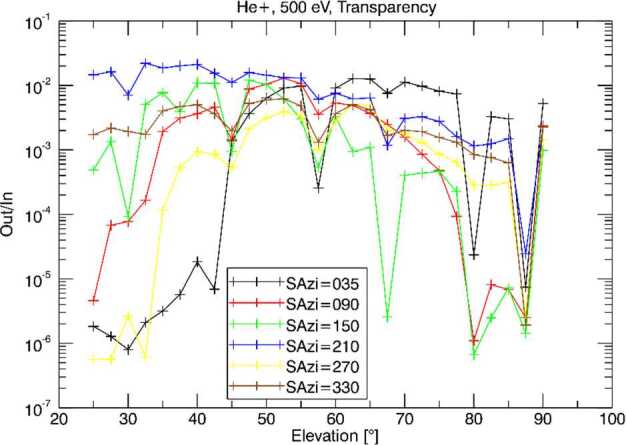
Transparency (fraction of registered to incoming ion flux) for several sectors as a function of elevation angle with the flight model

Figure 83 illustrates the ratio of the recorded flux to the incoming flux for various azimuths and elevation angles. The transparency varies with elevation angles and sectors, but it is acceptably uniform and larger than 10^−3^ over a wide interval of elevation angles (30^∘^-80^∘^) for almost all sectors. At low and high elevation angles, the transparency decreases and the variation among the sectors becomes more distinct. The flux of the incoming ions was estimated by converting the beam current measured in front of the entrance slit of PICAM into the corresponding number of singly charged ions and assuming that all these ions enter the instrument.

#### Summary of PICAM Scientific Performances

Due to technical and resource constraints it is not always possible to achieve the full range or the best resolution of several parameters simultaneously. As an example, the bit rate limitations at MPO prevent the simultaneous transmission at the full energy, spatial, and mass resolution.

- PICAM shall measure ion flux in the energy range from spacecraft potential up to ∼3 keV
- PICAM shall resolve the full energy range in up to 32 steps.
- PICAM shall resolve the full field of view with up to 60 pixels.
- PICAM shall measure complete energy spectra within one to several seconds, but with limited angular and mass resolution
- PICAM shall measure complete energy spectra within one minute, with full angular or mass resolution
- PICAM shall resolve ion masses with a resolution better than 50
- PICAM shall be designed to measure ion mass numbers up to 132.

Table 12 shows relevant PICAM performance parameters.

**Table 12:** PICAM performance parameters

Parameter	Required	Actual	Comment
Energy range	>1 eV - 3 keV	∼10 eV - 3 keV	
Energy resolution Δ*E*/*E*	30%	<15%	
Viewing angle	3-D, 2*π*	3-D, 1.5*π*	Symmetric to the mechanical axis of the sensor
Angular resolution	25^∘^×25^∘^	> (20^∘^×60^∘^)	Sector dependent
Mass range	-	1….132 AMU	Determined by the ion path length of about 26 cm
Mass resolution *M*/Δ*M*, amu	>50	>50	Function of the gating and energy settings
Time resolution	<5 m	1 m	1 to 2 s for mass analysis modes, 0.25 to 1 s for image modes
Sampling time	-	1-320 s	Complete energy spectra with limited angular and mass resolution
Geometrical factor, w/o *ε*, maximum possible base line	≥ 10^−4^ cm^2^ sr	10^−3^ – 10^−5^ cm^2^ sr	Sector and elevation angle dependent; Full statistical analysis pending
Effective Geometrical factor, S Ω ΔE/E	-	10^−4^ – 10^−6^ cm^2^ sr eV/eV	Sector and elevation angle dependent; Full statistical analysis pending

## Operational Modes, Data Rate, and Power Consumption

### Instrument Operating Modes

#### SERENA SCU Operating Modes

**Fig. 84:**
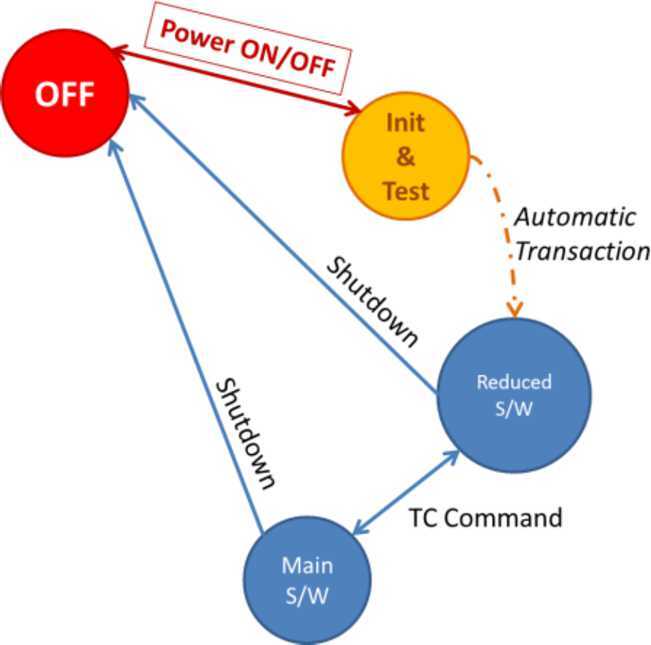
SCU Mode Transition Diagram

The SCU mode transition diagram is shown in Fig. 84. Following the power-up of the instrument, the HW-SW initialisations and tests are carried out. The test results can be provided by requesting specific boot report TM. The Main IDE controller is put into STAND-BY state. The reception of dedicated ON/OFF command from the S/C SpaceWire I/F to the power control register allows to control and power any sub-unit. Compressor unit is a specific sub-unit residing in the SCU itself and it can be powered on as any other Sensor unit. Boot behaviour is described in the next chapters.

The default logic for the SERENA state transitions is that any automatism can be disabled and the modes trajectory is defined by a sequence of ground or OBCP commands. The transition from one state to another (except power off/on) always goes via STAND-BY state.

Regardless of the Compression/DPU state, the following SERENA SCU operational modes can be commanded:

- INIT & TEST automatically entered by power-on/reset.
- STAND-BY: this is the default mode, in which SCU is switched following a reset or a power-up. This mode is realized with the Reduced S/W stored in PROM and can be detected by checking the SCU current mode parameter NSEDS002 (stand-by) or the EEPROM S/W Version parameter NSE0S030 (equal to 0). From this mode, the SCU can be switched to FULL SCIENCE mode by means of ‘Jump TC to Application S/W’.
- FULL SCIENCE (Nominal/High Science Compression). This scientific mode takes full benefit of the availability of the Compression functionality implemented and realized by SCU for MIPA and PICAM science data. It can provide a considerable larger amount of science, even with the same TM LBR/HBR allocation bandwidth of the nominal/high science modes. This mode is realized with the Application SW stored in EEPROM and can be detected by checking the SCU current mode parameter NSEDS002 (full science) or the EEPROM SW Version parameter NSE0S030 (different from 0).

##### INIT & TEST

INIT & TEST concerns the SCU HW-SW initialisation performed at the boot. The result of the initialization is made available in a specific RAM table. If the booting test procedures succeed, SCU switches autonomously to STAND-BY mode and the test results can be provided by requesting specific boot report TM.

##### STAND-BY MODE

This is the default mode provided by the Reduced SW resident on PROM, in which SCU is switched following a reset or a power-up. Only housekeeping telemetry will be supported while SCU is running in STAND-BY mode, but the MIPA sensor functionalities H/K are not supported. The PROM code includes only the mandatory services (in particular the memory management services) without scientific functionalities (as for example, science data compression capabilities). The Reduced S/W resident on PROM allows all the diagnostics operation of SCU.

##### FULL SCIENCE (Nominal/High Science Compression Sub-Modes)

The complete scientific mode is implemented by the Main program that is stored in EEPROM which must be activated through a dedicated jump TC. This scientific mode takes full benefit of the availability of the Compression functionality and it can provide a considerable larger amount of science data.

The way to transfer science data can be commanded in raw mode or in compressed mode. The application S/W must be started via the ‘jump to Application S/W’ through TC ZSE01044 to enter the compressed mode.

There is no automatic switching mode except failures. The SCU science mode should not be split between high and nominal science, as there are several combinations of sensor science modes. Such sensor combinations are not reflected in the high level SCU modes (only one SCU FULL SCIENCE mode is foreseen).

All SCU scientific functionalities are available when the Main program is running.

Each sensor unit foresees at least these basic modes:

- STAND-BY MODE
- SCIENCE MODE(s): one or more science modes with different power and telemetry budget defined for each unit. The “Nominal science mode” indicates the default/preferred one. The “High science mode” indicates the mode with high science performances.
- Other possible modes for calibration/diagnostics.
- When SCU runs in FULL SCIENCE mode, all the sensor modes can be commanded with the following constraint:
  - Not overcome the overall SERENA data telemetry budget (38000 bit/s).
  - Possibly avoid simultaneous full-operational modes for PICAM and MIPA.

#### ELENA Operating Modes

**Fig. 85:**
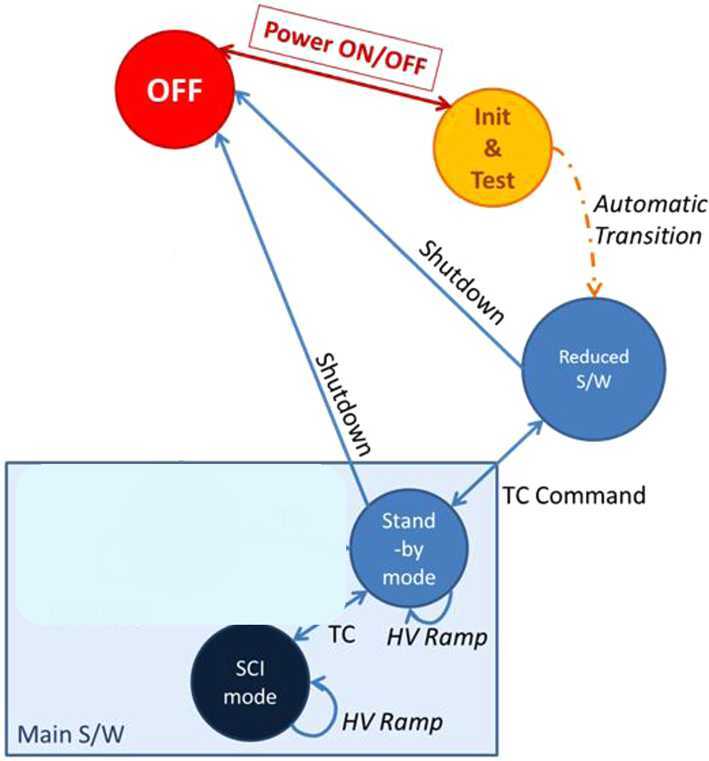
ELENA Mode Transition Diagram

The following ELENA operative modes are foreseen (Fig. 85):

The ELENA S/W is designed to have three operational states:

- REDUCED state implemented with Reduced S/W (Boot sequence)
- STAND-BY state implemented with Main S/W
- SCIENCE mode state implemented with Main S/W.

The REDUCED state supports only a limited set of the S/W functionalities such as:

- Memory management;
- System health check;
- Time update;
- ELENA Housekeeping;
- TC/TM handling (a limited number only).

The Main application S/W implements the Stand-by and Science modes. Once the Main application has started, is possible to switch in such two modes via TCs.

The STAND-BY state supports all the S/W functionalities except science management such as:

- Memory management;
- System health check;
- Time update;
- ELENA Housekeeping;
- TC/TM handling (a limited number only)
- HV management

The SCIENCE state provides the full SW operability:

- Memory management;
- System health check;
- Time update;
- ELENA Housekeeping;
- TC/TM handling.
- HV management
- Science management

In SCIENCE state there are four operative configurations:

- SO_16 Sectors 16 channels (with Standard Histogram type)
- SO_32 Sectors 32 channels (with Standard Histogram type)
- S Sectors 32 channels (with Extended Histogram type)
- R Event by event (Rational or Burst mode)

Note that only modes S and R are considered nominal and will be used in-flight, while SO_16 and SO_32 are backup modes.

#### Strofio Operating

Strofio has four Engineering Modes (Stand-By, Diagnostic, Safe, and Pre-Science), a single Science Survey mode interleaved with four additional science sub-modes (Ion, Molecular, High Energy, and Background modes), and a Calibration Mode. In particular Strofio only has a single science mode throughout the mission. At all times, it will be producing the average 200 bps.

**Fig. 86:**
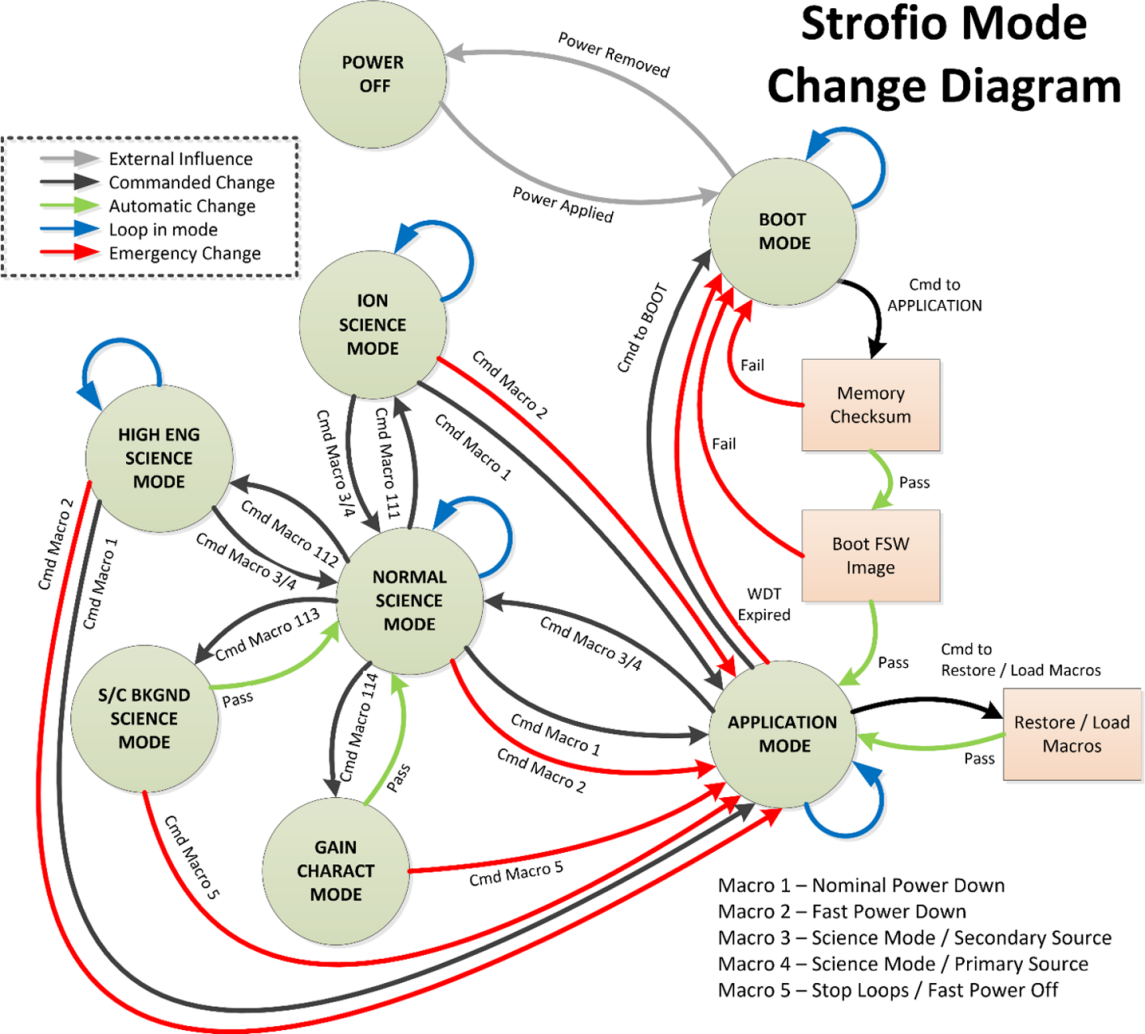
Strofio State Diagram

The different operational modes are provided in Fig. 86.

**Fig. 87:**
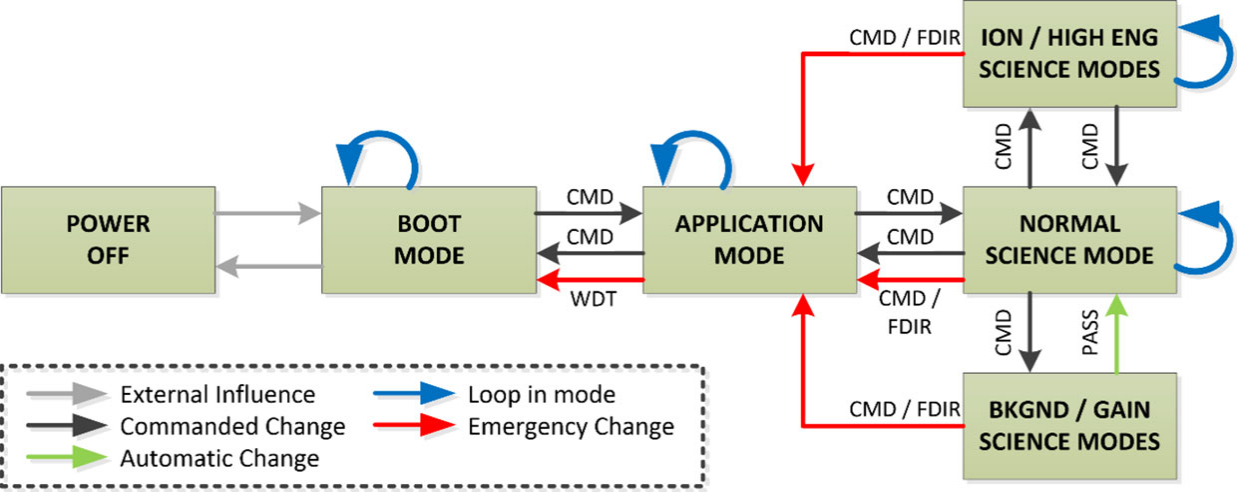
Strofio Mode Transition Diagram

The transitions between modes are summarized in Fig. 87.

#### MIPA Operating

**Fig. 88:**
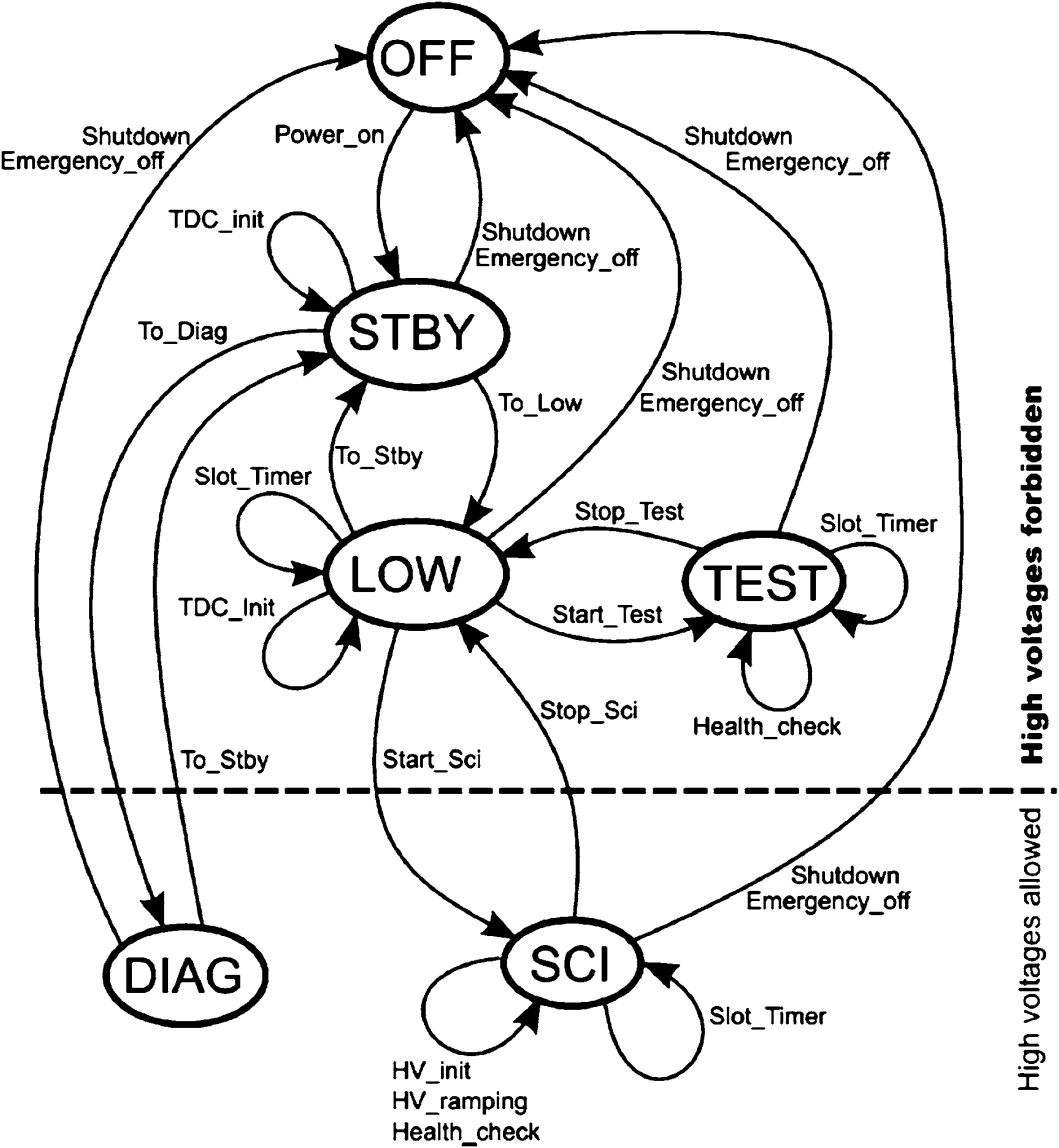
MIPA Mode Transition Diagram realized by SW resident on SCU

The MIPA modes implemented by the SW resident on SCU are shown in Fig. 88.

There are six states defined in the SCU SW modules that control MIPA. The description of these states is reported in Table 13.

**Table 13:** Description of these states of MIPA

SCI State	State description	Allowed actions
OFF	Sensor is powered off	Sensor power on
STBY	Sensor is powered on in low voltage configuration. • No commanding to sensor except direct TC. • No science or HK data acquisition. • High voltages are off	Sensor power off Immediate execution of direct TC Executed at maximum speed: TDC init sequence Macros
LOW	Low voltage mode • HK is periodically acquired • Science data is not acquired	Execution one direct TC once per slot Execution of macro sequence or TDC_Init with one command per slot
SCI	Standard science mode. • HK data is periodically acquired • Science data is periodically acquired. • Science data is processed on SCU if enabled	Switch high voltages on/off Ramp up/down high voltages Switch science data processing on/off Execution one direct TC once per slot Execution of macro sequence with one command per slot (TDC_init is forbidden)
TEST	Ground test mode • High voltages are off • HK data is periodically acquired • Science data is periodically acquired. • Science data is processed on SCU if enabled	Programming the test pulse generator Switch science data processing on/off Execution one direct TC once per slot Execution of macro sequence with one command per slot (TDC_init is forbidden)
DIAG	Diagnostics mode • No automatic HK request • No science data request • No data processing on SCU, data is transparently forwarded	Immediately execute direct TC Any command is allowed

Note that the transition to STAND-BY, LOW and DIAG modes are notified with the generation of relative TM($5,1$) Hello word:

- MIPA Stand-by Hello word TM($5,1$) EID 56577
- MIPA Low Hello word TM($5,1$) EID 56578
- MIPA Diag Hello word TM($5,1$) EID 56579

Transitions from SCI to LOW or from TEST to LOW have a time duration variable and can last up to an entire measurement cycle (maximum worst case is about 2 minutes), therefore in case of these transitions it is recommended to wait for the Low Hello word to be sure that this process has ended.

When SCU enters STAND-BY mode, the sensor is powered ON in low voltage configuration. The STAND-BY mode does not support the following task:

- No commanding to sensor except direct TC;
- No science data acquisition;
- High voltages are off.

**Fig. 89:**
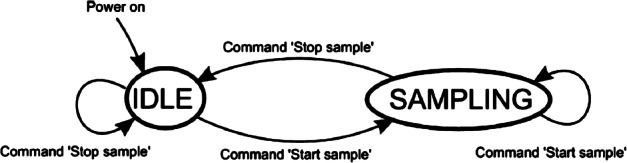
MIPA Low level Sensor Mode

The internal states of MIPA sensor are the low levels states (Fig. 89) controlled directly by the SCU S/W. There are 2 low level states:MIPA internal state changes when issuing mode commands to the sensor.

The description of the 2 internal states is described in Table 14.

**Table 14:** Description of the MIPA two internal states

MIPA internal mode	Description
Idle	TDC not active. No events are written to local RAM
Sample	TDC may be active. TOF events are written to local RAM if they occur

#### PICAM Operating Modes

**Fig. 90:**
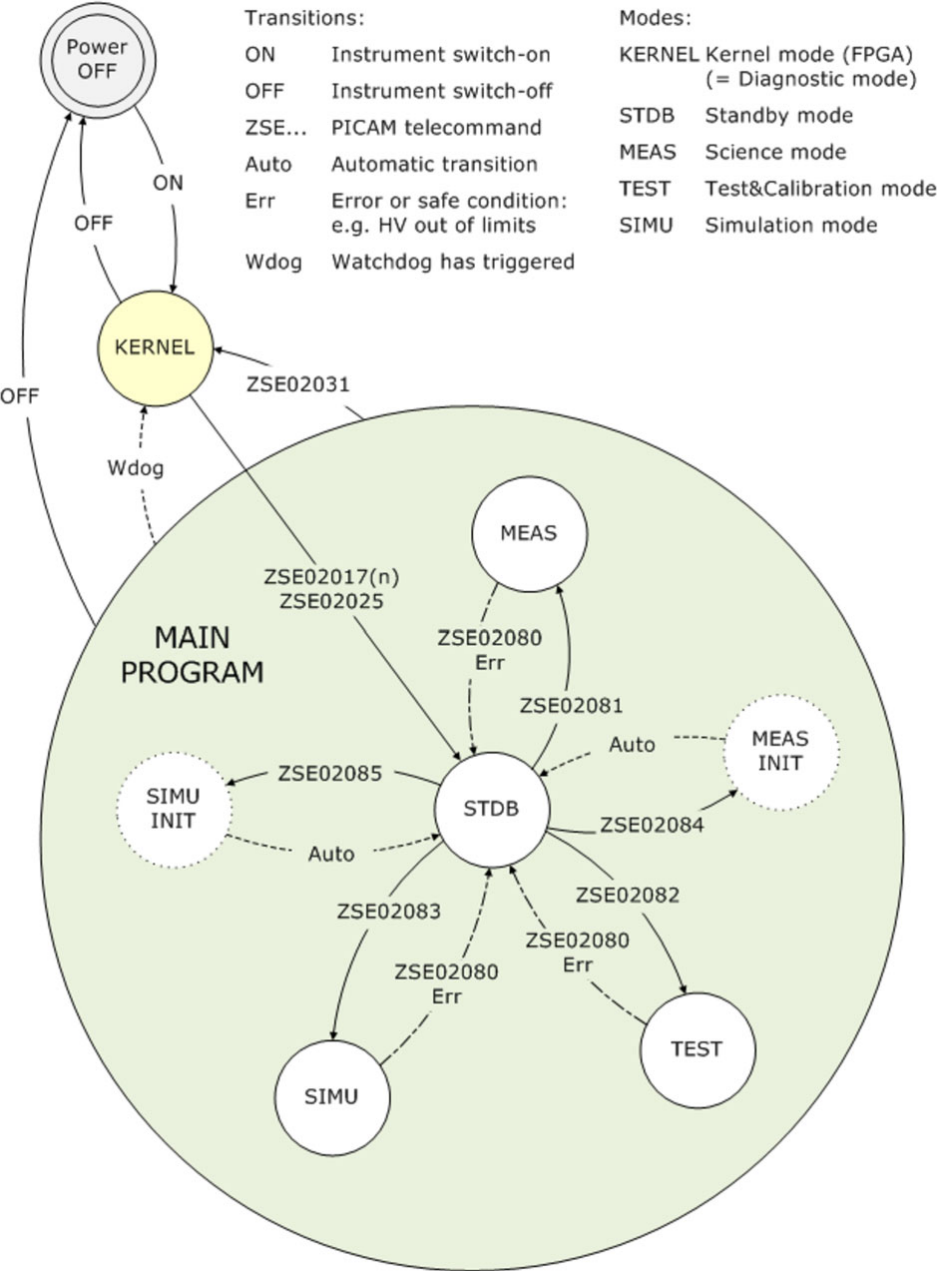
PICAM Mode Transition Diagram

After switch on, the PICAM sensor boots to the so-called Kernel mode, which provides basic tele-command and telemetry handling, including the memory management service. A system check is performed and the content of the first (of two) software module located in the EEPROM is verified. In case of success, the start of the main program can be tele-commanded, which brings the sensor to standby mode. In this mode, no high voltage is active and the PICAM sensor can be set to measurement mode or safely switched off at any time (see Fig. 90).

### SERENA TM Traffic

#### TM Traffic Due to INSTRUMENT HOUSEKEEPING TM($3,25$)

The foreseen housekeeping rate of SERENA instrument is 88 bps subdivided as shown in Table 15.

**Table 15:** Serena HK telemetry (bit/s)

SubSystem	HK size [Bytes]	Generation frequency [s]	HK rate [bps]
SCU	90	20	36
ELENA	86	60	11.5
MIPA	56	60	7.5
PICAM	146 (extended HK) +70 (standard HK)	60	28.8
Strofio	316	600	4.2
SERENA TOTAL			88

For PICAM the HK packets TM rate is in more detail in Table 16.

**Table 16:** PICAM HK packets rate (min, max, peak, average) bit/s

UNIT (bit/s)	MIN (Rate 120 s)	MAX (Rate 4 s)	PEAK (Rate 1 s)	AVERAGE (Rate 60 s)
PICAM	14.4	432	1728	28.8

#### TM Traffic Due to INSTRUMENT SCIENCE TM($21,3$)

The sections hereafter provide the TM traffic tables due to all the SERENA sensors.

For each table the following applies:

NAME = The name of Science mode

INFO = Information about the Science mode

MODE = Number or Extended name of the Science mode

TLM_UNC = Telemetry, uncompressed, in bit/s

TLM_COMP = Telemetry, compressed, in bit/s

POWER_BOL = Power, Begin Of Life, in watt

BOWER_EOL = Power, End Of Life, in watt

TIME = Time Resolution, in second

OTHERS = Ancillary information

#### TM Traffic Due to ELENA SCIENCE

In Table 17 are all the combinations of ELENA science modes but only modes S and R (green highlighted) are considered nominal and will be used in-flight, while SO-16 and SO-32 are backup modes.

**Table 17:** ELENA Science Modes, Power (W) and Telemetry (bit/s)

NAME	INFO	MODE	HISTOGRAM TYPE	POWER_BOL	POWER_EOL	TLM_UNC	TIME
SO-16	Sector Old (16 Sectors)	Sector histogram	Standard	10	11	50	10
SO-32	Sector Old (32 Sectors)	Sector histogram	Standard	10	11	100	10
S	Sector (32 Sectors)	Sector histogram	Extended (with multiplicity info)	10	11	100	10
R	Rational (Ev-by-ev)	Event-by-event	N/A	10	11	8000	1

Power BOL and EOL include only ELENA consumption, without considering the contribution of 4.2/4.6 W (BOL/EOL) for SCU and 0.91/1.00 W (BOL/EOL) added when MIPA is turned ON.

Telemetry rate depends on the integration time applied to the Sector histogram modes. Nominally ELENA integration time is set to 10 sec (default value) but can be increased up to the maximum value of 60 sec. Occasionally 5 sec can be set as well for scientific purposes. So the TM rate changes as following:

- with a resolution of 32 sectors: 10 s integration time (default value) => 100 bit/s; 60 s integration time => 17 bit/s; 5 s integration time => 200 bit/s (worst case).
- with a resolution of 16 sectors: 10 s integration time (default value) => 50 bit/s; 60 s integration time => 8.5 bit/s; 5 s integration time => 100 bit/s (worst case).

Based on 0 s integration time (corresponding to 100 ms) the maximum bitrate for R mode is 16000 bit/s, that is 2 KB/s and 2 TM packets per second. Similarly: 1 s integration time => max bitrate 8000 bit/s, that is 1 KB/s and 1 TM packets per second (default value); 2 s integration time => max bitrate 4000 bit/s, that is 0.5 KB/s and 1 TM packets every 2 seconds.

Note that for R mode the bitrate depends on both the integration time (1 s is default value) and the event flow. The values above are related to the worst cases where the event flow is maximum (up to 1000 ev/s with 0 s integration time, 500 ev/s with 1 s integration time, and so on…). Values are experimental and achieved during dedicated tests performed by using a noise source.

ELENA has only one scientific state and the different science modes can be set by a specific TC (ZSE03016) and a number of parameters. Among these parameters we define:

(A) Integration time in seconds (s): default is 10 s for modes S, SO-32 and SO-16; 1 s for mode R.
(B) Science mode: 1 = Sector histogram; 4 = Event by event.
(C) Sector resolution: 1 = 32 sectors; 2 = 16 sectors.
(D) Test mode: 0 = Sampled data; 1 = Simulated data.
(E) Histogram type: 0 = Standard Histogram type; 1 = Extended Histogram type (multiplicity info).

- From the TM point of view, science resolution is identified by the SID (Structure ID) as follows: SID: 0 = 32 Sectors resolution; 1 = 16 Sectors resolution; 4 = Event-by-Event.

#### TM Traffic Due to Strofio SCIENCE

Strofio TM traffic is indicated in Table 18.

**Table 18:** Strofio Modes, Telemetry (bit/s) and Power (W)

NAME	INFO	TLM_UNC	TLM_COMP	POWER_BOL	POWER_EOL	TIME
N	Nominal	600	200	6.58	7.5	100%

#### TM Traffic Due to MIPA SCIENCE

MIPA modes are shown in Table 19.

**Table 19:** MIPA Modes, Telemetry (bit/s) and Power (W). (mm(nn): nn number of bins actually programmed to the SCU, mm actual resolution the scientist will see)

NAME	INFO	No. of angular pixels	No. of energy steps	No. of mass bins	Counters (1 byte each)	Meas. cycles	TIME (resol)	TLM_UNC	TLM_COMP (Rice comp. factor = 2)	PWR_BOL	PWR_EOL
Mode0	Full	24	96	32	5	1	20	37888	18944	2.6	3.5
Mode1	Full slow	24	96	32	5	6	120	6315	3157	2.6	3.5
Mode2	Monitoring	24	32	2	5	4	80	1792	896	2.6	3.5
Mode3	Minimum	2	32	2	5	4	80	50	25	2.6	3.5
Mode4	Mass resolution 1	1	32	128	5	4	80	473	236	2.6	3.5
Mode5	Plasma dynamics 1 (basic)	24	32	2	5	1	20	2389	1195	2.6	3.5
Mode6	Mass resolution 2	6	32	128	5	4	80	2837	1419	2.6	3.5
Mode7	Mass spectrum from a single pixel	1	96	128	5	5	100	1064	532	2.6	3.5
Mode8	Plasma dynamics 2 (slow)	24	32	2	5	4	80	597	299	2.6	3.5
Mode9	Plasma dynamics 3 (fast)	24	48(96)	2	5	1	10(20)	7168	3584	2.6	3.5
Mode10	Plasma dynamics 4 (ultra fast)	24	32(96)	2	5	1	6.67(20)	7168	3584	2.6	3.5
Mode11	Plasma dynamics 5	24	32	8	5	1	20	3328	1664	2.6	3.5
Mode12	Mass resolution 3	1	32	64	5	4	80	245	123	2.6	3.5
Mode13	Calibration	1	96	128	5	1	0.125(20)	8512	4256	2.6	3.5
Mode14	Raw	n/a	n/a	n/a	n/a	n/a	n/a	16000	8000	2.6	3.5

Power consumption is constant for all MIPA scientific modes.

Conversely the TM data rates depend on the operating mode. Each couple of TM rate (compressed / uncompressed) represents a best estimate according to the following procedure:

1. take the size of the total uncompressed science raw data produced;
2. compress using lin-log compression (fix factor); lin-log compression is always used, except for the RAW (or binning) mode, where MIPA will generates ∼10 times more data than when compression is active;
3. compress using RICE compression.

The estimated compression factor is 2, but this may vary depending on the data itself. RICE compression factor depends on entropy, can be as small as 1 and as high as 4 –> 2 is a best estimate.

MIPA uses 15 modes which are mapped according to MIPA SICD BC-SRN-TN-40000 Iss2 Rev.17 to actual binning parameters and will be used for science planning and in-flight.

- Science modes 0 to 13 are nominal. Science mode 14 “Raw” is a contingency mode to find errors and will not be used routinely.
- Mode 0 “Full” will be used in a few occasions during commissioning and initial operations.
- The difference between Mode 0 and 1 is only the long term average telemetry production.

The maximum instantaneous load on the SCU is the same: it happens simply six times more seldom in mode 1 than in mode 0.

- Mode 9 or 10 are similar to 5 and 6. The higher data rate produced by mode 9 and 10 cannot be well reproduced by test pulses (there is not enough entropy in them) or filament sources (there is not enough energy spread in them).
- Mode 13 “Calibration” is a normal science mode with specially selected binning parameters having a function of calibration. This special mode will be used very seldom for case studies to investigate specific issues that cannot be solved with the standard modes. It is a kind of backup mode.

#### TM Traffic Due to PICAM SCIENCE

SCIENCE PICAM modes are shown in Table 20. The PICAM sensor utilizes a highly flexible measurement concept. The number of measurement modes is limited to 16: six image modes with different time, energy and spatial resolution, five time of flight modes with different time, energy and mass resolutions, and four mixed modes, combining image and time of flight measurements. One mode is dedicated to test purposes. Moreover, each mode can be fine-tuned by means of lookup tables. These tables allow for the modification of the energy steps and their integration time as well as a change of the anode grouping during flight.

**Table 20:** PICAM Modes, Telemetry (bit/s) and Power (W)

Mode #	Description (#Energy steps/#Anode groups/#Bins in TOF spectrum/Duration of one energy sweep [s])	TLM_UNC	TLM_COMP	POWER_BOL	POWER_EOL
1	High Time and Spatial Resolution Image (32/31/1/8)	4176	1044	6.1	6.7
2	High Time and Normal Spatial Resolution Image (32/13/1/8)	1832	458	6.1	6.7
3	High Time and Low Spatial Resolution Image (32/7/1/8)	1064	266	6.1	6.7
4	High Resolution Image (32/31/1/32)	1044	261	6.1	6.7
5	Normal Resolution Image (32/13/1/32)	448	112	6.1	6.7
6	Low Resolution Image (32/7/1/32)	256	64	6.1	6.7
7	High Resolution TOF, High Energy Resolution (32/1/511/32)	16704	4176	6.8/8.6 S/H	7.5/9.5 S/H
8	High Resolution TOF, Low Energy Resolution (16/1/511/32)	8352	2088	6.8/8.6 S/H	7.5/9.5 S/H
9	Normal Resolution TOF, High Energy Resolution (32/1/128/32)	4176	1044	6.8/8.6 S/H	7.5/9.5 S/H
10	Low Resolution TOF, Low Energy Resolution (16/1/64/32)	1044	261	6.8/8.6 S/H	7.5/9.5 S/H
11	High Resolution TOF (32/4/511/32)	66816	16704	6.8/8.6 S/H	7.5/9.5 S/H
12	Full Polar Angle, High Energy Resolution (32/16/128/32)	66816	16704	6.8/8.6 S/H	7.5/9.5 S/H
13	High Energy Resolution (32/12/128/32)	58624	14656	6.8/8.6 S/H	7.5/9.5 S/H
14	Low Energy Resolution (16/13/128/32)	29312	7328	6.8/8.6 S/H	7.5/9.5 S/H
15	Low Mass and Energy Resolution (16/7/64/32)	8352	2088	6.8/8.6 S/H	7.5/9.5 S/H
0	High Resolution TOF, High Energy Resolution (32/31/511/256)	66816	16704	6.8/8.6 S/H	7.5/9.5 S/H

The uncompressed rates refer to the rates at the PICAM-SCU interface. The data rate depends on the size of the science packet and the generation frequency of TM packet for each mode. Data compression is done by the SCU. In this table, a conservative compression factor of 4 is assumed.

In ToF modes and combined modes the power consumption depends on the gate pulsing (Single pulse or Hadamard pulse). The power values contain a margin of about 20% compared to values from ground testing.

### Serena Power Consumption

Table 21 summarizes the “nominal peak scenario” for the operational power of SERENA. Power consumption of each unit for any working configuration is reported.

**Table 21:** SERENA nominal peak power operational scenario^***^

Unit	Nominal (BOL/EOL) [W]		Calibration (BOL/EOL) [W]		Burst (BOL/EOL) [W]		Diagnostic (BOL/EOL) [W]	
*SCU – ELENA*								
SCU	4.2	4.6	4.2	4.6	4.2	4.6	4.2	4.6
ELENA with MIPA OFF	10	11	-	-	10	11	-	-
ELENA with MIPA ON	10.91	12	-	-	10.91	12	-	-
ELENA + SCU with MIPA OFF	14.2	15.6	-	-	14.2	15.6	-	-
ELENA + SCU with MIPA ON	15.11	16.6	-	-	15.11	16.6	-	-
Notes: DCDC for MIPA is hosted on ELENA Main Board. EOL = BOL +10%.								
*Strofio*								
Strofio	6.58	7.5	6.58	7.5	-	-	5	5.5
*PICAM*								
PICAM H orbits (around 50% of orbits)	8.6	9.5	8.6	9.5	8.6	9.5	4	4.4
PICAM S orbits (around 50% of orbits)	6.8	7.5						
Notes: EOL = BOL +10%.H = Hadamard mode, S = Single pulse mode.								
*MIPA*								
MIPA	2.6	3.5	2.6	3.5	2.6	3.5	2.6	3.5
DCDC for MIPA is hosted on ELENA Main Board. EOL = BOL +10%.								
*TOTAL*								
Picam S orbits (SCU + All units)	31.1	35.1	32.9	37.1	32.9	37.1	26.71	30
Picam H orbits (SCU + All units)	32.9	37.1						

^***^Calibration and Diagnostic modes are N/A for ELENA, Burst mode is N/A for Strofio, so that their Nominal values are considered for the computation of the Total power budget

### SERENA Science Operations Scenario

**Fig. 91:**
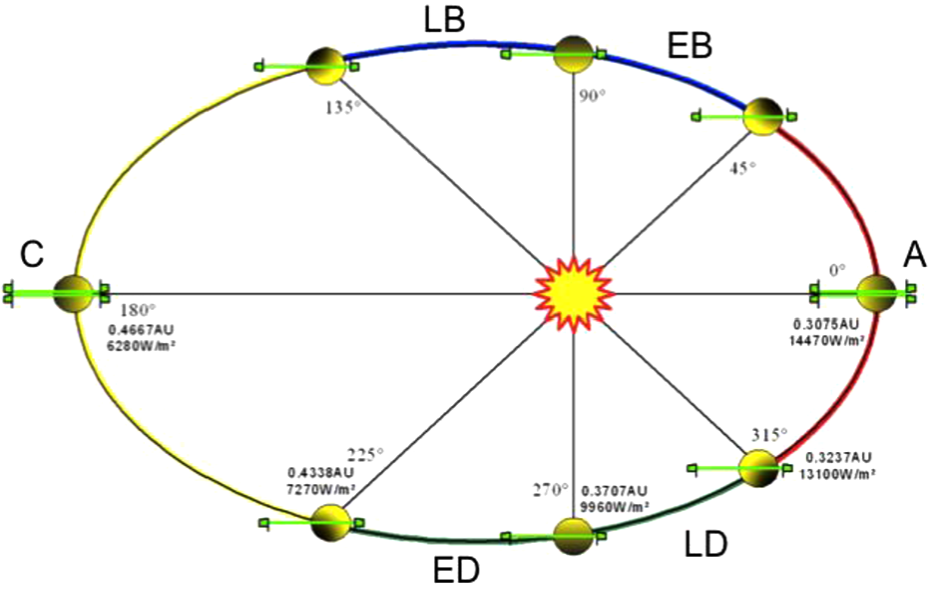
SERENA Science Operations Scenario

The SERENA Science Operations Scenario has been defined for the first Mercury year after insertion. Optimization of the science operation timeline will be done after the first data will be analysed. The SERENA timeline is divided into 6 phases of the Mercury orbit around the Sun (see Fig. 91 and Table 22) and indicates the cross reference between the subunits, the scientific objectives (reported in Sect. 2) and the orbit of MPO (both apo- and peri-herm sides are considered for each Mercury phase).

**Table 22:** SERENA Science Operations Scenario

	MERCURY POSITION	MPO POSITION
Aa	A Perihelion 315^∘^-45^∘^	apoherm
Ap	A Perihelion 315^∘^-45^∘^	periherm
EBa	EB 45^∘^ - 90^∘^	apoherm
EBp	EB 45^∘^ - 90^∘^	periherm
LBa	LB 90^∘^ - 135^∘^	apoherm
LBp	LB 90^∘^ - 135^∘^	periherm
Ca	C Aphelion 135^∘^-225^∘^	apoherm
Cp	C Aphelion 135^∘^-225^∘^	periherm
EDa	ED 225^∘^ - 270^∘^	apoherm
EDp	ED 225^∘^ - 270^∘^	periherm
LDa	LD 270^∘^ - 315^∘^	apoherm
LDp	LD 270^∘^ - 315^∘^	periherm

Table 23 shows a reduced set of instrument modes used for the planning of baseline science observations. During operations, more instrument modes are likely to be used to further focus on specific science objectives, react to results from prior measurements and to adapt to changes in power and/or data volume constraints.

**Table 23:** SERENA Instrument modes reduced set

	Legend of the operating modes
STROFIO	N = Nominal
ELENA	S = Sectors 32 “Extended Histogram type”
	R = Rational “Event-by-Event”
PICAM	1 = IM_HT_HR31 (High Time and Spatial Resolution Image)
	8_S = MC_HR511_LE_S (High Resolution TOF, Low Energy Resolution - Single pulse)
	8_H = MC_HR511_LE_H (High Resolution TOF, Low Energy Resolution - Hadamard pulse)
	13_S = MD_NR128_HE_S (High Energy Resolution - Single pulse)
	13_H = MD_NR128_HE_H (High Energy Resolution - Hadamard pulse)
MIPA	1 = Full slow
	5 = Plasma dynamics 1 (basic)
	6 = Mass resolution 2

The main science objectives of the SERENA Instrument suite are shown in Table 24. These objects address to the following science topics:

- Exosphere composition and spatial distribution and dynamics
- Search for exo-ionosphere and its relation with neutral atmosphere
- Surface release processes.
- Atmosphere/magnetosphere exchange and transport processes
- Escape, source/sink balance, geochemical cycles

These high level goals are broken down into finer granularity to represent the observations currently considered in the baseline operations plan. A full overview of Serena science goals is reported in Table 1.

**Table 24:** SERENA science objectives legend

	Legend of the science objectives
1	Chemical and elemental composition of the exosphere
2	2a. Neutral gas density asymmetries (Latitude)
	2b. Neutral gas density asymmetries (Day/night)
	2c. Neutral gas density asymmetries (Dawn/dusk)
	2d. Neutral gas density asymmetries (Altitude)
	2e. Neutral gas density asymmetries (Temporal variation)
3	Exo-ionosphere composition
4	Exo-ionosphere spatial and energy distribution (Temporal variation vs Solar Wind)
5	5a. Plasma precipitation rate (SW)
	5b. Plasma precipitation rate (SW distribution in the inner magnetosphere)
	5c. Plasma precipitation rate (Heavy ions)
6	6a. Surface emission rate and release processes (SW - sputtering emission)
	6b. Surface emission rate and release processes (SW - back-scattering emission)
	6c. Surface emission rate and release processes (Time-averaged ion-sputtering emissivity of surface features)
	6d. Surface emission rate and release processes (Surface MIV)
	6e. Surface emission rate and release processes (PSD)
7	7a. Particle loss rate from Mercury’s environment (SW sputtering)
	7b. Particle loss rate from Mercury’s environment (Exospheric charge-exchange)
	7c. Particle loss rate from Mercury’s environment (Loss of planetary ions)

The science goals per key instrument are as follows:

- ELENA 5a, 5c, 6a, 6b, 7a
- MIPA 4b, 5a, 5b, 5c, 6a, 6b, 7b
- PICAM 3, 4a, 4b, 5b, 5c, 7b
- Strofio 1, 2a, 2b, 2c, 2d, 2e, 6b, 6c, 6d

**Fig. 92:**
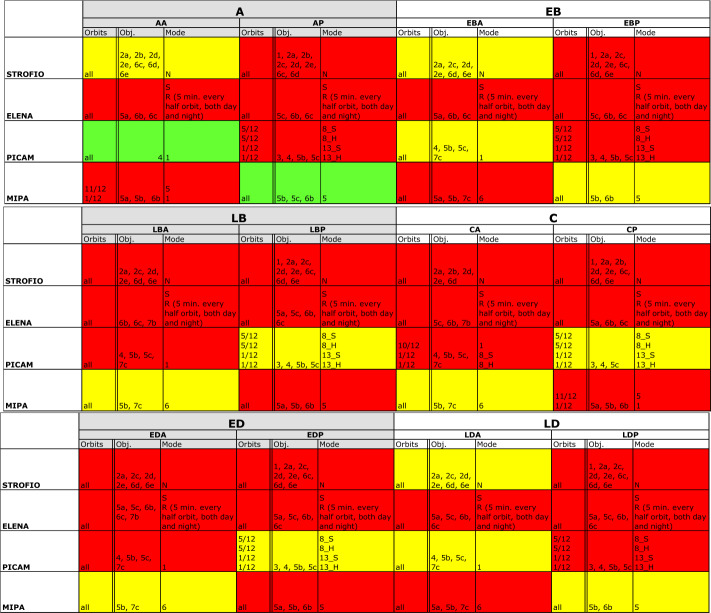
SERENA science goals and their priority per orbit phase and instrument. Red: high priority, Yellow: normal priority. Green: low priority

Figure 92 presents a color-coded 3-level priority of each set of science goals per instrument and orbit phase. In each orbit phase, the science objectives are further divided into apoherm and periherm arcs (for example: A$\mathbf{A}$ = Perihelion/Summer season $\mathbf{A}$poherm arc; A$\mathbf{P}$ = Perihelion/Summer season $\mathbf{P}$eriherm arc; etc...)

## Cruise Phase SERENA Science Operations

Before arriving at Mercury in December 2025, BepiColombo will flyby Earth (04/2020), Venus (10/2020 and 08/2021) and six times Mercury.

As extensively presented by Mangano et al. (2020), during the interplanetary cruise phase, orbit changes are achieved by the planetary flybys and by the Solar Electric Propulsion System (SEPS). During the cruise, the radiator panel (-Y-axis) points anti-sunwards, thus +Y-axis towards the Sun. Only small deviations from this pointing – described by solar aspect angle (SAA), that is the angle between +Y-axis and the Sun direction projected onto the X/Z plane – are allowed during the cruise phase.

The BepiColombo cruise configuration does not allow full instrumental science operability, due to the location of the payload within the Mission envelope (Benkhoff et al. 2020). Concerning SERENA, MIPA and PICAM may operate, since they are located at the border of the radiator plane, out of the BepiColombo elements stack, whereas ELENA and Strofio are fully blind.

### BepiColombo Science Objectives During Cruise Phase

During the cruise phase, solar activity will rise from a low level to eventually its maximum around 2025, so that interplanetary processes could be studied under different conditions over the solar cycle. In fact, even a limited number of instrument operations could contribute to a wide range of scientific cases, as for example (see Mangano et al. 2020):

- *in-situ* SW observations, with special regard to the study of Coronal Mass Ejections (CMEs), Co-rotating Interaction Regions (CIRs), high-speed SW streams, Heliospheric Current Sheet and Solar Energetic Particles (SEPs). The ratio between Fe and H, i.e., the so-called First Ionization Potential (FIP) bias, used to determine the location of SW source based on the abundance of the elements, can be measured by the PICAM and MIPA ion sensors of the SERENA package;
- measurements of plasma composition, of SW ion flux & density, and of magnetic fields in the vicinity of Venus and Mercury during flybys;
- coordinated science observations together with other spacecraft in Earth orbit and in the inner Solar System, e.g., Akatsuki, Parker Solar Probe, SDO, Proba-2, Hisaki, SOHO, Solar Orbiter and JUICE, to provide new measurements and/or additional vantage points;
- observations of InterPlanetary Scintillations (IPS) coordinated with Earth-based measurements to provide information on SW density.

In addition, other studies could benefit of the BepiColombo cruise phase, such as:

- analysis of cometary composition, and detection of dust particles of different origins;
- monitoring the local radiation background due to bombardment by energetic particles of Galactic Cosmic Rays;
- Gamma Ray Burst (GRB) detection and localization;

and, of course, the above mentioned superior solar conjunction measurements to test general relativity.

### SERENA -MIPA and -PICAM Science Topics During Earth and Venus Flyby

During the whole cruise, the SERENA ion sensors boresights are perpendicular to solar wind direction, so, despite the wide FoV of both sensors, it is hard to see the cold solar wind during interplanetary cruise. On the contrary during the flybys the plasma distribution could be wider, so ion detections will be feasible.

The BepiColombo Earth flyby has occurred recently. On April 10, 2020 BepiColombo flew inside the Earth’s magnetosphere crossing several regions like Magnetosheath, Low Latitude Boundary Layers, Plasma Sheet, Ring Current and Radiation Belts.

In some of these regions MIPA and PICAM were operated, with special interest in units and sectors intercalibrations, to be performed during the magnetosheath and plasma sheet crossing. In the magnetosheath H^+^ distribution is wide and in the plasma sheet H^+^ and O^+^ distributions are almost omnidirectional and with a significant flux in the SERENA sensors energy range, so that ion detection is feasible over a wide range of angular sectors of the two SERENA sensor FOVs. In the inner regions (< 6 Re), SERENA will not be operated for reducing the total dose at the detectors. The MAG data allowed the interpretation of the ion distributions observed by SERENA. At present, the preliminary analyses of the data showed that both PICAM and MIPA were both able to detect the magnetosphere regions crossings and related plasma regimes, like bow shock, magnetopause, magnetosheath, and plasma sheet.

On 15 October 2020 and 11 August 2021 two Venus fly-bys will occur, in order to use this planet to deviate the spacecraft towards Mercury and use the slingshot effect to brake it. However, these two flybys can also be used to study the Venusian environment.

In the first flyby, the spacecraft will approach the planet from the solar direction, over the dayside. The closest approach (CA, 10681 kn) occurs above the evening terminator of the planet, and then the spacecraft moves away from the planet along the anti-solar direction, over the night-side. The spacecraft will cross the bow shock around the time of closest approach, and will enter the ionotail about 40 min later, at a distance of 5R_V_. This offers the opportunity to study the induced magnetotail dynamics. As discussed by Mangano et al. (2020), similarly to the Earth, during this flyby the SERENA ion sensors will have the chance to observe particle flow within plasmoids travelling tailward, with a heavy ion flux of about 10^8^ [amu/(cm^2^ s)]. These relatively high heavy ion fluxes should allow PICAM to measure the magnetospheric ions during the first flyby.

In the second flyby, the spacecraft will approach the planet from its night side; CA (598 km) will occur again near the evening terminator. With respect to the first flyby, it will be slightly shifted toward late afternoon side. Then BepiColombo will move away from Venus towards the morning direction. In this phase, close-up observations can be performed from early night side to evening terminator at low latitudes, with similar strategy as for the first flyby. The composite will probably enter the ionosphere for a few minutes during closest approach, at an altitude of 740 km where BepiColombo will be inside the magnetic pileup boundary (MPB). If PICAM and MIPA will have the spacecraft ram vector in their FOVs, the two SERENA sensors will probably encounter high fluxes of planetary heavy ions. PICAM is optimized to observe higher fluxes of ions with energies less than the SW one. This means that during the second flyby PICAM would be able to analyze the ion composition both in the ionotail and during the ionosphere passage. With the good mass resolution of PICAM, the sensor may deliver the first quantified observation of minor ion escape from Venus as C+, N+, CO+ or CO2+.

Another interesting observation that can be made by BepiColombo is the study of the plasma turbulence (at MHD and kinetic scales) and the identification of different plasma wave modes in the different regions of the Venus plasma environment. These studies will be further consolidated if the plasma data from MIPA and PICAM will be available.

Cruise phase further considerations may be found in Mangano et al. (2020).

## Summary

In this paper we have extensively illustrated the major features of the SERENA suite on-board BepiColombo Mercury Planetary Orbiter, composed by four units: ELENA, Strofio, MIPA and PICAM, together with the internal System Control Unit, SCU. All the possible science objectives of SERENA have been analysed and compared to the actual performances of the four units. Moreover, the instrument calibration activity has been described, so that the actual status of the units flying onboard BepiColombo have been emphasized. The technical and scientific system philosophy has been faced and the various operational modes have been described. Finally, the scientific operations have been described versus the planet orbital phases around the Sun. In the end, some possible scientific objects to be reached during the cruise phase have been traced, in relation with MIPA and PICAM, the only SERENA units which may operate before the BepiColombo elements separation to be executed at the start of the operativity phase around Mercury.
